# Migratory Insertion of CO into a Au–C Bond

**DOI:** 10.1021/jacs.2c10432

**Published:** 2022-10-25

**Authors:** Jamie A. Cadge, Paul J. Gates, John F. Bower, Christopher A. Russell

**Affiliations:** † School of Chemistry, University of Bristol, Cantock’s Close, Bristol, BS8 1TS, United Kingdom; ‡ Department of Chemistry, University of Liverpool, Crown Street, Liverpool, L69 7ZD, United Kingdom

## Abstract

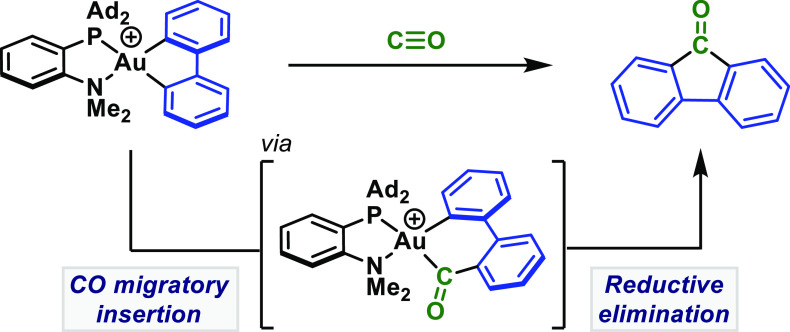

A MeDalPhos-ligated gold(III) metallafluorene complex, generated via C–C oxidative addition of biphenylene, reacts with CO to produce 9-fluorenone. Experimental and computational studies show that this proceeds via a hitherto unknown migratory insertion of CO into a Au(III)–C bond. This process is more energetically challenging compared to other M–C bonds, but once achieved, the product is comparatively stable with respect to retro-carbonylation. Exploiting migratory insertion of CO into Au–C bonds may extend the range of products that are accessible using gold chemistry.

CO is of great importance as an atom economical C-1 building block.^1−3^ Metal-catalyzed carbonylation using CO is exploited on vast scale in both alkene hydroformylation^4^ and the Cativa process.^5^ On smaller scale, complex molecules are routinely accessed via metal-catalyzed carbonylation, with examples including the synthesis of esters by Pd-catalyzed carbonylation of aryl halides,^3c^ and oxidative addition triggered carbonylations of strained C–C bonds (Scheme 1A).^6^ A unifying mechanistic feature of these processes is migratory insertion of CO into a M–C bond. This fundamental organometallic step has been demonstrated at a wide range of transition metal centers;^7^ however, to our knowledge, this process is not part of the repertoire of Au–C bonds.

**Scheme 1:**
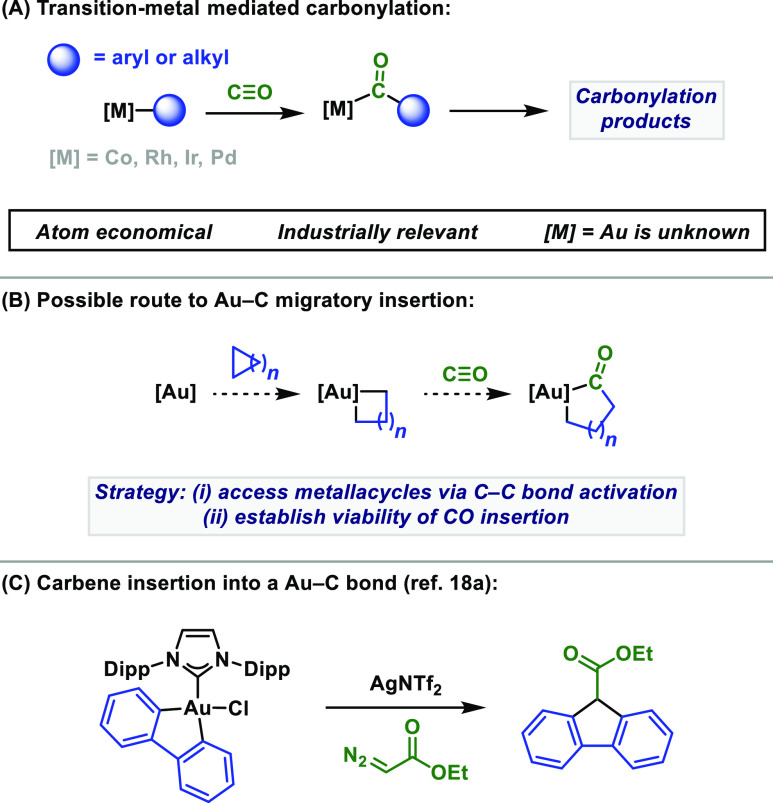
Migratory Insertion Processes

There is growing interest in the development of Au(I)/Au(III)-mediated redox transformations that exploit weak internal oxidants (e.g., Ar–X, strained C–C bonds).^8^ Broadly, these efforts seek to achieve typical late transition metal reactivity (“Pd-like”) at Au centers. This is significant because Au can offer enhanced biocompatibility, unique functional group tolerance, and reactivity which differs from that of Pd; e.g., the enhanced Lewis acidity of Au-centers (vs Pd) leads to the intriguing observation that oxidative addition of aryl halides is faster for more electron-rich substrates.^9^ Of relevance to this study are processes involving oxidative addition of strained C–C bonds.^10^ Bourissou, Amgoune and co-workers reported that a Au(I) center ligated by a rigid, narrow bite-angle carboranyl diphosphine ligand reacts with biphenylene to give a Au(III) metallacycle (*vide infra*).^11^ Related processes have been described using other LAu(I) moieties, including L = NHC,^12^ cyclic (alkyl)(amino)carbene (CAAC),^13^ hemilabile *P*,*N* (MeDalPhos),^9c^ and *C*,*N*-ligands.^14^ These auracycles are readily accessible compounds containing Au(III)–C bonds, which we suspected might serve as excellent model systems for studying migratory insertion of CO (Scheme 1B).

Several migratory insertions into Au–E bonds (e.g., E = H, O, Si) have been observed,^15^ and insertion of CO into a Au–OH bond has been proposed as a possible pathway leading to a Au–H species.^16^ However, examples where E = C are restricted to insertions of alkenes,^17^ carbenes,^18a^ or electrophilic species such as SO_2_.^18b−18d^ For example, Toste and co-workers demonstrated carbene insertion into an IPr-ligated Au(III) metallafluorene complex to give esters (Scheme 1C).^18a^ In this study, we demonstrate the hitherto unknown migratory insertion of CO into a Au–C bond by exploring the aforementioned auracycles (Scheme 1B). This observation is of fundamental interest, and may underpin the development of future carbonylation reactions by providing another “Pd-like” mechanistic step for Au(I)/Au(III) catalysis. During the course of this work, Hashmi and co-workers reported that an *in situ* formed CO-bound IPr aurafluoroene complex *does not* undergo migratory insertion of CO, with intermolecular attack of nucleophiles onto the CO moiety occurring instead.^19^ This observation contrasts the results presented here and highlights the significance of our findings.

Despite the challenging nature of the Au(I)/Au(III) couple,^20^ several small bite angle (*approx.* 90°) bidentate ligands^9a,9b,9e,9f^ alter the properties of the Au(I) center sufficiently to facilitate oxidative additions (e.g., of aryl halides or strained C–C bonds). Indeed, we showed that 5,5′-difluoro-2,2′-bipyridyl ligated Au(I) complex **1** is effective for the oxidative addition of aryl, alkenyl, and alkynyl C–I bonds.^9b,9e^ To ascertain the feasibility of related C–C oxidative additions, a 1,2-dichlorobenzene solution of **1** was reacted with biphenylene (**2a**) at 70 °C under static vacuum (Scheme 2A).^21^ This resulted in approximately 20% conversion to a new species where the chemical shift of the ligand ^19^F reporter nuclei had shifted downfield (δ_F_ = −114.5 ppm vs −117.8 ppm for **1**), as expected for Au(III) complex **3**. Mass spectrometry provided further support for the formation of **3** {*m*/*z* 541.0786 for [M–NTf_2_]^+^ (calcd. 541.0791)}. Efforts to improve the conversion of **1** to **3** were unsuccessful, and so we investigated C–C oxidative additions and carbonylations of other strained rings. Exposure of cyclopropanes **2b**–**e** to **1** led to decomposition, with no evidence for the formation of C–C oxidative addition products.^22^ Similar results were obtained using MeDalPhosAuCl or IPrAuCl in place of **1**.^23^ The contrasting reactivity of 3-membered **2b**–**e** versus **2a** was investigated computationally (see Supporting Information (SI)), leading to two key findings. First, 4-membered auracycles generated from oxidative addition of cyclopropane-based substrates are thermodynamically *disfavored* compared to 5-membered variants.^24^ Second, auracycles containing two Au–C(*sp*^2^) bonds are *favored* compared to those possessing Au–C(*sp*^3^) bonds. Thus, oxidative addition of **2a** to **1** is feasible, whereas **2b**–**2f** do not lead to the corresponding auracycles.

**Scheme 2:**
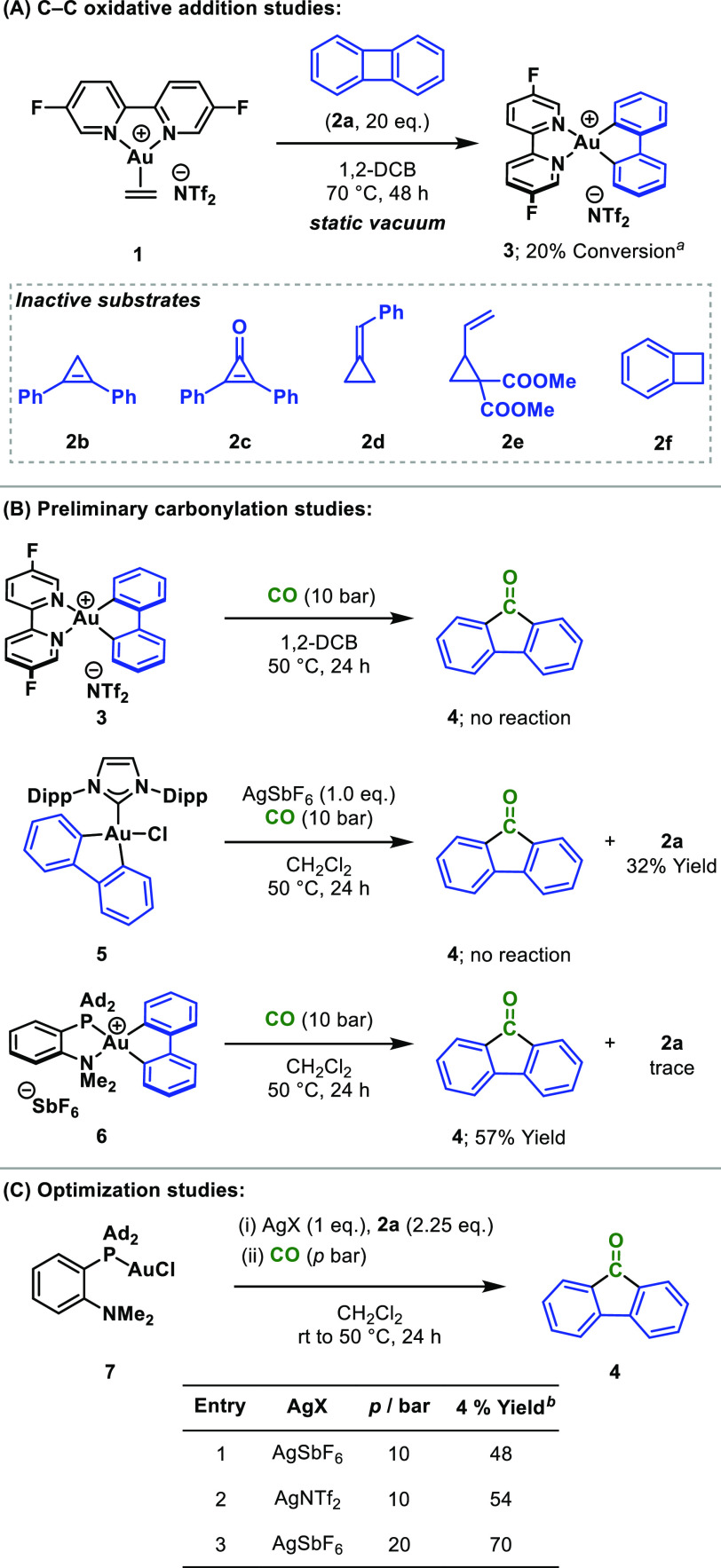
C–C Oxidative Addition and Carbonylation Studies Yield determined by ^19^F NMR spectroscopy using trifluorotoluene as an internal standard. Isolated yield. Dipp = 2,6-diisopropylphenyl.

Based on the above, complex **3** and known complexes **5** and **6** were good candidates for investigating CO migratory insertion processes. When exposed to 10 bar CO, **3** and **5** did not give target 9-fluorenone (**4**) (Scheme 2B). For **5**, reductive elimination of biphenylene occurred instead (32% yield), whereas **3** remained intact. We note both the similarity between and inactivity toward reaction with CO of **5** and the complexes employed by Hashmi et al. in their study on the reactivity of Au(III) carbonyl complexes.^19^ Pleasingly, the analogous experiment using the MeDalPhos ligated Au(III) complex, **6**, resulted in the formation of **4** in 57% yield,^25^ along with trace quantities (<5%) of **2a**. Only a 9% yield of **4** was obtained using a balloon pressure of CO (1 atm), which indicates the process is relatively challenging. 9-Fluorenone was also generated in one pot from MeDalPhosAuCl, by premixing this complex with CO (Scheme 2C). Using this procedure, **4** was generated in 48% yield at 10 bar of CO. Changing the Ag(I) salt to AgNTf_2_ increased the yield of **4** to 54%,^26^ whereas doubling the CO pressure to 20 bar provided **4** in 70% yield. The two-step telescoped procedure is required, as **4** was not obtained when a CO atmosphere was used at the outset. This suggests CO impedes C–C oxidative addition of **2a**, possibly because of the competing formation of inactive Au–CO species (see SI).^27^

To gain more insight into this Au-mediated carbonylation, the reaction was monitored by FTIR spectroscopy which showed three intense signals in the carbonyl region at 2152, 1714, and 1605 cm^–1^ (Scheme 3A,B). The signal at 2152 cm^–1^ is similar to that of free CO and is consistent with a complex possessing a weak Au(III)–CO interaction (e.g., **8**).^16,28^ The remaining two signals were not definitively assigned but can be attributed to either migratory insertion complex **9** or 9-fluorenone **4**. Further insight was obtained through the use of ^13^CO, which facilitated analysis of the mixture by ^13^C{^1^H} NMR spectroscopy. Importantly, the 9-fluorenone generated in this experiment showed full ^13^C incorporation (see SI). This experiment revealed three signals at δ_C_ = 193.6 (s), 188.5 (d), and 184.3 (br. s) ppm (Scheme 3C). The singlet at 193.6 ppm is the carbonyl group of **4**. Although not unequivocally assigned, we speculate the broad signal observed at 184.3 ppm is due to (reversible) binding of free CO to the Au(III) center (e.g., **8**), and the doublet at 188.5 ppm (^2^*J*_CP_ = 94 Hz) is consistent with acyl-Au complex **9**, where insertion of ^13^CO has occurred *trans* to the MeDalPhos P-unit.^29^ A complementary doublet of similar magnitude (^2^*J*_PC_ = 93 Hz) was observed in the ^31^P{^1^H} NMR spectrum (see SI). Complex **9** was also observed by mass spectrometry (ESI^+^, *m*/*z* 798.3120, calcd. 798.3139, [M–SbF_6_]^+^)(Scheme 3D). Tandem mass spectrometry showed that this ion fragments to a new ion whose mass was consistent with starting Au(I) fragment **10** (*m*/*z* 618.2551, calcd. 618.2564). This requires loss of 9-fluorenone **4** from **9**, thus equating to direct observation of reductive elimination from complex **9**. Mass spectrometry was also used to probe the apparent reversibility of oxidative addition of **2a** to **10** to give **6**. Exposure of a CH_2_Cl_2_ solution of the latter to biphenylene-*d*_8_ led to crossover, giving deuterated complex **11**, and confirming that **6** can undergo C–C reductive elimination of biphenylene (**2a**).

**Scheme 3:**
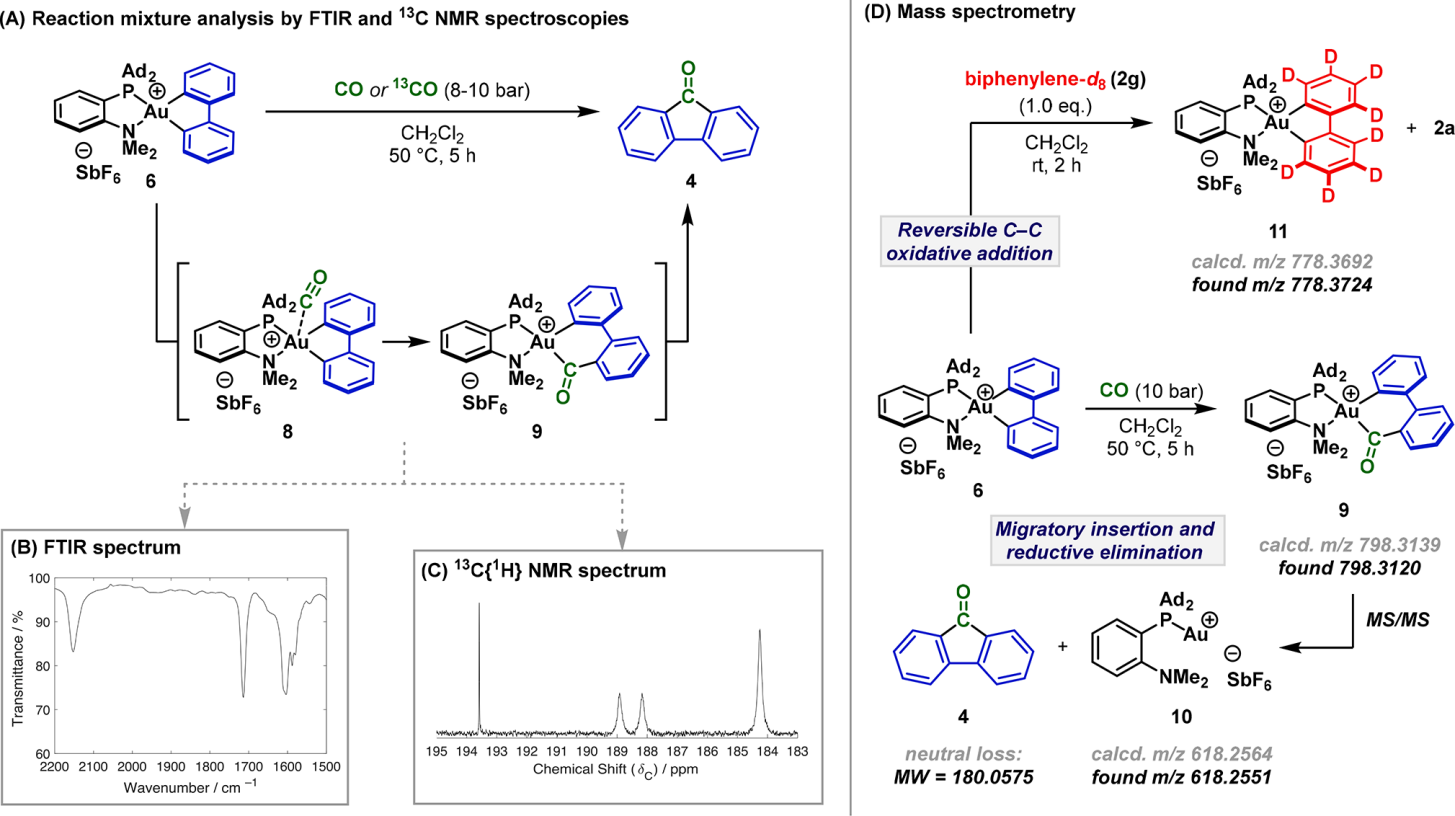
Mechanistic Studies of Migratory Insertion and Reductive Elimination

The data described so far underpin an outline carbonylation mechanism that could be probed computationally (Scheme 4). At the ωB97-XD level of theory with a CH_2_Cl_2_ solvent model, CO initially forms a long (3.3 Å) contact with the Au(III) center of **6** to give **Int1**, with a Au center marginally perturbed from its preferred square planar geometry (sum of angles at Au 359.5°). **Int1** rearranges with exchange of CO and NMe_2_, giving **Int2**, with a weakly bound NMe_2_ group (Au···N 3.0 Å; sum of angles at Au 359.5°).^30^ Exothermic migratory insertion (−35.6 kcal mol^–1^) then occurs with a barrier (from **Int1**) of 37.1 kcal mol^–1^ via **TS1** to give **Int3**.^31^ An analogous migratory insertion directly from **Int1** is *much higher* in energy (50.5 kcal mol^–1^ via **TS2**). Consistent with the ^13^C{^1^H} NMR data for complex **9**, the rearrangement of **Int1** to **Int2** forces CO to undergo migratory insertion into the Au–C bond that is *trans* to the MeDalPhos PAd_2_ unit. A similar change in coordination during a bisphosphine-ligated Pt-mediated carbonylation was reported in a computational study by Macgregor and Neave.^32^ The hemilability of the NMe_2_ group of MeDalPhos is clearly important for accessing the energetically viable CO migratory insertion pathway from **Int2**. Indeed, consistent with the results in Scheme 3B, the barrier to migratory insertion with NHC complex **5** is much higher (by 12 kcal mol^–1^) (see SI). Reductive elimination from **Int3** to give Au(I) species **10** and 9-fluorenone **4** is facile with a barrier of 7.8 kcal mol^–1^ via **TS3**, and the overall process (**6** to **10**) is exothermic (−31.0 kcal mol^–1^).

**Scheme 4:**
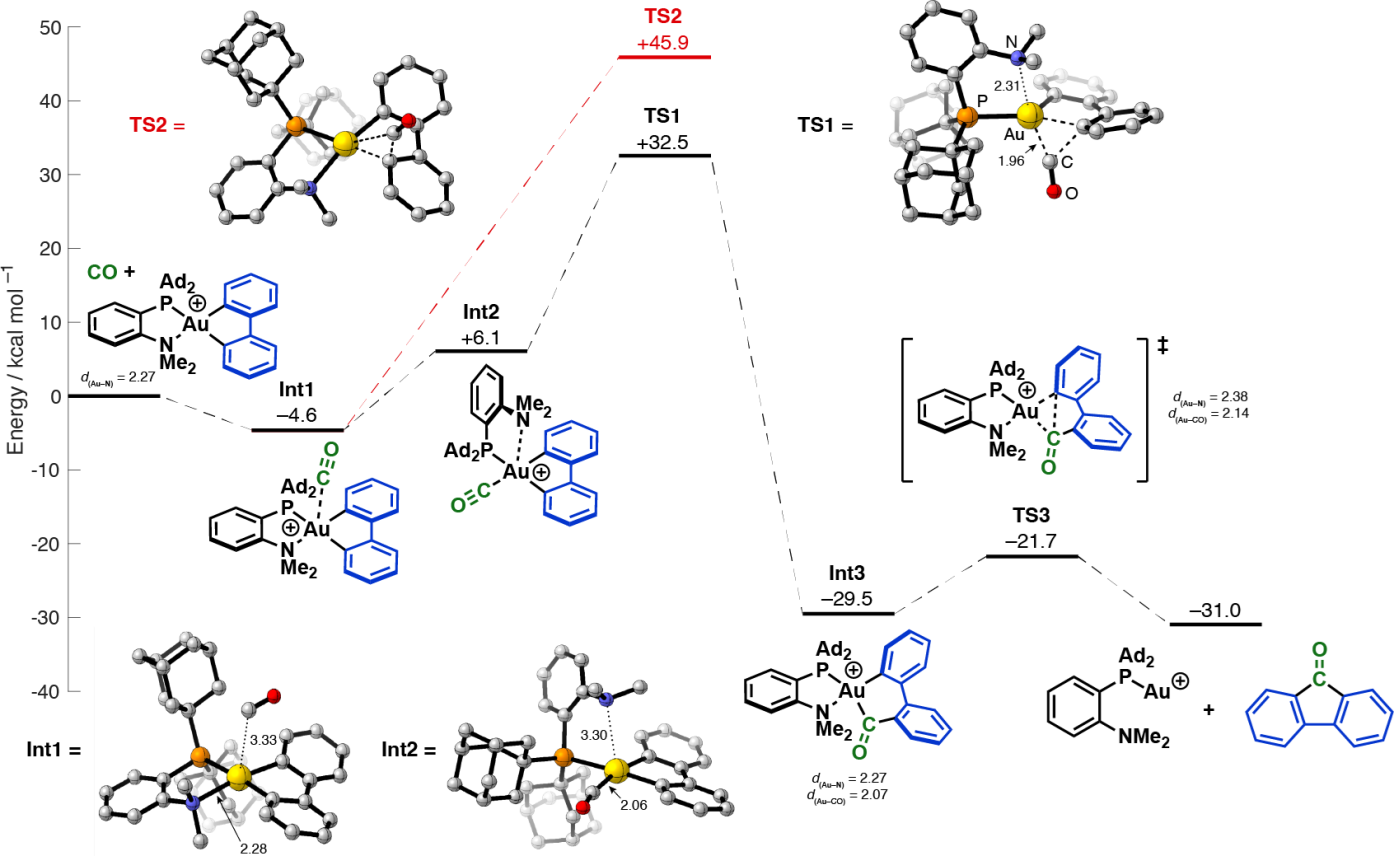
DFT Analysis of the Carbonylation Process Zero-point energies with solvation corrections are shown (see SI for details). Bond lengths are quoted in angstroms; for comparison, a Au–CO distance of 1.94 Å was calculated using the same level of theory for the *bona fide* Au(III)–CO complex, [(C^∧^N^∧^C)AuCO]^+^.^16,28a^

We next compared the propensity for retrocarbonylation at Au(III) versus Rh(III) centers; interestingly, decarbonylation was recently reported as the decomposition pathway for highly fragile Au(III) carboxylates possessing κ^3^-C,C,N-pincer ligands.^33^ The Au(III) acyl complex **12** was considered an ideal model system (*vide infra*), and this was accessed in 84% yield by reaction of benzocyclobutenone (**2h**) with the cation of MeDalPhosAuCl **7** (Scheme 5A). The process was selective for the C1–C2 bond of **2h**. When **12** was heated at 130 °C for 4 h, a 1:1 ratio of isomers **13a** and **13b** formed, where cleavage of the C1–C8 bond of **2h** had occurred. These results are in line with observations by Bourissou, Amgoune and co-workers using a carboranyl diphosphine Au(I) complex, which were rationalized in terms of the thermodynamic versus kinetic preference for C1–C8 and C1–C2 cleavage.^11^ To build upon this, we examined the mechanism of the rearrangement of **12** to **13a** and **13b**. There are two likely pathways: (1) retro-carbonylation and CO insertion (Pathway A) or (2) reductive elimination and reoxidative addition (Pathway B, Scheme 5B). The former is operative for analogous processes at Rh(III)-centers,^34^ and this offered a point of comparison. To probe the process further, a crossover experiment using Au(III) complex **12** and cyclobutenone **2i** was undertaken (Scheme 5B). A mixture of complexes **13a** and **14a** with their isomers (**13b** and **14b**) was observed by ^1^H NMR spectroscopy and mass spectrometry. This indicates that C–C reductive elimination and reoxidative addition are feasible (Pathway B), but it does not definitively rule out Pathway A.^35^ However, computational studies revealed that retrocarbonylation from acyl-Au(III) complex **9** has a *very* high barrier (Δ*E*^‡^ = 62 kcal mol^–1^), making the process significantly less favorable than analogous or similar Rh(III)-complexes (24–28 kcal mol^–1^).^34b^ The fact that Au–C carbonylation is essentially irreversible is of potential significance; carbonylations of, e.g., Rh–C bonds are often highly reversible, which means that high CO pressures or specific design features can be required to enforce access to the acyl-metal intermediate.^36^

**Scheme 5:**
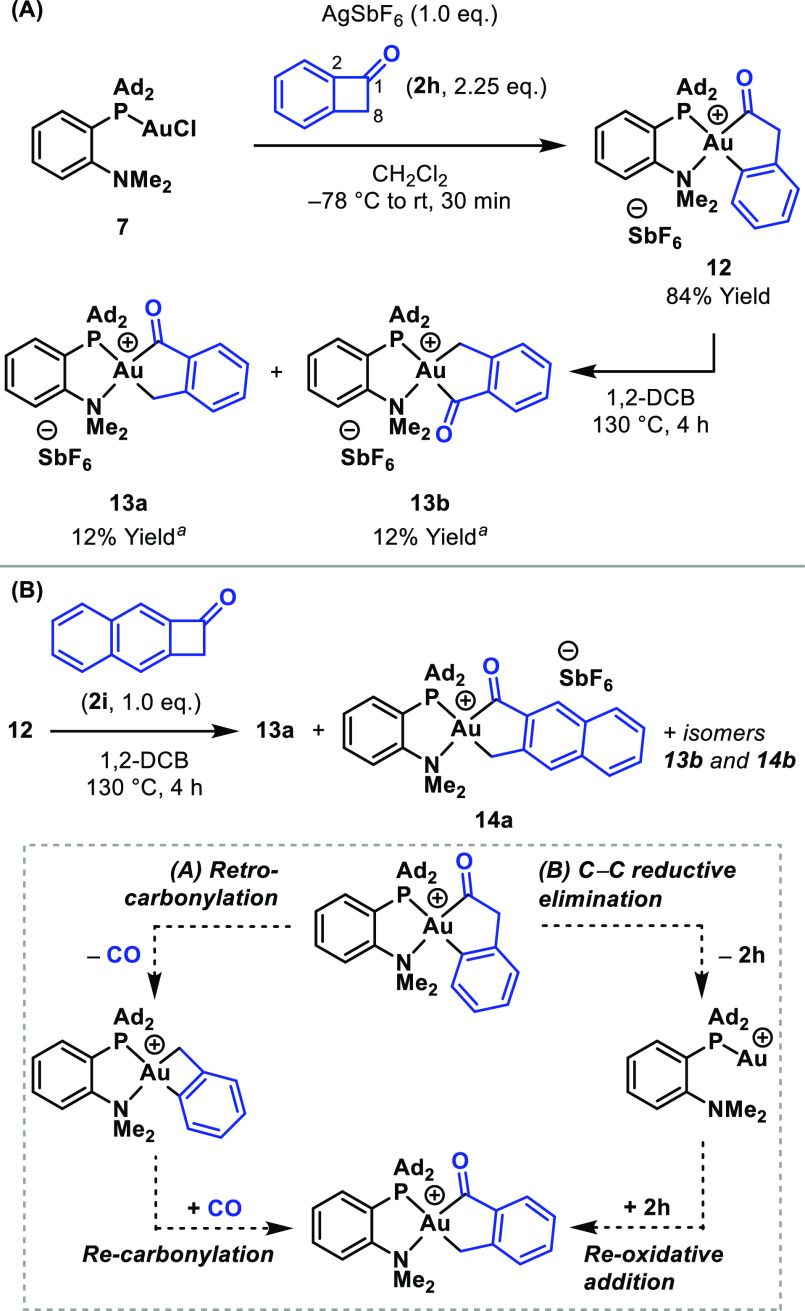
Reactivity of Benzocyclobutenone Complex **12** Yields determined by ^1^H NMR spectroscopy using a 1,3,5-trimethoxybenzene internal standard.

In conclusion, we have demonstrated migratory insertion of CO into a Au–C bond of a MeDalPhos-ligated aurafluorene complex. Experimental and computational investigations revealed the process occurs via a formal migratory insertion, with the hemilabile MeDalPhos being critical in allowing CO binding, and giving a significantly lower insertion barrier compared to other systems. The microscopic reverse, retro-carbonylation, was found to be a high energy process that is inaccessible under normal conditions. Compared to other transition metals, carbonylation at Au(III) centers affords migratory insertion complexes that are thermodynamically stable toward loss of CO.

## Supporting Information Available

Experimental details, characterization data, X-ray crystallography data, computational details. (PDF)

## References

[ref1] aSheldon R. A. E factors, green chemistry and catalysis: an odyssey. Chem. Commun. 2008, 3352–3365. 10.1039/b803584a. 18633490

[ref2] aTrost B. The atom economy - a search for synthetic efficiency. Science 1991, 254, 1471–1477. 10.1126/science.1962206. 1962206

[ref3] aWojcicki A. Insertion Reactions of Transition Metal-Carbon σ-Bonded Compounds I: Carbon Monoxide Insertion. In Advances in Organometallic Chemistry; Stone F. G. A., West R., Eds.; Academic Press: 1973; Vol. 11, pp 87–145.

[ref4] aAgbossou F.; Carpentier J.-F.; Mortreux A. Asymmetric Hydroformylation. Chem. Rev. 1995, 95, 2485–2506. 10.1021/cr00039a008.

[ref5] aJones J. H. The Cativa Process for the Manufacture of Acetic Acid. Platinum Metals Rev. 2000, 44, 94.

[ref6] aMurakami M.; Matsuda T. Metal-catalysed cleavage of carbon–carbon bonds. Chem. Commun. 2011, 47, 1100–1105. 10.1039/C0CC02566F. 20953480

[ref7] aCavell K. J. Recent fundamental studies on migratory insertion into metal-carbon bonds. Coord. Chem. Rev. 1996, 155, 209–243. 10.1016/S0010-8545(96)90182-4.

[ref8] aJoost M.; Amgoune A.; Bourissou D. Reactivity of Gold Complexes towards Elementary Organometallic Reactions. Angew. Chem., Int. Ed. 2015, 54, 15022–15045. 10.1002/anie.201506271. 26768342

[ref9] aJoost M.; Zeineddine A.; Estévez L.; Mallet-Ladeira S.; Miqueu K.; Amgoune A.; Bourissou D. Facile Oxidative Addition of Aryl Iodides to Gold(I) by Ligand Design: Bending Turns on Reactivity. J. Am. Chem. Soc. 2014, 136, 14654–14657. 10.1021/ja506978c. 25268830

[ref10] aPerthuisot C.; Edelbach B. L.; Zubris D. L.; Simhai N.; Iverson C. N.; Müller C.; Satoh T.; Jones W. D. Cleavage of the carbon–carbon bond in biphenylene using transition metals. J. Mol. Catal. A: Chem. 2002, 189, 157–168. 10.1016/S1381-1169(02)00203-0.

[ref11] Joost M.; Estévez L.; Miqueu K.; Amgoune A.; Bourissou D. Oxidative Addition of Carbon–Carbon Bonds to Gold. Angew. Chem., Int. Ed. 2015, 54, 5236–5240. 10.1002/anie.201500458. 25727203

[ref12] Wu C.-Y.; Horibe T.; Jacobsen C. B.; Toste F. D. Stable gold(III) catalysts by oxidative addition of a carbon–carbon bond. Nature 2015, 517, 449–454. 10.1038/nature14104. 25612049 PMC4304402

[ref13] Chu J.; Munz D.; Jazzar R.; Melaimi M.; Bertrand G. Synthesis of Hemilabile Cyclic (Alkyl)(amino)carbenes (CAACs) and Applications in Organometallic Chemistry. J. Am. Chem. Soc. 2016, 138, 7884–7887. 10.1021/jacs.6b05221. 27304485

[ref14] Font P.; Valdés H.; Guisado-Barrios G.; Ribas X. Hemilabile MIĈN ligands allow oxidant-free Au(I)/Au(III) arylation-lactonization of γ-alkenoic acids. Chem. Sci. 2022, 13, 9351–9360. 10.1039/D2SC01966C. 36093006 PMC9384699

[ref15] aRoşca D.-A.; Smith D. A.; Hughes D. L.; Bochmann M. A Thermally Stable Gold(III) Hydride: Synthesis, Reactivity, and Reductive Condensation as a Route to Gold(II) Complexes. Angew. Chem., Int. Ed. 2012, 51, 10643–10646. 10.1002/anie.201206468. 22997099

[ref16] Roşca D.-A.; Fernandez-Cestau J.; Morris J.; Wright J. A.; Bochmann M. Gold(III)-CO and gold(III)-CO_2_ complexes and their role in the water-gas shift reaction. Sci. Adv. 2015, 1, e1500761 10.1126/sciadv.1500761. 26601313 PMC4646827

[ref17] aRekhroukh F.; Brousses R.; Amgoune A.; Bourissou D. Cationic Gold(III) Alkyl Complexes: Generation, Trapping, and Insertion of Norbornene. Angew. Chem., Int. Ed. 2015, 54, 1266–1269. 10.1002/anie.201409604. 25353964

[ref18] aZhukhovitskiy A. V.; Kobylianskii I. J.; Wu C.-Y.; Toste F. D. Migratory Insertion of Carbenes into Au(III)–C Bonds. J. Am. Chem. Soc. 2018, 140, 466–474. 10.1021/jacs.7b11435. 29260868 PMC5765531

[ref19] Ahrens A.; Lustosa D. M.; Karger L. F. P.; Hoffmann M.; Rudolph M.; Dreuw A.; Hashmi A. S. K. Experimental and theoretical studies on gold(III) carbonyl complexes: reductive C,H- and C,C bond formation. Dalton Trans 2021, 50, 8752–8760. 10.1039/D1DT01315G. 34079966

[ref20] aLivendahl M.; Goehry C.; Maseras F.; Echavarren A. M. Rationale for the sluggish oxidative addition of aryl halides to Au(I). Chem. Commun. 2014, 50, 1533–1536. 10.1039/C3CC48914K. PMC4295554 24382586

[ref21] Our previous studies involving oxidative addition to C–I bonds (refs (9b), (9e), and (9f)) indicated that static vacuum is required to liberate ethylene to drive the oxidative addition equilibrium forward.

[ref22] Recently, Nevado et al. demonstrated C–C bond insertion with alkylidene cyclopropanes with a flanking pyridyl ring in the presence of Au(III) salts; see: González J. A.; Verdugo F.; Mascareñas J. L.; López F.; Nevado C. [C^N]-Alkenyl gold(III) complexes by proximal ring-opening of (2-pyridyl)alkylidenecyclopropanes: Mechanistic insights. Angew. Chem., Int. Ed. 2020, 59, 20049 10.1002/anie.202007371. 32671957

[ref23] aDaley R. A.; Morrenzin A. S.; Neufeldt S. R.; Topczewski J. J. Gold catalyzed decarboxylative cross-coupling of iodoarenes. J. Am. Chem. Soc. 2020, 142, 13210–13218. 10.1021/jacs.0c06244. 32634305

[ref24] aDinger M. B.; Henderson W. Synthesis and characterisation of the first auracyclobutane complex. J. Organomet. Chem. 1999, 577, 219 10.1016/S0022-328X(98)01043-2.

[ref25] Analogous reactions were attempted with an isoelectronic isocyanide; however, migratory insertion and reductive elimination were not observed. Instead, formation of a Au(I) isocyanide complex occurred (see SI for more details).

[ref26] This observation suggests there is little anion effect associated with migratory insertion, as oxidative addition of biphenylene in the presence of AgNTf_2_ is sluggish (see SI).

[ref27] aDias H. V. R.; Jin W. Coinage Metal Carbonyls and Isocyanides: Synthesis and Characterization of the Gold(I) Complexes [HB(3,5-(CF_3_)_2_Pz)_3_]AuCO and [HB(3,5-(CF_3_)_2_Pz)_3_]AuCNBut. Inorg. Chem. 1996, 35, 3687–3694. 10.1021/ic960186w.

[ref28] aGaggioli C. A.; Belpassi L.; Tarantelli F.; Belanzoni P. The gold(III)–CO bond: a missing piece in the gold carbonyl complex landscape. Chem. Commun. 2017, 53, 1603–1606. 10.1039/C6CC09879G. 28106186

[ref29] aBach I.; Goddard R.; Kopiske C.; Seevogel K.; Pörschke K.-R. Organometallics 1999, 18, 10 10.1021/om980705y.

[ref30] A Au–CO coordinate scan (with geometry optimizations at each stage) in the gas-phase was performed starting from **Int1**. This showed that upon decreasing the Au···CO distance coordination of the NMe_2_ group in the pseudoaxial position was favored (see SI).

[ref31] Although the migratory insertion barrier from **Int1** to **TS1** is high (at 298 K and 1 bar), we suspect that this barrier would be lowered under the reaction conditions (50 °C, 10–20 bar). This is consistent with experiment results where carbonylation is observed at high pressures and performs poorly at 1 atm.

[ref32] Macgregor S. A.; Neave G. W. Theoretical Study of the CO Migratory Insertion Reactions of Pt(Me)(OMe)(dppe) and Ni(Me)(OR)(bpy) (R = Me, O-*p*-C_6_H_4_CN): Comparison of Group 10 Metal–Alkyl, –Alkoxide, and –Aryloxide Bonds. Organometallics 2004, 23, 891–899. 10.1021/om030590k.

[ref33] Beucher H.; Merino E.; Genoux A.; Fox T.; Nevado C. κ^3^-(N^C^C)Gold(III) Carboxylates: Evidence for Decarbonylation Processes. Angew. Chem., Int. Ed. 2019, 58, 9064–9067. 10.1002/anie.201903098. 31059173

[ref34] aHuffman M. A.; Liebeskind L. S.; Pennington W. T. Reaction of cyclobutenones with low-valent metal reagents to form η^4^- and η^2^-vinylketene complexes. Reaction of η^4^-vinylketene complexes with alkynes to form phenols. Organometallics 1992, 11, 255–266. 10.1021/om00037a047.

[ref35] The isomerization shown in Scheme 5A was repeated under a pressure (1 bar) of ^13^CO, and no incorporation of ^13^CO was observed providing further evidence for Pathway B (see SI).

[ref36] aYagupsky G.; Brown C. K.; Wilkinson G. Further studies on hydridocarbonyltris(triphenylphosphine)rhodium(I); intermediate species in hydroformylation; rhodium and iridium analogues. J. Chem. Soc. A 1970, 1392–1401. 10.1039/j19700001392.

