# Supplementary material for: Migratory Insertion of CO into a Au–C Bond
Source: J Am Chem Soc. 2022 Oct 25;144(43):19719–25. doi: 10.1021/jacs.2c10432 (PMC9634805; doi:10.1021/jacs.2c10432)
Supplement: Supplementary file 1 — ja2c10432_si_001.pdf [file ja2c10432_si_001.pdf]

Migratory Insertion of CO into a Au–C Bond

Jamie A. Cadge,[a] *Paul J. Gates,*[a] *John F. Bower*[a,b,]* *and Christopher A. Russell*[a,]*

[a]School of Chemistry, University of Bristol, Cantock's Close, Bristol, U.K., BS8 1TS

[b]Department of Chemistry, University of Liverpool, Crown Street, Liverpool, U.K., L69 7ZD

Supporting Information

1. Materials and methods **2**

2. Experimental **4**

2.1 Substrate synthesis **4**

2.1.1 Biphenylene-d_6 (**2g**) synthesis 7

2.1.2 Naphthyl benzocyclobutenone **2i** synthesis 9

2.2 Syntheses of gold complexes **12**

2.2.1 Au(I) complexes 12

2.2.2 Au(III) biphenylene complexes 15

2.2.3 Au(III) benzocyclobutenone complexes 19

2.2.4 Unsuccessful oxidative additions 24

2.3 Carbonylation reactions **27**

2.3.1 From Au(III) complexes 27

2.3.2 From Au(I) complexes 30

2.3.3 Control experiment 30

2.4 Isocyanide reactivity **32**

3. Crystallographic information **36**

4. Mechanistic studies **39**

4.1 Biphenylene-d_8 cross-over experiment **40**

4.2 FTIR reaction mixture analysis **40**

4.3 ^{13}C-labelling NMR study **41**

4.4 Reaction mixture analysis by nanospray mass spectrometry **43**

4.5 CO incompatability with oxidative addition (^{31}P NMR monitoring) **44**

4.6. Benzocyclobutenone competition experiment **46**

5. Computational details **48**

5.1. General considerations **48**

5.2. Oxidative addition of cyclopropane-containing substrates **48**

5.2.1 Potential energy surfaces ... 48
5.2.2. Energies (au) and Cartesian coordinates (Å) for stationary points ... 50
5.3. Biphenylene vs benzocyclobutene oxidative addition ... 57
5.3.1. Potential energy surfaces ... 57
5.3.2. Energies (au) and Cartesian coordinates (Å) for stationary points ... 58
5.4. Carbonylation potential energy surfaces ... 65
5.4.1. MeDalPhos vs IPr ... 65
5.4.2. Energies (au) and Cartesian coordinates (Å) for stationary points ... 68
5.4.3. Comparison with a literature Au(III) CO complex ... 79
6. Selected spectra ... 81
7. References ... 88

1. Materials and methods

Materials

All starting materials were obtained from commercial suppliers and, except where otherwise stated, used as purchased. Anhydrous solvents (CH_2Cl_2, MeCN, hexane and THF) were dried using an Anhydrous Engineering Grubbs-type system (alumina)[1] and stored over 4 Å molecular sieves. TMEDA was distilled over CaH_2 and stored over 4 Å molecular sieves. CD_2Cl_2 was distilled over CaH_2 for NMR spectroscopy. $CDCl_3$ was used directly as purchased.

Methods

For all reactions performed, unless stated otherwise, inert conditions were employed using standard Schlenk line and/or glove box techniques under an atmosphere of dinitrogen or argon using oven-dried glassware. Reactions involving high pressure in autoclaves are described in Section 2. Room temperature (rt) typically fluctuated between 18 – 25 °C depending on the season and time of day. In instances where reactions were monitored, they were followed by NMR spectroscopy or analytical thin-layer chromatography (TLC). Merck TLC silica gel 60 F254 plates were used for TLC and were visualised using UV light and/or with a potassium permanganate solution and exposure to heat. Normal phase flash chromatography was carried out using 60 Å silica with solvent systems specified in Section 2.

Instrumentation

NMR spectroscopy: ^{1}H NMR spectra were measured at 300 MHz on a Jeol ECS 300, at 400 MHz on a Bruker Avance 400, Varian 400-MR, Jeol ECZ400 or Jeol ECS400 and at 500 MHz on a Bruker Avance III HD Cryo. Corresponding ^{13}C frequencies are 75.4 MHz, 101 MHz and 126 MHz. Corresponding ^{31}P frequencies are 121 MHz, 162 MHz and 202 MHz. Corresponding ^{19}F frequencies are 282 MHz, 376 MHz and 470 MHz. Corresponding ^{2}H frequencies are 46 MHz, 62 MHz and 77 MHz. NMR samples were analyzed as solutions with solvents specified below and, unless otherwise stated, at 298 K. ^{1}H and $^{13}C\{^{1}H\}$ NMR spectra were referenced to residual solvent peaks ($CDCl_3$ δ_H 7.26 and δ_C 77.2; CD_2Cl_2 δ_H 5.32 and δ_C 53.8). Chemical shifts (^{1}H and ^{13}C) are reported in parts per million (ppm) relative to tetramethylsilane standard. ^{31}P spectra were measured against 85% aq. solution of H_3PO_4 as an external standard. ^{19}F spectra were measured against CF_3Cl as an external standard. Other internal standards are specified below. Coupling constants (*J*) are reported to the nearest 0.1 Hz and were calculated using *MestReNova 11.0.* Coupling constants that did not match as a result of digitization are reported as rounded averages. Multiplicities are defined as followed: s singlet; d doublet; t triplet; q quartet; m multiplet and combinations thereof. Characterization of novel compounds was supported by 2D NMR experiments.

Mass spectrometry: Routine mass spectrometry was performed on a Bruker microTOF II spectrometer, a Thermo Scientific Orbitrap Elite mass spectrometer or a Thermo Scientific QExactive GC-MS by the University of Bristol mass spectrometry service. Positive ion nanospray mass spectrometry analyses (for analysis of gold complexes and reaction mixtures) were performed on a Synapt G2S mass spectrometer (Waters, Manchester, U.K.) equipped with a Nanomate Triversa chip-based nanospray system (Advion Biosciences, Norwich, U.K.). Samples were typcially dissolved in MeOH:water (50:50) to a concentration of 0.1 mg/mL immediately prior to analysis. The Nanomate was set to aspirate 5 μL of sample solution. Tandem mass spectrometry (MS/MS) was performed on isolated precursor ions (+/- 0.5 Da isolation window) at 22 eV collision energy using N_2 (g) as the collision gas.

FTIR spectroscopy: FTIR spectra were recored on a Perkin-Elmer Spectrum Two spectrometer with an attenuated total reflectance (ATR) (for air-stable solid samples) or a CaF_2 window solution cell (for air sensitive or reaction mixture samples) attachments.

Gas chromatography: GC-TIC/MS for qualitative reaction mixture analysis was performed on an Agilent 7820A GC equipped with a 5977E MSD.

2. Experimental

2.1 Substrate synthesis

Diphenylcyclopropenone (**2c**), benzocyclobutene (**2f**) and benzocyclobutenone (**2h**) were purchased from commercial sources and used as received.

Biphenylene (2a)

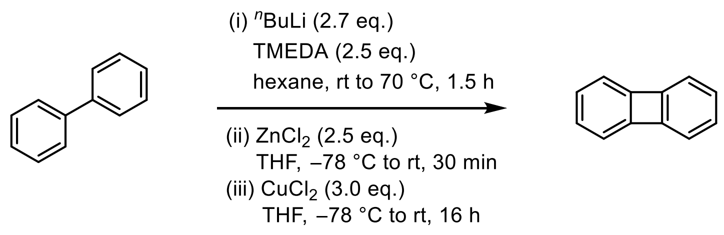

Following a modified literature procedure,[2] an oven-dried 3 L flask equipped with an overhead mechanical stirrer, dropping funnel and reflux condenser was purged with dinitrogen for 16 hours. Biphenyl (20.0 g, 122 mmol) followed by TMEDA (50 mL, 305 mmol) were then added. *n*-Butyllithium (130 mL of a 2.5 M solution in hexane) was added dropwise at room temperature over 30 minutes. Once addition was complete, the reaction mixture was heated at 70 °C for 1.5 hours. After cooling to room temperature, the solvent was evaporated *in vacuo* and the resultant residue was re-dissolved in THF (200 mL). The reaction mixture was cooled to –78 °C and $ZnCl_2$ (44 g, 305 mmol, dried under vacuum with heating for two hours) was added in portions. When addition was complete, the reaction mixture was warmed to room temperature and stirred for 30 minutes. The reaction mixture was then cooled again to –78 °C and $CuCl_2$ (52 g, 366 mmol, dried under vacuum with heating for two hours) was added. After warming to room temperature and stirring for 16 hours, the reaction mixture was poured on to aq. 1 M HCl (400 mL). Dichloromethane (400 mL) and brine (400 mL) were added sequentially and the organic portion was separated. The aqueous phase was washed with dichloromethane (3 × 400 mL). The organic extracts were combined, filtered through a pad of Celite and evaporated *in vacuo*. The resultant black residue was purified by sublimation (50 °C, 5×10^{-2} mbar) to give **2a** (2.24 g, 12%) as a colorless crystalline solid; **^{1}H NMR (400 MHz, $CDCl_3$)** δ

6.74 (dd, J = 4.9, 2.9 Hz, 4H, C_{Ar}-H), 6.63 (dd, J = 4.9, 2.9 Hz, 4H, C_{Ar}-H); **^{13}C{^{1}H} NMR (101 MHz, $CDCl_3$)** δ 151.5 (C_{Ar}), 128.4 (C_{Ar}), 117.5 (C_{Ar}). The spectroscopic properties of this compound were consistent with literature data.[2]

1,2-Diphenylcycloprop-1-ene (2b)

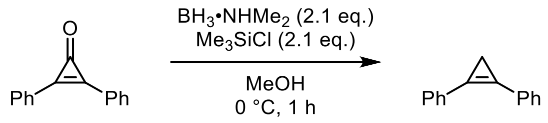

Following a modified literature procedure,[3] diphenylcyclopropenone (**2c**, 500 mg, 2.40 mmol) was dissolved in methanol (1.5 mL) and cooled to 0 °C. BH_3•$NHMe_2$ (300 mg, 5.10 mmol) was added as a solution in methanol (1.5 mL) followed by Me_3SiCl (0.63 mL, 5.00 mmol) as a solution in methanol (1.5 mL). After one hour, the reaction mixture was evaporated *in vacuo*. The resulting residue was dissolved in hexane (30 mL) and water (30 mL) was added. The organic portion was separated and the aqueous phase was washed with hexane (2 × 30 mL). The organic extracts were collected, dried ($MgSO_4$), filtered and evaporated *in vacuo* to give **2b** (367 mg, 80%) as a yellow waxy solid; **^{1}H NMR (400 MHz, $CDCl_3$)** δ 7.79 – 7.71 (m, 4H, C_{Ar}-H), 7.51 – 7.45 (m, 4H, C_{Ar}-H), 7.38 – 7.32 (m, 2H, C_{Ar}-H), 1.55 (s, 2H, CH_2). The spectroscopic properties of this compound were consistent with literature data.[4]

(Cyclopropylidenemethyl)benzene (2d)

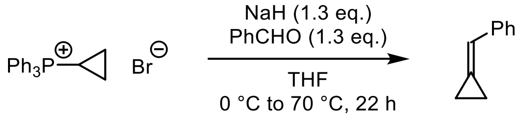

Following a modified literature procedure,[5] to a suspension of cyclopropyltriphenylphosphonium bromide (2.00 g, 5.20 mmol) and sodium hydride (160 mg, 6.80 mmol) in THF (10 mL) was added benzaldehyde (0.68 mL, 6.80 mmol) at 0 °C. The reaction mixture was then heated at 70 °C for 22 hours. Once cooled to room temperature, the reaction mixture was diluted with pentane (60 mL) and filtered though a short plug of silica. The filtrate was evaporated *in vacuo* and the crude material was purified by normal phase flash chromatography (pentane) to give **2d** (512 mg, 76%) as a colorless oil; **^{1}H NMR (400 MHz, $CDCl_3$)** δ 7.59 – 7.53 (m, 2H, C_{Ar}-H), 7.38 – 7.31 (m, 2H, C_{Ar}-H), 7.26 – 7.20 (m, 1H, C_{Ar}-H),

6.78 (p, J = 2.1 Hz, 1H, CH=C_{Ar}), 1.45 (ddd, J = 9.8, 5.7, 2.1 Hz, 2H, CH_2), 1.20 (ddd, J = 9.8, 5.7, 2.1 Hz, CH_2); **^{13}C{^{1}H} NMR (101 MHz, $CDCl_3$)** δ 138.4 (C_{Ar}), 128.6 (C_{Ar}), 126.8 (C_{Ar}), 124.4 (C_{Ar}), 118.4 (CH=C_{Ar}), 4.34 (CH_2=C), 0.64 (CH_2). The spectroscopic properties of this compound were consistent with literature data.[5]

Dimethyl 2-vinylcyclopropane-1,1-dicarboxylate (2e)

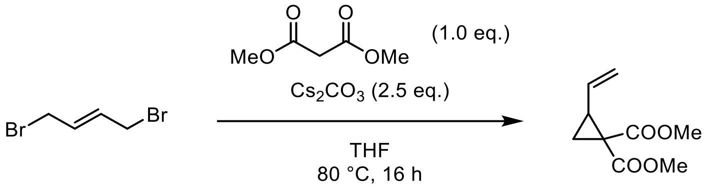

Following a modified literature procedure,[6] dibromobut-2-ene (5.00 g, 23.4 mmol) and dimethyl malonate (3.10 g, 23.4 mmol) were dissolved in THF (120 mL). Cesium carbonate (19.0 g, 58.4 mmol) was added and the reaction mixture was heated at 80 °C. After 16 hours, the reaction mixture was cooled to room temperature, filtered through a pad of Celite and washed with diethyl ether (100 mL). To the filtrate, sat. aq. $NaHCO_3$ (100 mL) and water (100 mL) were added. The organic portion was separated, dried (Na_2SO_4), filtered and evaporated *in vacuo*. The crude material was purified by normal phase flash chromatography (5% diethyl ether in hexane) to give **2e** (3.02 g, 70%) as a colorless solid; **^{1}H NMR (400 MHz, $CDCl_3$)** δ 5.46 – 5.33 (m, 1H, CH–CH=CH_2), 5.31 – 5.22 (m, 1H, CH–CH=CH_2), 5.11 (tt, J = 8.1, 2.2 Hz, 1H, CH–CH=CH_2), 3.75 – 3.66 (m, 6H, O–CH_3), 2.56 (h, J = 8.0 Hz, 1H, CH–CH=CH_2), 1.69 (dtt, J = 7.5, 4.8, 2.4 Hz, 1H, CH_2-a), 1.55 (tdd, J = 8.8, 4.8, 2.2 Hz, 1H, CH_2-b); **^{13}C{^{1}H} NMR (101 MHz, $CDCl_3$)** δ 170.1 (C=O), 167.9 (C=O), 133.0 (C–C=O), 118.8 (CH–CH=CH_2), 52.8 (O–CH_3), 52.7 (O–CH_3), 35.8 (C–C=O), 31.6 (CH_2), 20.7 (CH–CH=CH_2). The spectroscopic properties of this compound were consistent with literature data.[6]

2.1.1 *Biphenylene-d_6 (**2g**) synthesis*

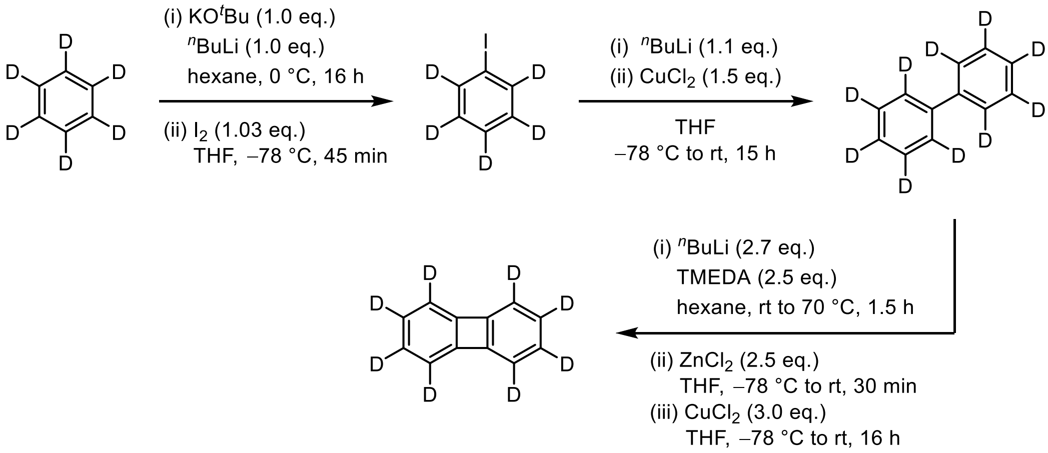

Iodobenzene-*d_5* (S1)

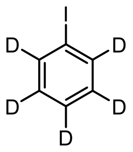

Following a modified literature procedure,[7] benzene-*d_6* (8.8 mL, 99 mmol) and KO^{t}Bu (11 g, 99 mmol) were suspended in hexane (40 mL) and cooled to –78 °C. *n*-Butyllithium (40 mL of a 2.5 M solution in hexane) was added dropwise over 30 minutes. Once addition was complete, the reaction mixture was warmed to 0 °C and stirred for 16 hours. The reaction mixture was cooled again to –78 °C and THF (20 mL) was added and stirred for 15 minutes. Iodine (26 g, 102 mmol) was added dropwise as a solution in THF (60 mL) over 30 minutes. Once addition was complete the reaction mixture was warmed to room temperature and the solvent removed by distillation. The resultant residue was dissolved in diethyl ether (60 mL), washed with sat. aq. $Na_2S_2O_3$ (3 × 60 mL) and brine (2 × 60 mL). The organic phase was evaporated *in vacuo* and purification was achieved by distillation (50 °C, 5 mbar) to give **S1** (4.4 g, 21%) as a light-red oil; **^{2}H NMR (61 MHz, $CHCl_3$)** δ 7.25 (s, 2D, C_{Ar}-*D*), 6.87 (s, 1D, C_{Ar}-*D*), 6.66 (s, 2D, C_{Ar}-*D*); **^{13}C NMR (101 MHz, $CHCl_3$)** δ 135.9 (t, *J* = 25.3 Hz, C_{Ar}), 129.6 (t, *J* = 24.8 Hz, C_{Ar}), 126.9 (t, *J* = 24.8 Hz, C_{Ar}). The spectroscopic properties of this compound were consistent with literature data.[7]

Biphenyl-d_{10} (S2)

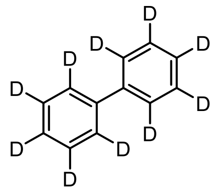

Iodobenzene-d_5 (**S1**, 3.00 g, 14.3 mmol) was dissolved in THF (60 mL) and cooled to –78 °C. *n*-Butyllithium (6.3 mL of a 2.5 M solution in hexane) was added dropwise over five minutes. The reaction mixture was then stirred for 30 minutes after which $CuCl_2$ (2.9 g, 21.5 mmol) was added. After stirring for a further five minutes, the reaction mixture was warmed to room temperature. After 15 hours, water (10 mL) and ethyl acetate (60 mL) were added sequentially. The organic portion was separated and the aqueous phase was washed with ethyl acetate (3 × 60 mL). The organic extracts were combined, dried ($MgSO_4$), filtered and evaporated *in vacuo*. The crude material was purified by normal phase flash chromatography (hexane) to give **S2** (567 mg, 24%) as a colorless solid; **^{2}H NMR (61 MHz, $CHCl_3$)** δ 7.15 (s, 4D, C_{Ar}-*D*), 6.99 (s, 4D, C_{Ar}-*D*), 6.90 (s, 2D, C_{Ar}-*D*); **^{13}C NMR (101 MHz, $CHCl_3$)** δ 140.9 (C_{Ar}), 128.2 (t, J = 24.4 Hz, C_{Ar}), 126.6 (t, J = 24.4 Hz, C_{Ar}).

Biphenylene-d_8 (2g)

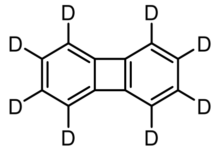

Following a similar procedure for the formation of biphenylene (**2a**) on a 1.91 mmol scale with biphenyl-d_{10} (**S2**), gave **2g** (27 mg, 8%) as a colorless solid; **^{2}H NMR (61 MHz, $CHCl_3$)** δ 6.28 (s, 4D, C_{Ar}-*D*), 6.17 (s, 4D, C_{Ar}-*D*); **^{13}C NMR (101 MHz, $CHCl_3$)** δ 151.4 (C_{Ar}), 127.9 (t, J = 24.6 Hz, C_{Ar}), 117.2 (t, J = 24.8 Hz, C_{Ar}); **HRMS (EI$^+$)** calcd. 160.1123 for $[C_{12}D_8]^+$ $[M]^+$, found 160.1119.

2.1.2 *Naphthyl benzocyclobutenone **2i** synthesis*

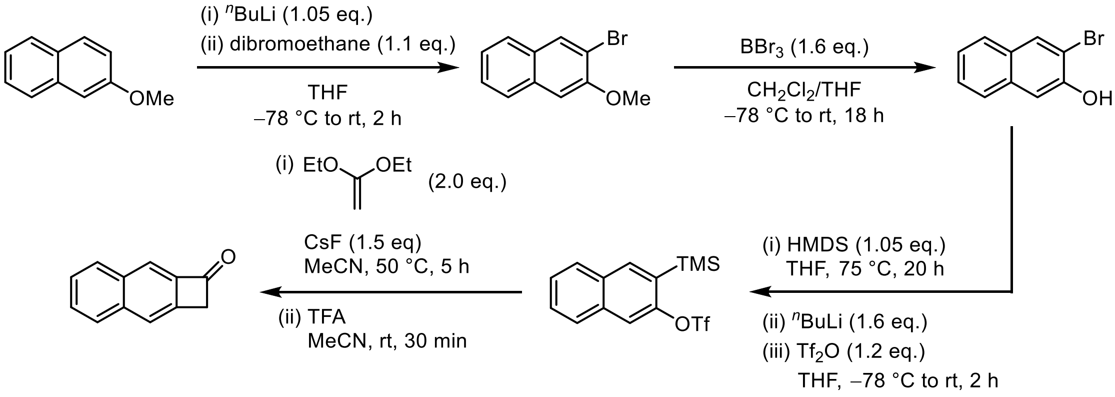

2-Bromo-3-methoxynaphthalene (S3)

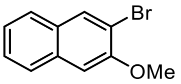

Following a modified literature procedure,[8] 2-methoxynaphthalene (7.90 g, 50.0 mmol) was dissolved in THF (60 mL) and cooled to –78 °C. *n*-Butyllithium (21.0 mL of a 2.5 M solution in hexane) was added dropwise over ten minutes and the reaction mixture was warmed to room temperature. After stirring for one hour, the reaction mixture was cooled again to –78 °C. Dibromoethane (4.74 mL, 55.0 mmol) was added, the reaction mixture was warmed to room temperature and stirred for one hour. The reaction mixture was then poured onto sat. aq. NH_4Cl (20 mL) and the organic layer was separated. The aqueous phase was washed with diethyl ether (3 × 30 mL) and the organic extracts were combined. The combined extracts were washed with brine (3 × 30 mL), dried ($MgSO_4$), filtered and evaporated *in vacuo*. The crude material was recrystallized from hexane to give **S3** (5.05 g, 43%) as a colorless solid; **^{1}H NMR (400 MHz, $CDCl_3$)** δ 8.06 (s, 1H, C_{Ar}-*H*), 7.68 – 7.73 (m, 2H, C_{Ar}-*H*), 7.46 (ddd, *J* = 8.2, 6.9, 1.3 Hz, 1H, C_{Ar}-*H*), 7.37 (ddd, *J* = 8.2, 6.9, 1.3 Hz, 1H, C_{Ar}-*H*), 7.16 (s, 1H, C_{Ar}-*H*), 4.00 (s, 3H, CH_3); **^{13}C{^{1}H} NMR (101 MHz, $CDCl_3$)** δ 153.7 (C_{Ar}), 133.7 (C_{Ar}), 132.4 (C_{Ar}), 129.6 (C_{Ar}), 126.9 (C_{Ar}), 126.8 (C_{Ar}), 126.7 (C_{Ar}), 124.6 (C_{Ar}), 106.8 (C_{Ar}), 56.4 (CH_3). The spectroscopic properties of this compound were consistent with literature data.[8]

3-Bromonaphthalen-2-ol (S4)

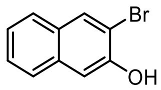

Following a modified literature procedure,[8] **S3** (3.00 g, 12.7 mmol) was dissolved in dichloromethane (20 mL) and cooled to –78 °C. Boron tribromide (20 mL of a 1.0 M solution in THF) was added dropwise over ten minutes and warmed to room temperature. After 18 hours, the reaction mixture was cooled to 0 °C and ice was slowly added to remove excess boron tribromide. The organic layer was separated and the aqueous phase was washed with dichloromethane (3 × 20 mL). The organic extracts were combined, washed with brine (3 × 20 mL), dried ($MgSO_4$), filtered and evaporated *in vacuo*. The crude material was recrystallized from hexane to give **S4** (1.63 g, 58%) as an off-white solid; **^{1}H NMR (400 MHz, $CDCl_3$)** δ 8.03 (s, 1H, C_{Ar}-*H*), 7.72 – 7.66 (m, 2H, C_{Ar}-*H*), 7.45 (ddd, *J* = 8.2, 6.9, 1.3 Hz, 1H, C_{Ar}-*H*), 7.40 (s, 1H, C_{Ar}-*H*), 7.35 (ddd, *J* = 8.2, 6.9, 1.3 Hz, 1H, C_{Ar}-*H*), 5.62 (s, 1H, O*H*); **^{13}C{^{1}H} NMR (101 MHz, $CDCl_3$)** δ 149.6 (C_{Ar}), 134.2 (C_{Ar}), 131.3 (C_{Ar}), 129.6 (C_{Ar}), 127.0 (C_{Ar}), 126.9 (C_{Ar}), 126.7 (C_{Ar}), 124.6 (C_{Ar}), 112.7 (C_{Ar}), 110.9 (C_{Ar}). The spectroscopic properties of this compound were consistent with literature data.[8]

3-(Trimethylsilyl)naphthalen-2-yl trifluoromethanesulfonate (S5)

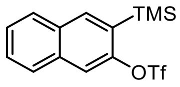

Following a modified literature procedure,[8] **S4** (1.00 g, 4.48 mmol) was dissolved in THF (15 mL) and HMDS (1.0 mL, 4.71 mmol) was added. The reaction mixture was heated at 75 °C for 20 hours, after which time was evaporated *in vacuo*. The resultant residue was dissolved in THF (30 mL) and cooled to –78 °C. *n*-Butyllithium (2.85 mL of a 2.5 M solution in hexane) was added dropwise over five minutes and the reaction mixture was stirred for one hour. Triflic anhydride (0.90 mL, 5.36 mmol) was added and the reaction mixture was stirred for a further hour. Before warming to room temperature, ice cold sat. aq. $NaHCO_3$ (10 mL) was added. The organics were separated and the aqueous phase was washed with diethyl ether (3 × 30 mL). The organic extracts were combined, washed with brine (20 mL), dried (Na_2SO_4), filtered and evaporated *in vacuo*. The crude material was purified by normal phase flash chromatography

(pentane) to give **S5** (1.16 g, 74%) as a pale yellow oil; **^{1}H NMR (400 MHz, CDCl$_3$)** δ 8.02 (s, 1H, C$_{Ar}$-*H*), 7.90 – 7.80 (m, 3H, C$_{Ar}$-*H*), 7.58 – 7.53 (m, 2H C$_{Ar}$-*H*), 0.45 (s, Si–C*H*$_3$); **^{13}C{^{1}H} NMR (101 MHz, CDCl$_3$)** δ 152.7 (*C*$_{Ar}$), 137.7 (*C*$_{Ar}$), 134.3 (*C*$_{Ar}$), 131.9 (*C*$_{Ar}$), 131.2 (*C*$_{Ar}$), 128.1 (*C*$_{Ar}$), 127.9 (*C*$_{Ar}$), 127.1 (*C*$_{Ar}$), 118.7 (q, *J* = 320 Hz, *C*F$_3$), 116.6 (*C*$_{Ar}$), -0.59 (Si–*C*H$_3$); **^{19}F NMR (377 MHz, CDCl$_3$)** δ -73.62 (C*F*$_3$). The spectroscopic properties of this compound were consistent with literature data.[8]

1,1-Diethoxyethene (S6)

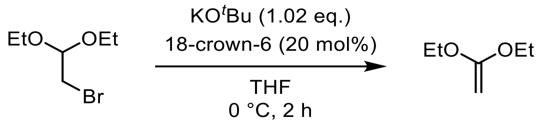

Following a modified literature procedure,[9] KO^{t}Bu (2.24 g, 20.0 mmol) and 18-crown-6 (1.04 g, 3.93 mmol) were dissolved in THF (15 mL) and cooled to 0 °C. 2-Bromo-1,1-dimethoxyethane (2.95 mL, 19.6 mmol) was added dropwise over two minutes and the reaction mixture was stirred for two hours. The solvent was distilled off and the remaining residue was purified by Kugelrohr distillation (65 °C, 1 atm) to give **S6** (572 mg, 25%) as a colorless oil; **^{1}H NMR (400 MHz, CDCl$_3$)** δ 3.53 (q, *J* = 7.1 Hz, 4H, O–C*H*$_2$), 1.45 (s, 2H, C=C*H*$_2$), 1.19 (t, *J* = 7.1 Hz, 6H, C*H*$_3$); **^{13}C{^{1}H} NMR (CDCl$_3$)** δ 114.3 (*C*=CH$_2$), 57.5 (C=*C*H$_2$), 20.3 (O–*C*H$_2$), 15.3 (*C*H$_3$). The spectroscopic properties of this compound were consistent with literature data.[9]

Cyclobuta[*b*]naphthalen-1(2*H*)-one (2i)

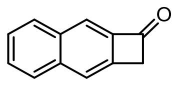

Following a modified literature procedure,[10] 1,1-diethoxyethene (**S6**, 82 mg, 0.706 mmol) and cesium fluoride (80 mg, 0.530 mmol) were suspended in acetonitrile (2 mL) in a screw-cap Schlenk tube. **S5** (123 mg, 0.353 mmol) was added, the tube was sealed and the reaction mixture was heated at 50 °C in a pre-heated oil bath. After five hours, the reaction mixture was cooled to room temperature and trifluoroacetic acid (54 μL, 0.706 mmol) was added. After 30 minutes, ethyl acetate (10 mL) and water (5 mL) were added. The organic layer was separated and the aqueous phase was washed with ethyl acetate (3 × 10 mL). The organic extracts were combined,

dried ($MgSO_4$), filtered and evaporated *in vacuo*. The crude material was purified by normal phase flash chromatography (10% ethyl acetate in hexane) to give **2i** (27 mg, 45%) as a colorless solid; **^{1}H NMR (500 MHz, $CDCl_3$)** δ 7.96 (d, *J* = 8.3 Hz, 1H, C_{Ar}-*H*), 7.91 – 7.84 (m, 3H, C_{Ar}-*H*), 7.61 (ddd, *J* = 8.3, 6.7, 1.3 Hz, 1H, C_{Ar}-*H*), 7.54 – 7.47 (m, 1H, C_{Ar}-*H*), 4.20 (d, *J* = 1.2 Hz, 2H, C*H*$_2$); **^{13}C{^{1}H} NMR (126 MHz, $CDCl_3$)** δ 190.8 (C_{Ar}), 146.7 (C_{Ar}), 143.7 (C_{Ar}), 138.0 (C_{Ar}), 133.8 (C_{Ar}), 131.1 (C_{Ar}), 128.8 (C_{Ar}), 128.5 (C_{Ar}), 126.3 (C_{Ar}), 121.8 (C_{Ar}), 120.4 (C_{Ar}), 53.2 (CH$_2$). The spectroscopic properties of this compound were consistent with literature data.[10]

2.2 *Syntheses of gold complexes*

2.2.1 *Au(I) complexes*

Au(I) complexes IPrAuCl and MeDalPhosAuCl (**7**) were purchased from commercial sources and were used as supplied.

[(κ^2-F_2-bipy)Au(η^2-C_2H_4)][NTf_2] (1)

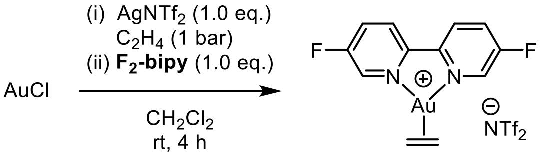

Following a modified literature procedure,[11] in a glovebox, a J. Young's tube was charged with gold(I) chloride (200 mg, 0.861 mmol) and silver(I) triflimide (334 mg, 0.861 mmol) in the absence of light. Outside the glovebox, the tube was placed under an atmosphere of ethylene (1 bar) and dichloromethane (40 mL) was added. After three hours stirring at room temperature, the reaction mixture was filtered through a pad of Celite into a flask containing **F_2-bipy**[12] (165 mg, 0.861 mmol) whilst maintaining darkness. After stirring for one hour, the solution was evaporated *in vacuo* to *approx.* 2 mL and filtered through a pad of Celite. The filtrate was layered with diethyl ether and stored for 24 hours at –18 °C to give **1** (360 mg, 60%) as colorless needles; **^{1}H NMR (400 MHz, CD_2Cl_2)** δ 8.71 (d, *J* = 2.7 Hz, 2H, C_{Ar}-*H*), 8.58 (dd, *J* = 9.1, 4.2 Hz, 2H, C_{Ar}-*H*), 8.11 – 8.03 (m, 2H, C_{Ar}-*H*), 3.93 (s, 4H, C*H*$_2$); **^{13}C{^{1}H} NMR (126 MHz, CD_2Cl_2)** δ 161.1 (d, *J* = 260 Hz, C_{Ar}), 147.8 (d, *J* = 3.1 Hz, C_{Ar}), 140.6 (d, *J* = 320 Hz, C_{Ar}), 129.0 (d, *J* = 18.6 Hz, C_{Ar}), 125.7 (d, *J* = 7.4 Hz, C_{Ar}), 119.8 (q, *J* = 320 Hz, anion CF$_3$), 63.8

(CH_2); **^{19}F NMR (377 MHz, CD_2Cl_2)** δ -77.53 (anion C*F*$_3$), -115.71 – -116.72 (C_{Ar}-*F*). The spectroscopic properties of this compound were consistent with literature data.[11]

***tert*-BuXPhosAuCl (S7)**

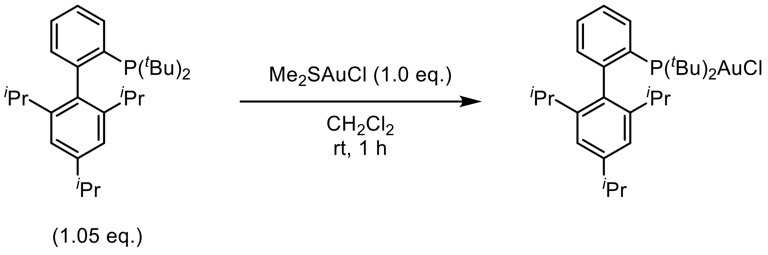

To a solution of Me_2SAuCl[13] (100 mg, 0.340 mmol) in dichloromethane (3.5 mL) *tert*-BuXPhos (156 mg, 0.365 mmol) was added as a solution in dichloromethane (3.5 mL). After one hour, the reaction mixture was filtered (using a Millipore Millex-HV 0.45 μm PVDF membrane syringe filter) and evaporated *in vacuo* to *approx.* 0.5 mL and hexane was added to precipitate a colorless solid. The mother liquor was removed and the solid was washed thrice with hexane to give **S7** (111 mg, 50%) as a colorless solid; **^{1}H NMR (500 MHz, CD_2Cl_2)** δ 7.90 (td, *J* = 7.8, 1.7 Hz, 1H, C_{Ar}-*H*), 7.56 – 7.47 (m, 2H, C_{Ar}-*H*). 7.29 (ddd, *J* = 7.2, 4.7, 1.8 Hz, 1H, C_{Ar}-*H*), 7.06 (s, 2H, C_{Ar}-*H*), 2.95 (hept, *J* = 6.9 Hz, 1H, C*H*–CH_3), 2.36 (hept, *J* = 6.7 Hz, 2H, C*H*–CH_3), 1.43 (s, 9H, P–C–C*H*$_3$), 1.40 (s, 9H, P–C–C*H*$_3$), 1.35 (d, *J* = 6.9 Hz, 6H, CH–C*H*$_3$), 1.28 (d, *J* = 6.8 Hz, 6H, CH–C*H*$_3$), 0.91 (d, *J* = 6.7 Hz, 6H, CH–C*H*$_3$); **^{13}C{^{1}H} NMR (126 MHz, CD_2Cl_2)** δ 150.6 (*C*$_{Ar}$), 148.8 (d, *J* = 14.1 Hz, *C*$_{Ar}$), 146.5 (*C*$_{Ar}$), 136.5 (d, *J* = 5.6 Hz, *C*$_{Ar}$), 135.5 (d, *J* = 8.1 Hz, *C*$_{Ar}$), 135.2 (d, *J* = 3.1 Hz, *C*$_{Ar}$), 130.8 (d, *J* = 2.4 Hz, *C*$_{Ar}$), 128.8 (d, *J* = 43.2 Hz, *C*$_{Ar}$), 127.0 (d, *J* = 6.9 Hz, *C*$_{Ar}$), 122.3 (*C*$_{Ar}$), 38.8 (d, *J* = 26.6 Hz, P–*C*–CH_3), 34.9 (*C*H–CH_3), 31.6 (d, *J* = 6.4 Hz, *C*H–CH_3), 31.4 (P–C–*C*H$_3$), 26.4 (CH–*C*H$_3$), 24.7 (CH–*C*H$_3$), 23.2 (CH–*C*H$_3$); **^{31}P{^{1}H} NMR (122 MHz, CD_2Cl_2)** δ 58.20; **HRMS (nanospray)** calcd. 621.2924 for $[C_{29}H_{45}AuClP]^+$ $[M]^+$, found 621.2939. The spectroscopic properties of this compound were consistent with literature data.[14]

[MeDalPhosAu(C≡N–^{t}Bu)][SbF_6] (S8)

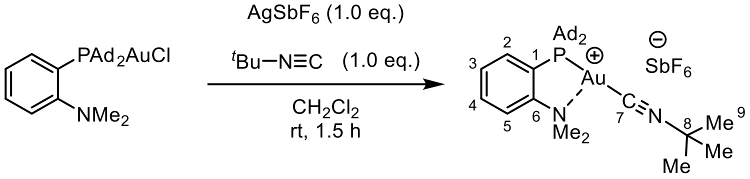

A mixture of MeDalPhosAuCl (50 mg, 0.0765 mmol) and *tert*-butyl isocyanide (9.0 μL, 0.0765 mmol) in dichloromethane (2 mL) was added dropwise to a solution of $AgSbF_6$ (26 mg, 0.0765 mmol) in dichloromethane (2 mL) at room temperature. After 1.5 hours, the reaction mixture was filtered (syringe filter) and concentrated to *approx.* 0.1 mL. Hexane was added which precipitated a colorless solid. The mother liquor was removed and the remaining solid was washed thrice with hexane and dried under vacuum to give **S8** (58 mg, 81%) as a colorless solid; **ν_{max} (ATR, neat) / cm^{-1}** 2909, 2854, 2786, 2239 (C≡N), 1587, 1474, 1450, 1374, 1302, 1143, 972, 930, 783, 746, 653; **^{1}H NMR (500 MHz, CD_2Cl_2)** δ 7.74 (ddd, *J* = 8.2, 6.9, 1.6 Hz, 1H, C5-*H*), 7.70 – 7.61 (m, 2H, C3-*H* and C4-*H*), 7.42 (ddt, *J* = 8.2, 6.9, 1.6 Hz, 1H, C2-*H*), 2.67 (s, 6H, C2-H_3), 2.24 – 2.15 (m, 6H, C_{Ad}-*H*), 2.10 – 1.97 (m, 11H, C_{Ad}-*H*), 1.73 (q, *J* = 13.6 Hz, 13H, C_{Ad}-*H*), 1.67 (s, 9H, C10-H_3); **^{13}C{^{1}H} NMR (126 MHz, CD_2Cl_2)** δ 159.5 (d, *J* = 7.1 Hz, *C*6), 136.0 (d, *J* = 1.7 Hz, *C*5), 134.2 (d, *J* = 1.8 Hz, *C*4), 126.9 (d, *J* = 7.0 Hz, *C*2), 126.4 (d, *J* = 4.5 Hz, *C*3), 122.9 (d, *J* = 49.4 Hz, *C*1), 78.1 (*C*8), 60.5 (*C*9), 48.8 (*C*7), 42.9 (d, *J* = 2.9 Hz, C_{Ad}), 42.3 (d, *J* = 22.2 Hz, C_{Ad}), 36.6 (d, *J* = 1.7 Hz, C_{Ad}), 30.1 (*C*10), 29.2 (d, *J* = 10.0 Hz); **^{31}P{^{1}H} NMR (162 MHz, CD_2Cl_2)** δ 54.78; **HRMS (nanospray)** calcd. 701.3299 for [M–SbF_6]$^+$ [$C_{33}H_{49}N_2PAu$]$^+$, found 701.3314. Crystals suitable for single-crystal X-ray diffraction were obtained by slow diffusion of a THF solution of **S8** with hexane at room temperature (X-ray data are given in Section 3).

2.2.2 *Au(III) biphenylene complexes*

[(F_2-bipy)aurafluorene][NTf_2] (3)

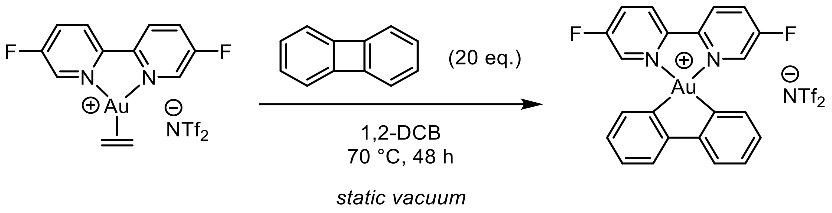

Following a modified literature procedure,[11] gold(I) ethylene complex **1** (19 mg, 0.0272 mmol) was dissolved in 1,2-dichlorobenzene and biphenylene (83 mg, 0.545 mmol) was added. The reaction mixture was subjected to ×3 freeze-pump-thaw cycles and heated at 70 °C for 48 hours. Analysis of the reaction mixture by ^{19}F NMR spectroscopy (Figure S1) gave two species at δ_F = –117.79 and δ_F = –114.47. The former is consistent with the presence of an Au(I) complex which can be attribiuted to the starting ethylene complex or a biphenylene π-bound species. The latter downfield signal is consistent with the formation of a Au(III) complex[11, 15] in approximately 20% conversion. Monitoring the reaction over the course of 48 hours, showed no change in the relative amounts of these two species suggesting an equilibrium. Analysis of the reaction mixture by nanospray mass spectrometry which gave *m*/*z* 541.0786 for $[M–NTf_2]^+$ (calcd. 541.0791). Additionally, consistent with the observations by ^{19}F NMR spectroscopy, the Au(I) ethylene complex was the most dominant ion observed. The poor conversion to complex **3** and its apparent fragility meant that no further analysis was performed.

Figure S1: ^{19}F NMR spectrum of the reaction mixture between **1** and biphenylene.

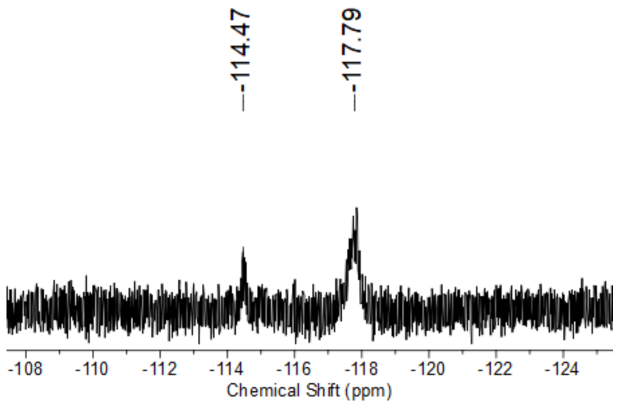

IPr(aurafluorene)Cl (5)

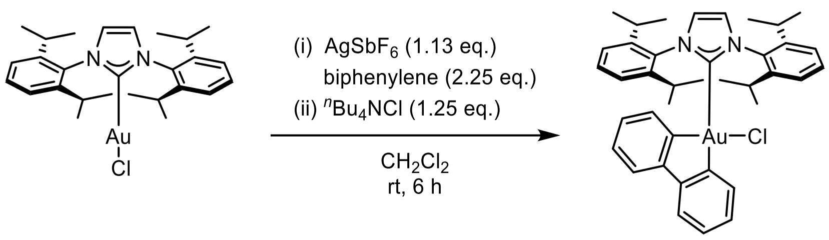

Following a modified literature procedure,[16] IPrAuCl (100 mg, 0.160 mmol) was dissolved in dichloromethane (8 mL) and $AgSbF_6$ (60 mg, 0.180 mmol) was added. After stirring for ten minutes at room temperature, the reaction mixture was filtered (using a Millipore Millex-HV 0.45 μm PVDF membrane syringe filter) and biphenylene (54 mg, 0.360 mmol) was added to the resultant filtrate. After three hours, $^{n}Bu_4NCl$ (54 mg, 0.200 mmol) was added. After a further three hours, the reaction mixture was evaporated *in vacuo* and the crude material was purified by normal phase flash chromatography (2:1 dichloromethane/hexane) to give **5** (68 mg, 55%) as a colorless solid; **^{1}H NMR (500 MHz, $CDCl_3$)** δ 8.04 (d, *J* = 7.7 Hz, 1H, C_{Ar}-*H*), 7.39 (t, *J* = 7.7 Hz, 2H, C_{Ar}-*H*), 7.35 (s, 2H, NHC[a]–C*H*), 7.31 – 7.12 (m, 6H, C_{Ar}-*H*), 7.02 (dt, *J* = 13.9, 7.3 Hz, 2H, C_{Ar}-*H*), 6.93 (t, *J* = 7.5 Hz, 1H, C_{Ar}-*H*), 6.88 (d, *J* = 7.5 Hz, 1H, C_{Ar}-*H*), 6.72 (t, *J* = 7.5 Hz, 1H, C_{Ar}-*H*), 3.17 (dp, *J* = 20.5, 6.7 Hz, 4H, C*H*–CH_3), 1.46 (d, *J* = 6.6 Hz, 6H, CH–CH_3), 1.18 (d, *J* = 6.8 Hz, 6H, CH–CH_3), 1.06 (d, *J* = 6.8 Hz, 6H, CH–CH_3), 0.88 (d, *J* = 6.7 Hz, 6H, CH–CH_3); **^{13}C{^{1}H} NMR (126 MHz, $CDCl_3$)** δ 188.4 (NHC–*C*), 159.2 (C_{Ar}), 154.5 (C_{Ar}), 153.0 (C_{Ar}), 152.6 (C_{Ar}), 147.3 (C_{Ar}), 144.9 (C_{Ar}), 134.3 (C_{Ar}), 133.6 (C_{Ar}), 133.2 (C_{Ar}), 130.9 (C_{Ar}), 127.0 (C_{Ar}), 126.8 (C_{Ar}), 126.7 (C_{Ar}), 125.6 (NHC–CH_2), 124.8 (C_{Ar}), 124.5 (C_{Ar}), 121.3 (C_{Ar}), 120.4 (C_{Ar}), 28.9 (*C*H–CH_3), 26.8 (CH–CH_3), 26.7 (CH–CH_3), 23.2 (CH–CH_3), 22.9 (CH–CH_3). The spectroscopic properties of this compound were consistent with literature data.[16]

[a] *NHC = N-heterocyclic carbene ligand fragment*

[(MeDalPhos)aurafluorene][SbF6] (6)

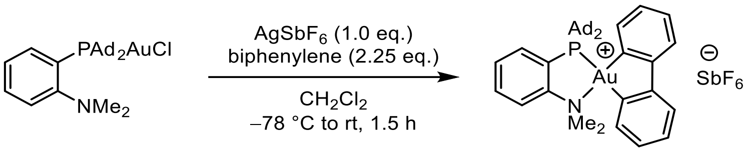

Following a modified literature procedure,[17] $AgSbF_6$ (52 mg, 0.152 mmol) was dissolved in dichloromethane (4 mL) and cooled to –78 °C. A solution of MeDalPhosAuCl (100 mg, 0.152 mmol) and biphenylene (52 mg, 0.342) in dichloromethane (4 mL) was added and the reaction mixture was stirred for ten minutes. After warming to room temperature, the reaction mixture was stirred for a further 1.5 hours. The reaction mixture was then filtered (using a Millipore Millex-HV 0.45 μm PVDF membrane syringe filter) and evaporated *in vacuo* to *approx.* 0.5 mL. Excess hexane was added to precipitate an orange solid. The mother liquor was removed and the solid was washed thrice with hexane. Drying under vacuum gave **6** (97 mg, 63%) as an orange solid; **^{1}H NMR (500 MHz, CD_2Cl_2)** δ 8.21 (dt, *J* = 8.1, 1.5 Hz, 1H, C_{Ar}-*H*), 8.02 (ddd, *J* = 8.1, 5.6, 1.4 Hz, 1H, C_{Ar}-*H*), 7.94 – 7.83 (m, 3H, C_{Ar}-*H*), 7.65 (ddt, *J* = 8.2, 6.8, 1.5 Hz, 1H, C_{Ar}-*H*), 7.57 (ddd, *J* = 7.6, 3.4, 1.7 Hz, 1H, C_{Ar}-*H*), 7.51 (dd, *J* = 7.7, 1.7 Hz, 1H, C_{Ar}-*H*), 7.40 – 7.26 (m, 3H, C_{Ar}-*H*), 7.01 (td, *J* = 7.7, 1.7 Hz, 1H, C_{Ar}-*H*), 3.73 (s, 6H, N–CH_3), 2.50 – 1.99 (m, 18H, C_{Ad}-*H*), 1.79 – 1.64 (m, 12H, C_{Ad}-*H*); **^{13}C{^{1}H} NMR (126 MHz, CD_2Cl_2)** δ 172.2 (d, *J* = 116.1 Hz, C_{Ar}), 163.3 (d, *J* = 11.6 Hz, C_{Ar}), 154.2 (d, *J* = 3.7 Hz, C_{Ar}), 153.5 (d, *J* = 1.7 Hz, C_{Ar}), 150.0 (d, *J* = 4.8 Hz, C_{Ar}), 142.1 (d, *J* = 6.7 Hz, C_{Ar}), 137.4 (C_{Ar}), 136.1 (d, *J* = 2.1 Hz, C_{Ar}), 131.2 (d, *J* = 4.7 Hz, C_{Ar}), 127.9 (d, *J* = 9.4 Hz, C_{Ar}), 126.5 (d, *J* = 2.6 Hz, C_{Ar}), 124.8 (d, *J* = 7.2 Hz, C_{Ar}), 123.8 (C_{Ar}), 122.9 (d, *J* = 5.5 Hz, C_{Ar}), 119.9 (d, *J* = 38.1 Hz, C_{Ar}), 55.6 (N–CH_3), 46.5 (d, *J* = 6.9 Hz, C_{Ad}), 42.4 (C_{Ad}), 36.0 (d, *J* = 1.8 Hz, C_{Ad}), 29.5 (d, *J* = 9.2 Hz, C_{Ad}); **^{31}P{^{1}H} NMR (162 MHz, CD_2Cl_2)** δ 77.29. The spectroscopic properties for this compound were consistent with literature data.[17]

[(MeDalPhos)aurafluorene][NTf_2] (S9)

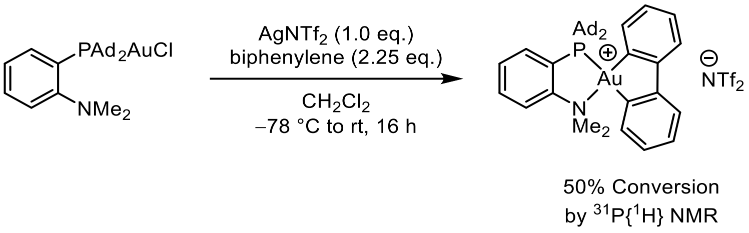

Following an equivalent procedure to the synthesis of gold(III) complex **3** using $AgNTf_2$ instead of $AgSbF_6$, the oxidative addition reaction was sluggish giving *approx.* 50% conversion to **S9** by $^{31}P\{^1H\}$ NMR spectroscopy after 16 hours (Figure S2). This in line with what has previously been observed by Bourissou and co-workers for the oxidative addition of aryl halides with MeDalPhosAuCl.[17]

Figure S2: $^{31}P\{^1H\}$ NMR spectrum of the reaction mixture for the attempted oxidative addition of biphenylene with MeDalPhosAuCl in the presence of $AgNTf_2$.

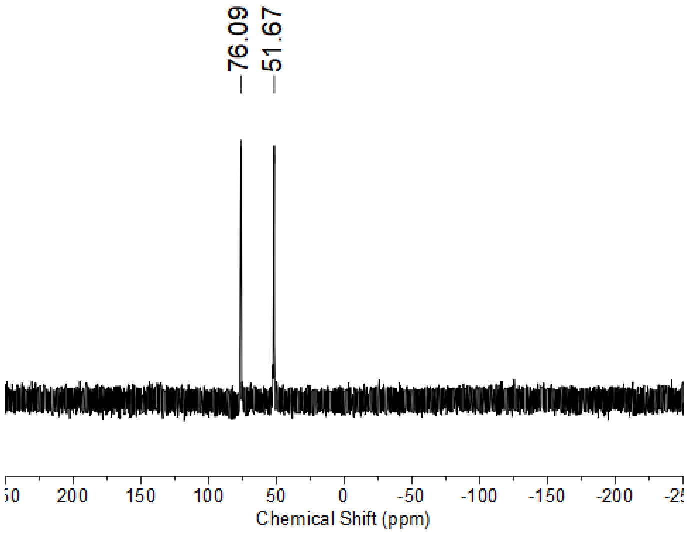

2.2.3 Au(III) benzocyclobutenone complexes

MeDalPhos benzocyclobutenone gold(III) acyl complex 12

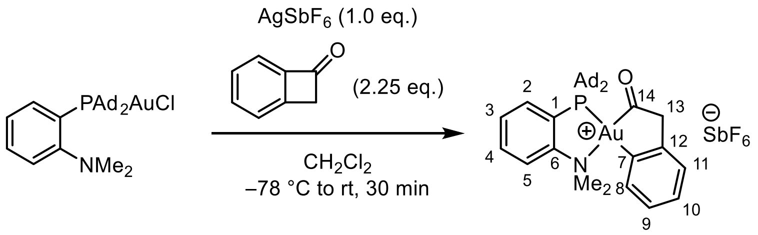

$AgSbF_6$ (52 mg, 0.153 mmol) was dissolved in dichloromethane (2 mL) and cooled to –78 °C and a solution in dichloromethane (2 mL) of MeDalPhosAuCl (100 mg, 0.153 mmol) and benzocyclobutenone (33 μL, 0.344 mmol) was added. After stirring for ten minutes, the reaction mixture was warmed to room temperature. After 30 minutes, the reaction mixture was filtered (using a Millipore Millex-HV 0.45 μm PVDF membrane syringe filter) and evaporated to *approx.* 0.5 mL. Hexane was added to precipitate a colorless solid and the mother liquor was removed. The remaining solid was washed thrice with hexane and dried under vacuum to give **12** (125 mg, 84%) as a colorless solid; **ν_{max} (ATR, neat) / cm^{-1}** 2910, 2851, 1763 (C=O), 1449, 1345, 1301, 1267, 1196, 1142, 1033, 1000, 972, 901, 781, 766, 751, 736, 654; **^{1}H NMR (500 MHz, CD_2Cl_2)** δ 7.88 – 7.85 (m, 3H, C2-*H*, C4-*H* and C5-*H*), 7.76 (app. t, *J* = 7.2 Hz, 1H, C8-*H*), 7.65 – 7.60 (m, 1H, C3-*H*), 7.56 – 7.49 (m, 2H, C9-*H* and C10-*H*), 7.46 (d, *J* = 7.3 Hz, 1H, C11-*H*), 4.51 (s, 2H, C13-H_2), 3.40 (s, 6H, N–CH_3), 2.39 – 2.01 (m, 18H, C_{Ad}-*H*), 1.85 – 1.69 (m, 12H, C_{Ad}-*H*); **^{13}C{^{1}H} NMR (126 MHz, CD_2Cl_2)** δ 188.5 (d, *J* = 4.6 Hz, *C*14), 163.4 (d, *J* = 96.3 Hz, *C*7), 161.6 (d, *J* = 11.4 Hz, *C*6), 140.1 (*C*12), 137.3 (*C*4), 136.0 (d, *J* = 2.0 Hz, *C*5), 131.1 (d, *J* = 4.4 Hz, *C*8), 129.8 (*C*3), 129.7 (*C*11), 128.1 (d, *J* = 7.6 Hz, *C*9 or *C*10), 125.1 (d, *J* = 6.7 Hz, *C*2), 123.9 (d, *J* = 6.9 Hz, *C*9 or *C*10), 121.3 (d, *J* = 39.7 Hz, *C*1), 67.9 (*C*13), 53.9 (N–*C*H_3), 44.4 (d, *J* = 14.4 Hz, C_{Ad}), 41.6 (C_{Ad}), 36.3 (d, *J* = 1.8 Hz, C_{Ad}), 29.2 (d, *J* = 9.6 Hz, C_{Ad}); **^{31}P{^{1}H} NMR (162 MHz, CD_2Cl_2)** δ 63.88; **HRMS (nanospray)** calcd. 736.2983 for $[M–SbF_6]^+$ $[C_{36}H_{46}NOPAu]^+$, found 736.2977. Crystals suitable for single-crystal X-ray diffraction were obtained by slow diffusion of a dichloromethane solution of **16** with hexane at room temperature (X-ray data are given in Section 3).

MeDalPhos naphthyl cyclobutenone gold(III) acyl complex (S10)

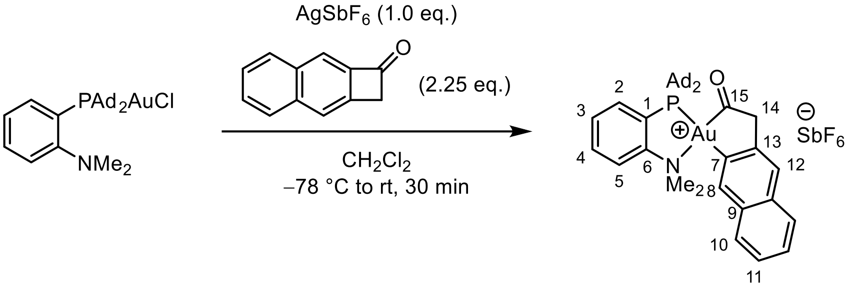

Following a similar procedure for the synthesis of **16** with **9i** on a 0.0185 mmol scale gave **S10** (8 mg, 42%) as a colorless solid; **ν_{max} (ATR, neat) / cm^{-1}** 2901, 2850, 1764 (C=O), 1451, 1344, 1301, 1267, 1126, 1046, 1011, 972, 902, 781, 765, 751, 655, 606; **^{1}H NMR (500 MHz, CD_2Cl_2)** δ 8.19 (d, *J* = 7.6 Hz, 1H, C5-*H*), 8.03 – 7.98 (m, 1H, C8-*H*), 7.94 (s, 1H, C13-*H*), 7.92 – 7.83 (m, 4H, C2-*H*, C4-*H* and C10-*H* or C11-*H*), 7.67 – 7.61 (m, 1H, C3-*H*), 7.61 – 7.54 (m, 2H, C10-*H* or C11-*H*), 4.67 (s, 2H, C14-H_2), 3.50 (s, 6H, N–CH_3), 2.38 – 2.28 (m, 6H, C_{Ad}-*H*), 2.17 – 2.08 (m, 12H, C_{Ad}-*H*), 1.84 – 1.72 (m, 12H, C_{Ad}-*H*); **^{13}C{^{1}H} NMR (126 MHz, CD_2Cl_2)** δ 188.6 (d, *J* = 4.3 Hz, *C*15), 162.63 (d, *J* = 96.7 Hz, *C*7), 161.6 (d, *J* = 11.3 Hz, *C*6), 137.3 (*C*10 or *C*11), 136.5 (*C*10 or *C*11), 136.1 (d, *J* = 1.9 Hz, *C*12), 134.0 (*C*9), 133.0 (d, *J* = 8.5 Hz, *C*13), 130.8 (d, *J* = 4.2 Hz, *C*5), 129.7 (d, *J* = 5.6 Hz, *C*3), 129.0 (*C*10′ or *C*11′), 127.9 (d, *J* = 8.0 Hz, *C*2), 127.0 (*C*10′ or *C*11′), 125.1 (d, *J* = 6.6 Hz, *C*4), 121.9 (d, *J* = 6.7 Hz, *C*8), 121.3 (d, *J* = 39.8 Hz, *C*1), 67.8 (*C*14), 54.1 (N–*C*H_3), 44.5 (d, *J* = 14.4 Hz, C_{Ad}), 41.6 (C_{Ad}), 36.3 (d, *J* = 1.8 Hz, C_{Ad}), 29.2 (d, *J* = 9.5 Hz, C_{Ad}); **^{31}P{^{1}H} NMR (122 MHz, CD_2Cl_2)** δ 64.65; **HRMS (nanospray)** calcd. 786.3139 for $[M–SbF_6]^+$ $[C_{40}H_{48}NOPAu]^+$, found 786.3141.

Benzocyclobutenone complex isomerisation

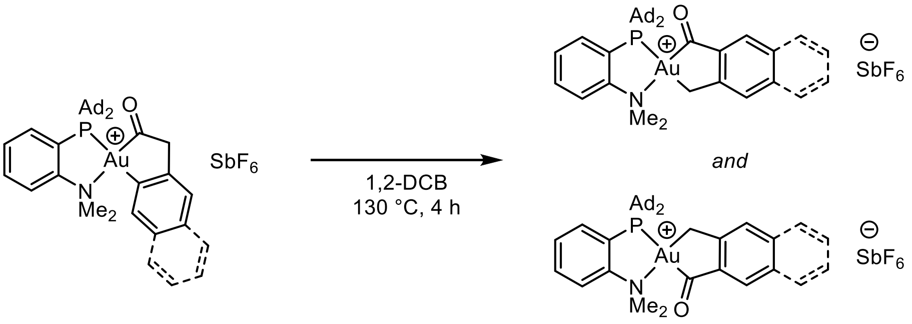

Benzocyclobutenone oxidative addition complexes **12** or **S10** were dissolved in 1,2-dichlorobenzene and stirred for four hours at 130 °C in a sealed tube. Once cooled to room temperature, the reaction mixture was filtered (using a Millipore Millex-HV 0.45 µm PVDF membrane syringe filter) and evaporated to dryness. The resultant residue containing **13a**/**13b** or **S11a**/**S11b** was redissolved in CD_2Cl_2 and subjected to analysis by NMR spectroscopy and mass spectrometry. *Due to the high temperatures required*[b] *for the isomerization to occur, full characterization was not possible due to significant degradation indicated by the formation of nanoparticles and gold mirroring of the reaction flask. However, NMR spectroscopic yields could be obtained (using a 1,3,5-trimethoxybenzene internal standard). Structural information diagnostic of the isomerization could also be obtained from the NMR spectra, namely from coupling between the phosphorus and the C=O and* CH_2 *of the bound benzocyclobutenone. These results are summarized below.*

[b] *High temperatures were required for isomerization, no reaction was observed at lower temperatures (50-90 °C).*

Figure S3: (A) ^{31}P-^{1}H NMR correlation experiment, (B) ^{1}H-^{13}C HSQC correlation experiment and (C) ^{1}H-^{13}C HMBC correlation of the isolated mixture containing gold(III) acyl complexes **13a** and **13b**.

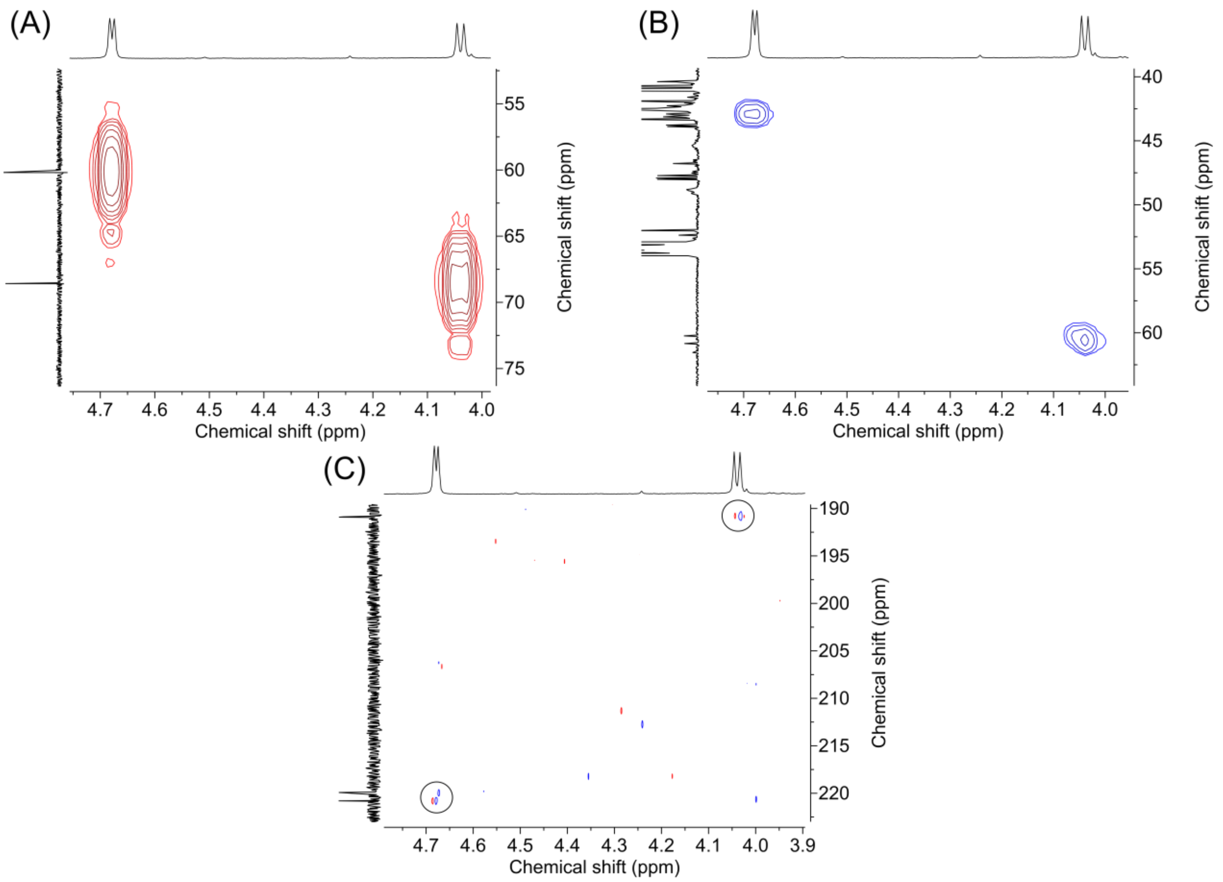

Figure S4: Summary of structural information gained from NMR correlation experiments (Figure S3) for (A) **13a** and (B) **13b**. Chemical shifts are given in ppm and coupling constants (J) in Hz.

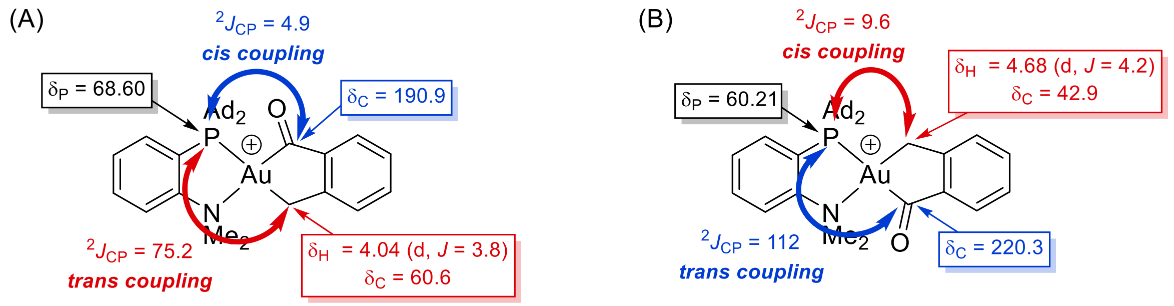

Figure S5: (A) ^{31}P-^{1}H NMR correlation experiment, (B) ^{1}H-^{13}C HSQC correlation experiment of the isolated reaction mixture containing gold(III) acyl complexes **S11a** and **S11b**. *In this case, the signal in the $^{13}C\{^{1}H\}$ NMR relating to the carbonyl carbon was not observed. However, the relative positions of the methylene carbons with respect to phosphorus could be determined.*

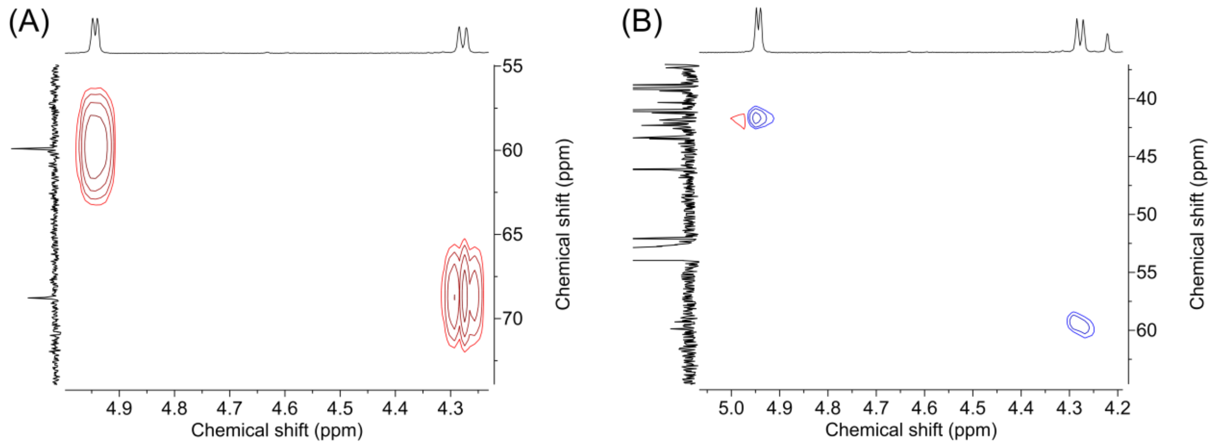

Figure S6: Summary of structural information gained from NMR correlation experiments (Figure S5) for gold(III) acyl complexes (A) **S11a** and (B) **S11b**. Chemical shifts are given in ppm and coupling constants (J) in Hz.

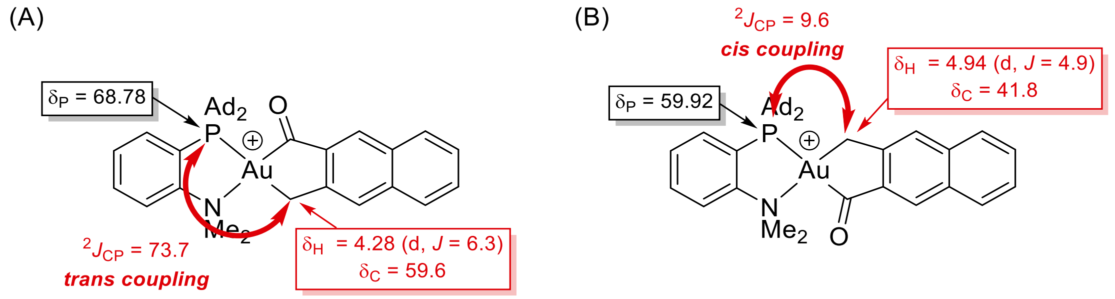

2.2.4 *Unsuccessful oxidative additions*

Attempted oxidative additions with other substrates containing strained C–C bonds:

All attempted oxidative additions outlined below were performed by following the procedures outlined above for their respective Au(I) complexes.

For all substrates with three-membered rings (**2b-e**) and benzocyclobutene (**2f**), no oxidative addition was observed by ^{19}F NMR spectroscopy when complex **1** was used. In all cases, the only species observed was related to the starting complex **1** (δ_F ~ –119 ppm). In cases where the substrate contained an accessible alkene moiety, another similar signal was observed, attributed to a π-bound species.

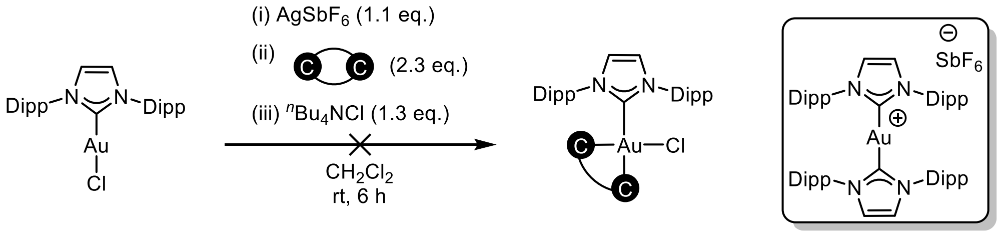

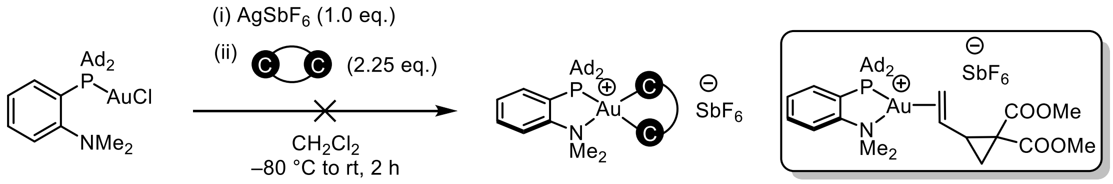

In the case of IPrAuCl, no reactivity with respect to oxidative addition was observed when a procedure similar to the synthesis of IPr biphenylene complex **5** was carried out. ^{1}H NMR spectroscopy was inconclusive, however, no desymmeterization of the 2,6-diisopropyl phenyl groups was observed which would be supportive of the formation of a Au(III) complex *cf.* **4**. Instead, in all cases, analysis of the reaction mixture by nanospray mass spectrometry showed the presence of a bis(NHC) Au(I) complex giving *m/z* 973.5413 for $[M–SbF_6]^+$ (calcd. 973.5422).

MeDalPhosAuCl gave the same reactivity profile as IPrAuCl, where decomposition was observed upon warming to room temperature from –78 °C. In all cases, new signals in the ^{31}P{^{1}H} NMR spectra were observed at δ_P ~ 59 ppm which were tentatively assigned to a

MeDalPhos Au(I) complex. This would either be a Au···SbF_6 adduct, after exchange of the chloride for SbF_6 or, in cases where substrates contained C=C bonds (*e.g.*, **2d** and **2e**) a π-bound complex could be formed. In the case of vinyl cyclopropane **2e**, this species was identified by nanospray mass spectrometery giving *m*/*z* 802.3319 for $[M–SbF_6]^+$ (calcd. 802.3299) which fragmented to a $MeDalPhosAu^+$ fragment giving *m*/*z* 618.2537 for $[M–SbF_6]^+$ (calcd. 618.2564). A series of similar alkene π-bound species with MeDalPhos have been reported by Bourissou and co-workers.[18] Similar inactivity was also observed with benzocyclobutene **2f**.

Attempted oxidative addition with biphenylene and *tert*-BuXPhosAuCl:

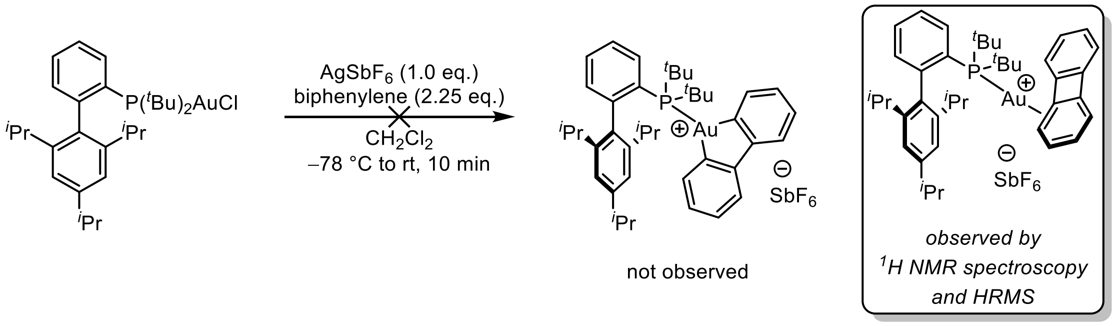

Following an idential procedure for the formation of MeDalPhosAuCl complex **6**, *tert*-BuXPhosAuCl (20 mg, 0.0304 mmol) was reacted with $AgSbF_6$ (10 mg, 0.0304 mmol) and biphenylene (10 mg, 0.0685 mmol). After warming to room temperature from –78 °C, immediate decomposition was observed indicated by the formation of Au nanoparticles. Analysis of the reaction mixture by $^{31}P\{^1H\}$ NMR spectroscopy showed no evidence of oxidative addition, instead a new signal at δ_P = –62.65 ppm was observed in line to those observed above with MeDalPhos Au(I) complexes (Figure S7). Additionally, analysis of the reaction mixture by ^1H NMR spectroscopy (after evaporating the reaction mixture to dryness *in vacuo* and re-dissolving in CD_2Cl_2) showed broading of the signals related to biphenylene (Figure S8). Similar broading in ^1H NMR spectra was observed by Toste *et al.* in relation to the oxidative addition of biphenylene with IPrAuCl.[16] It was shown that these signals arise from biphenylene forming a π-complex with the Au metal center prior to oxidative addition. Moreover, nanospray mass spectrometric analysis of the reaction mixture gave rise to a weak signal relating to this biphenylene π-complex giving *m*/*z* 773.3572 for $[M–SbF_6]^+$ (calcd. 773.3572). Although this mass would also be consistent with the oxidative addition of biphenylene, the NMR spectroscopic data is *not* consistent with this.

Figure S7: $^{31}P\{^1H\}$ NMR spectrum of the reaction mixture of the attempted oxidative addition of biphenylene with *tert*-BuXPhosAuCl.

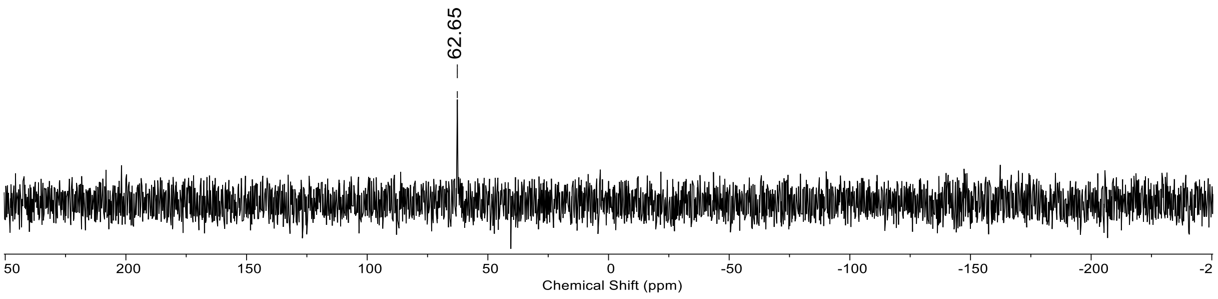

Figure S8: ^1H NMR spectrum of isolated material from the attempted oxidative addition of biphenylene with *tert*-BuXPhosAuCl. Signals highlighted with arrows are speculated to arise from a π-bound biphenylene.

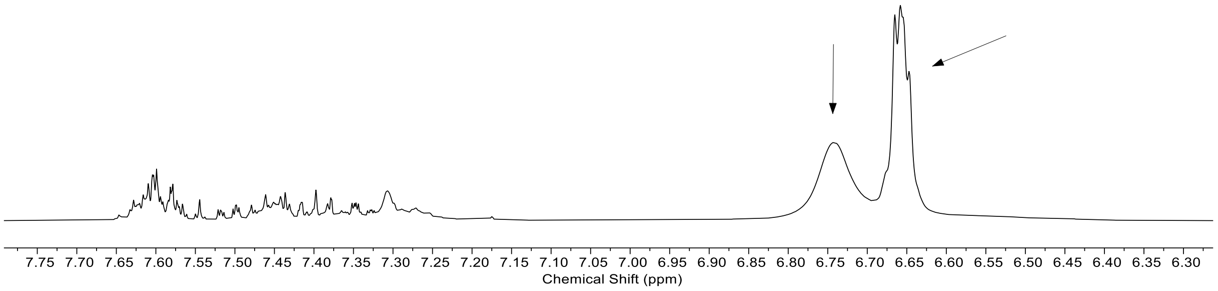

Oxidative addition with 4-fluoroiodobenzene (20 eq.) was also attempted which gave rise to decomposition and no changes observed in the ^{19}F NMR spectra of the reaction mixture. Identical results were achieved when 1,4-dioxane was used as the solvent.

2.3 Carbonylation reactions

2.3.1 From Au(III) complexes

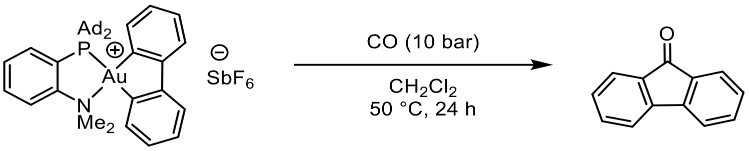

[(MeDalPhos)aurafluorene][SbF_6] (**6**) (40 mg, 0.0397 mmol) was added to a 35 mL stainless steel autoclave with a glass insert and dissolved in dichloromethane (4 mL). The autoclave was then sealed and pressurised to 10 bar of carbon monoxide. The autoclave was placed in an aluminum heating block and heated at 50 °C for 24 hours. After cooling to room temperature by removing the autoclave from the heating block and placing on ice, the autoclave was vented. An aliquot (typically <0.1 mL) of the reaction mixture was taken for analysis by GC-MS (Figure S9) and the remainder was evaporated *in vacuo*. The crude material was purified by normal phase flash chromatography (0 to 10% ethyl acetate in hexane) to give 9-fluorenone (**4**, 4.1 mg, 57%) as a pale yellow solid; **^{1}H NMR (500 MHz, CD_2Cl_2)** δ 7.63 (dt, *J* = 7.4, 1.0 Hz, 2H, C_{Ar}-*H*), 7.57 (dt, *J* = 7.4, 1.0 Hz, 2H, C_{Ar}-*H*), 7.52 (td, *J* = 7.4, 1.2 Hz, 2H, C_{Ar}-*H*), 7.32 (td, *J* = 7.4, 1.2 Hz, C_{Ar}-*H*); **^{13}C{^{1}H} NMR (126 MHz, CD_2Cl_2)** δ 194.0 (*C*=O), 144.8 (C_{Ar}), 135.1 (C_{Ar}), 134.5 (C_{Ar}), 129.5 (C_{Ar}), 124.4 (C_{Ar}), 120.8 (C_{Ar}); **GC-MS (EI$^+$)** calcd. *m/z* 180.1 for [M^+], found *m/z* 180.1 (100%), 152.1 (38.7%), 151.1 (21.6%), 76.0 (14.1%), 181.0 (13.5%), 150.0 (13.5%). Data consistent with values reported in the literature.[19]

For the procedure with 1 atm carbon monoxide, the carbonylation was performed in a Schlenk tube which was purged with carbon monoxide prior to the reaction. The Au(III) complex **6** (40 mg, 0.0397 mmol) was dissolved in dichloromethane (4 mL) and the tube was sealed with a virgin septum. A balloon containing carbon monoxide (purged thrice with carbon monoxide) was then placed through the septum. The reaction mixture was added to a pre-heated oil bath at 50 °C for 24 hours. The isolation procedure was identical to that above.

Figure S9: GC-MS analysis of the carbonylation reaction mixture with MeDalPhos biphenylene complex **6**. (A) TIC with signals at 7.82 min and 8.80 min corresponding to biphenylene and 9-fluorenone respectively as indicated by the mass spectra in (B) and (C), respectively.

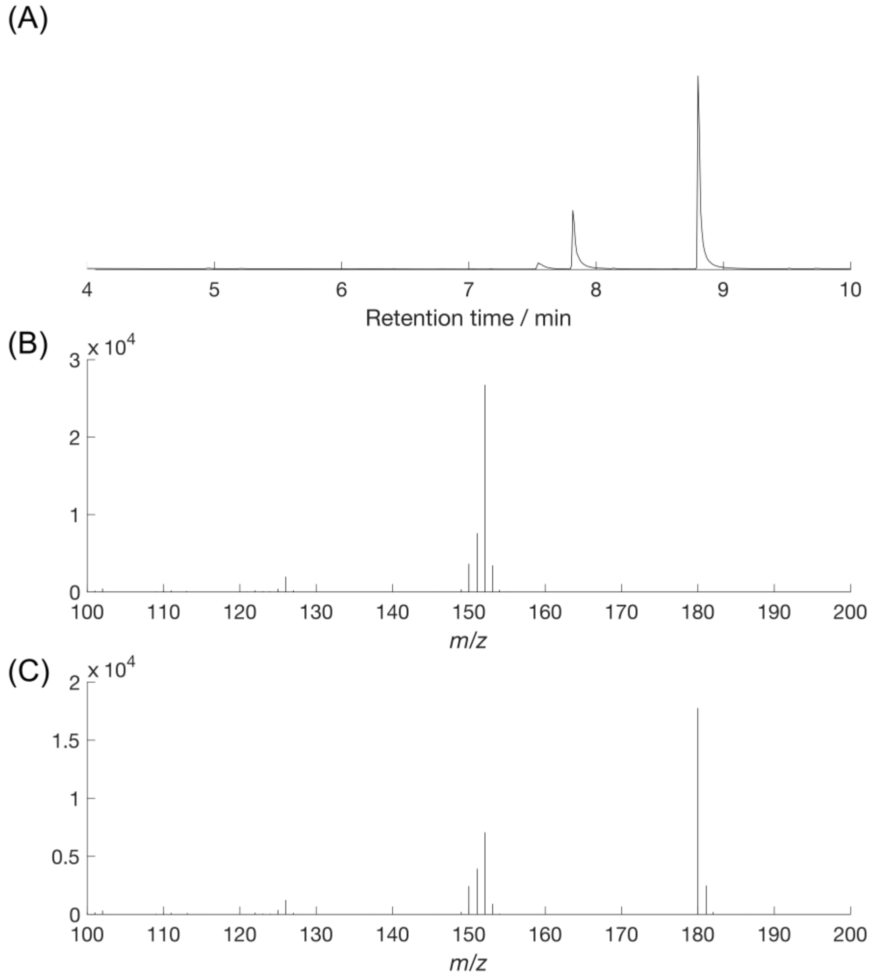

The above procedure was repeated for both complexes **1** and **5** which gave trace and 33% yield of biphenylene respectively. The GC-MS TIC trace does not show the presence of 9-fluorenone, with the major species as biphenylene (Figure S10).

Figure S10: GC-MS of reaction mixture of the attempted carbonylation with IPr aurafluorene complex **5**. (A) TIC and (B) mass spectrum of signal at 7.83 min corresponding to biphenylene.

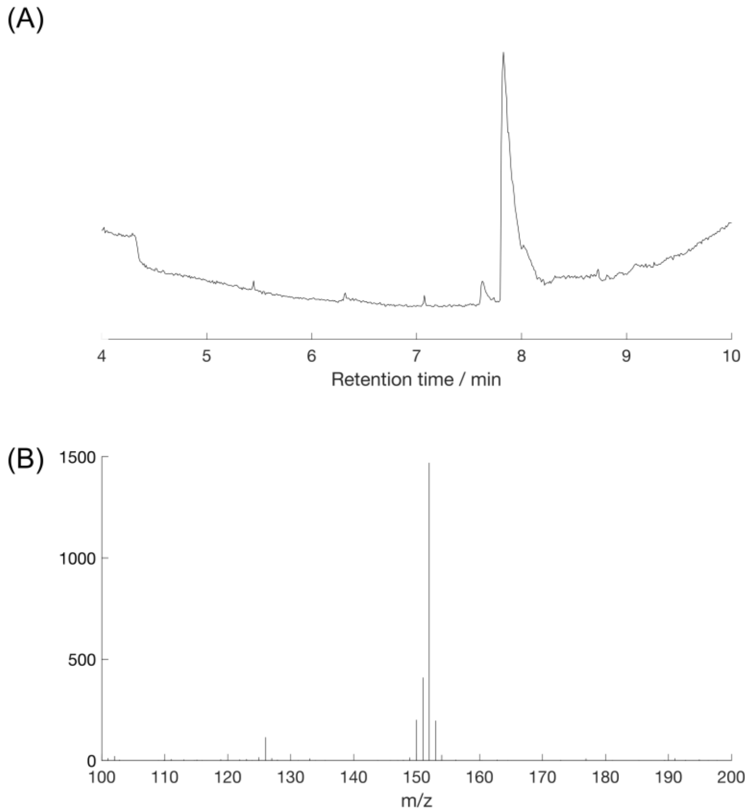

2.3.2 From Au(I) complexes

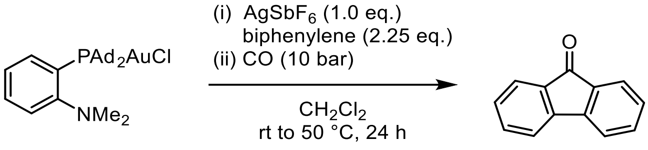

MeDalPhosAuCl (40 mg, 0.0612 mmol) and biphenylene (21 mg, 0.138 mmol) were added to a 35 mL stainless steel autoclave with a glass insert and dissolved in dichloromethane (6 mL). $AgSbF_6$ (21 mg, 0.0612 mmol) was then added, the autoclave sealed and the reaction mixture was stirred at room temperature for two hours. The autoclave was then pressurized with 10 bar of carbon monoxide, placed in an alumnium heating block and heated at 50 °C for 24 hours. After cooling to room temperature by removing the autoclave from the heating block and placing on ice, the autoclave was vented. The reaction mixture was evaporated *in vacuo* and the crude material was purified by normal phase flash chromatography (0 to 10% ethyl acetate in hexane) to give 9-fluorenone (**4**, 5.3 mg, 48%) as a pale yellow solid. Data consistent with 9-fluorenone isolated in the above example directly from the Au(III) complex **3**.

This procedure was repeated for the three-membered ring substrates (**2b**-**e**) and benzocyclobutene (**2f**) and none of the desired carbonylation products were detected by GC-MS analysis of the reaction mixtures.

2.3.3 Control experiment

To ensure that the carbonylation reaction to form 9-fluorenone was mediated by gold, and not by a direct reaction of carbon monoxide and biphenylene, a control experiment was carried out.

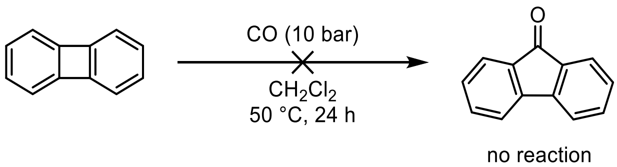

Biphenylene (14 mg) was added to a 35 mL stainless steel autoclave with a glass insert and stirrer bar and dissolved in dichloromethane (1.8 mL). The autoclave was sealed, pressurized to 10 bar carbon monoxide, placed in an aluminum heating block and heated at 50 °C. After 24 hours, the autoclave was cooled to room temperature by placing on ice and vented. The reaction

mixture was diluted with dichloromethane and subjected to analysis by GC-MS (Figure S11). The resultant TIC trace shows only the presence of biphenylene and the absence of 9-fluorenone.

Figure S11: (A) TIC trace of the blank reaction with biphenylene and carbon monoxide only and (B) mass spectrum of signal at 7.82 min related to biphenylene.

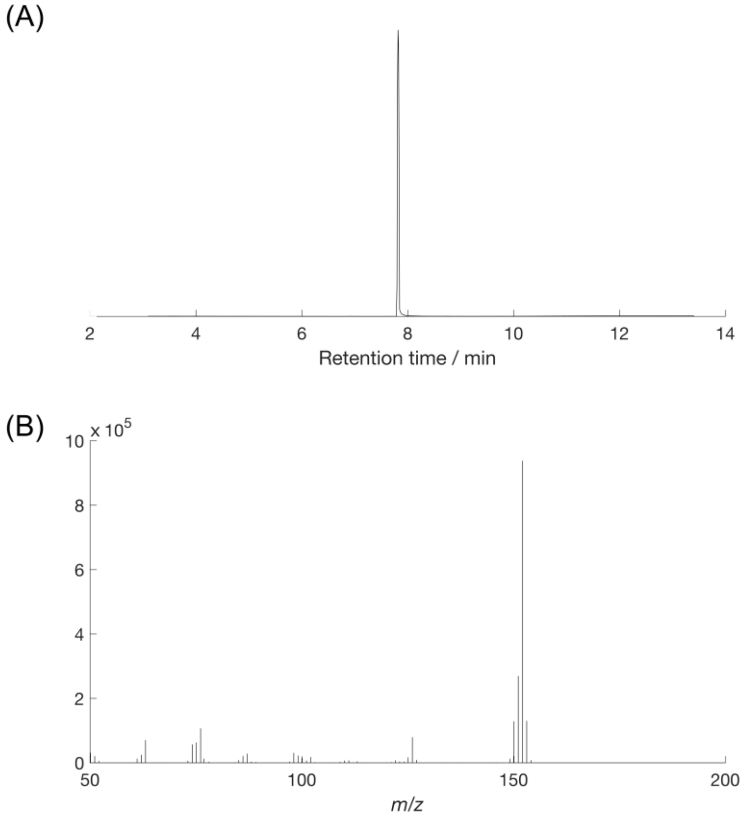

2.4 *Isocyanide reactivity*

To provide insight into the reactivity of isocyanides, commerically available *tert*-butyl isocyanide was exposed to MeDalPhos aurafluorene complex **6** in 1.1 and 10 equivalents.

Reaction with 1.1 eq. *tert*-butyl isocyanide:

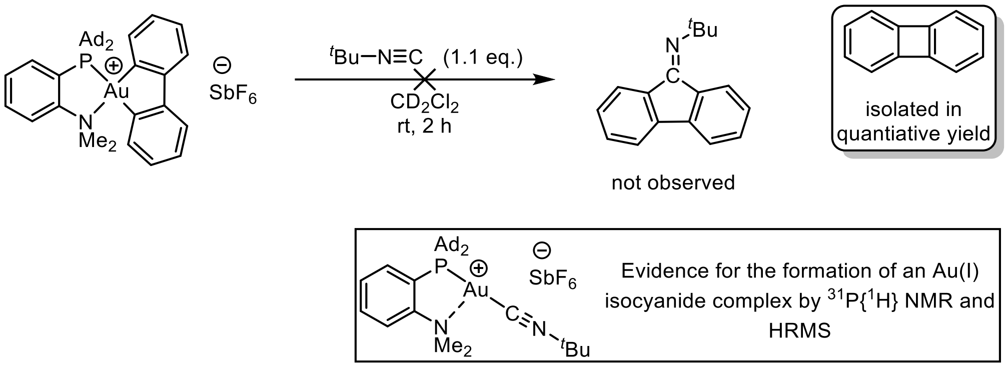

MeDalPhos aurafluorene complex **6** (15 mg, 0.0149 mmol) was dissolved in CD_2Cl_2 (1.5 mL) and *tert*-butyl isocyanide (1.7 µL, 0.0149 mmol) was added. Upon addition, the bright orange solution of **6** became colorless immediately. After stirring for two hours at room temperature, the reaction mixture was analysed by ^{1}H NMR and $^{31}P\{^{1}H\}$ NMR spectroscopies (Figures S12 and S13, respectively) and GC-MS. The reaction mixture was evaporated *in vacuo* and purification by normal phase flash chromatography (0 to 10% ethyl acetate in hexane) to give biphenylene **2a** in quantitative yield. Analysis of the reaction mixture by GC-MS did not show the desired imine product **S12**. Running the equivalent reaction at 50 °C also did not give rise to the expected product. Toste *et al.* when attempting a similar migratory insertion and reductive elimination with IPr aurafluorene complex **5** only gave trace yield of **S12**.[20]

Figure S12: $^{31}P\{^1H\}$ NMR spectrum of the reaction mixture for the attempted migratory insetion of *tert*-butyl isocyanide. Signals at δ_P = 77.25 and δ_P = 54.72 correspond to the Au(III) biphenylene complex **3** and Au(I) isocyanide complex **S8**, respectively.

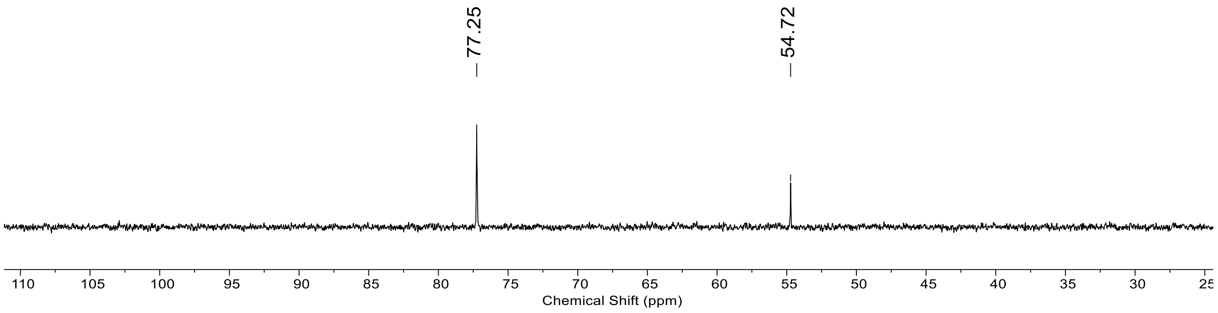

Figure S13: ^1H NMR spectrum of the reaction mixture for the attempted migratory insertion of *tert*-butyl isocyanide. Signals highlighted with arrows in the aromatic region are related to free biphenylene.

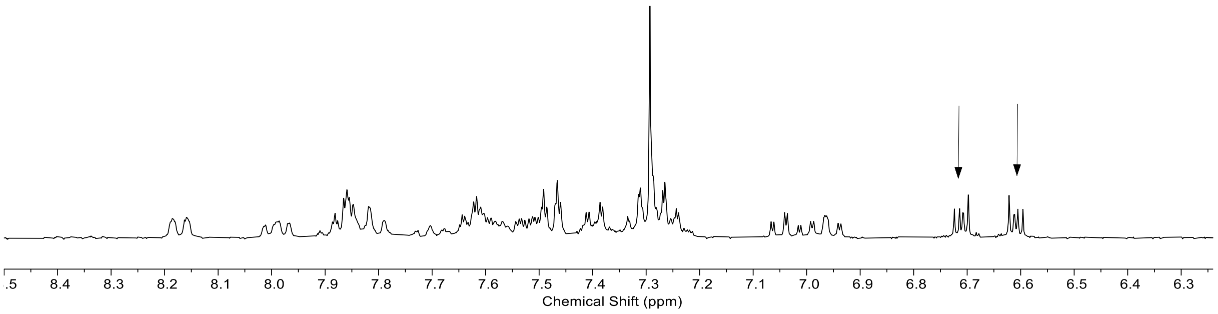

Further analysis of this reaction mixture by $^{31}P\{^1H\}$ NMR spectroscopy (Figure S12) showed two signals at δ_P 77.25 ppm and δ_P 54.72 ppm, the first signal consistent with the starting Au(III) complex **3**. The latter signal, however, is consistent with the formation Au(I) isocyanide complex **S8** which was synthesised independently (δ_P = 54.78 ppm). ^1H NMR spectroscopic measurements of the reaction mixture were inconclusive (Figure S13); however, did show the formation of free biphenylene, presumably from a C–C reductive elimination. Analysis of the reaction mixture by mass spectrometry (ESI$^+$) showed Au(I) complex **S8** to be the dominant molecular ion, with the aurafluorene complex **6** also present. Nanospray mass spectrometric analysis of the former species gave *m*/*z* 701.3307 for $[M–SbF_6]^+$ (calcd. 702.3299).

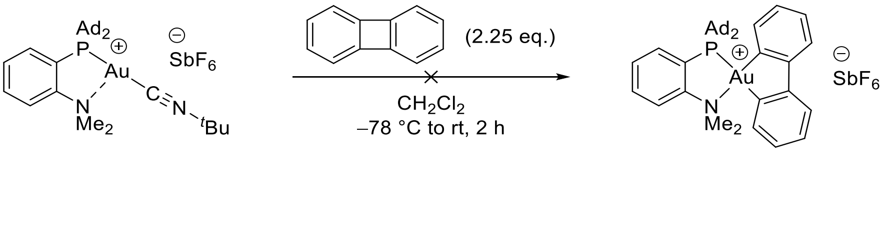

In a reaction of isocyanide complex **S8** and biphenylene under the oxidative addition conditions above, the formation of Au(III) complex **3** was not observed, consistent with irreversible formation of Au(I) isocyanide complex **S8**.

Reaction with 10 eq. *tert*-butyl isocyanide:

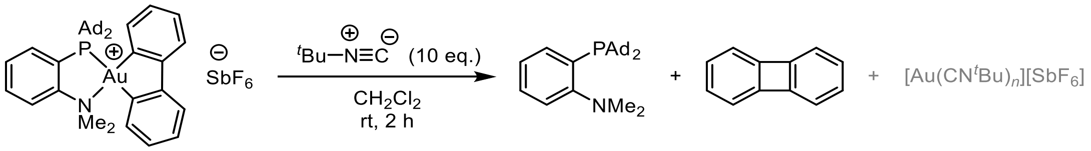

Following the same general procedure above with 10 equivalents of *tert*-butyl isocyanide, no migratory insertion and reductive elimination was observed. However, a different reaction outcome compared to the addition of one equivalent occured. In this case, analysis of the reaction mixture by $^{31}P\{^{1}H\}$ NMR spectroscopy gave one signal at δ_P 20.16 ppm (Figure S14). This combined with the ^{1}H NMR spectrum (Figure S15), obtained after purification by normal phase flash chromatography, is consistent with the formation of free ligand, MeDalPhos.[21] Furthermore, MeDalPhos was crystallized from the reaction mixture; however, full analysis could not be completed due to unresolved electron density on P (Figure S16). Nevertheless, the structure clearly shows the absence of a bound Au atom. It is speculated that the fate of the Au is associated with the formation of a homoleptic Au(I) isocyanide complex, many examples of which have been reported in the literature.[22]

Figure S14: $^{31}P\{^{1}H\}$ NMR spectrum of the reaction mixture of the attempted migratory insertion of *tert*-butyl isocyanide with 20 eq. showing free MeDalPhos ligand.

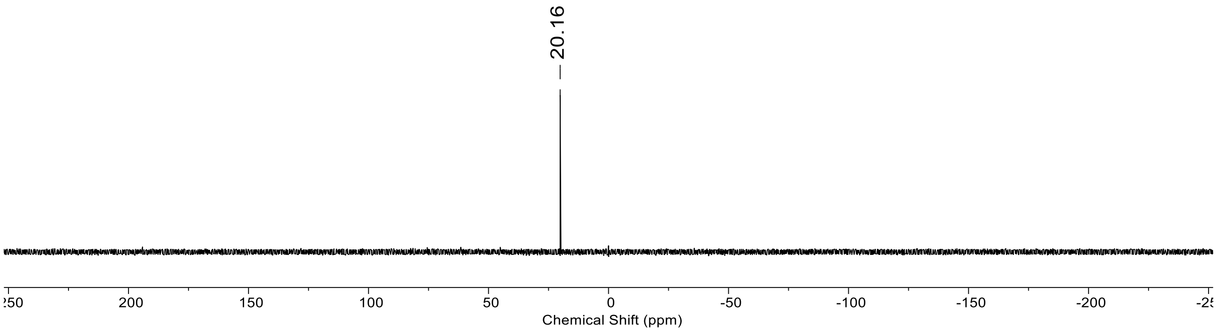

Figure S15: ^{1}H NMR spectrum of isolated material of the above reaction in $CDCl_3$ corresponding to free MeDalPhos ligand.

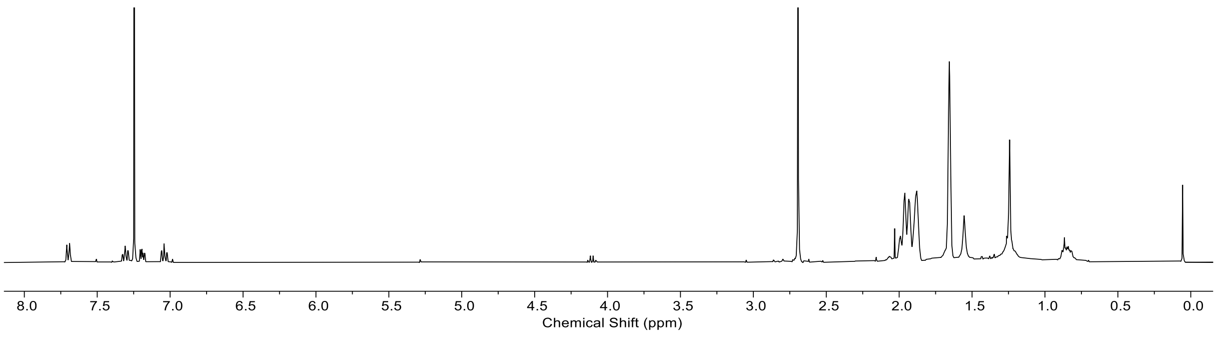

Figure S16: Molecular structure of free MeDalPhos ligand isolated from the above reaction with 20 eq. *tert*-butyl isocyanide determined by scXRD. *N.B. The structure is incomplete due to unresolved electron density on P; however, it does show the absence of Au and is in keeping with the ^{1}H and ^{31}P{^{1}H} NMR data given above.*

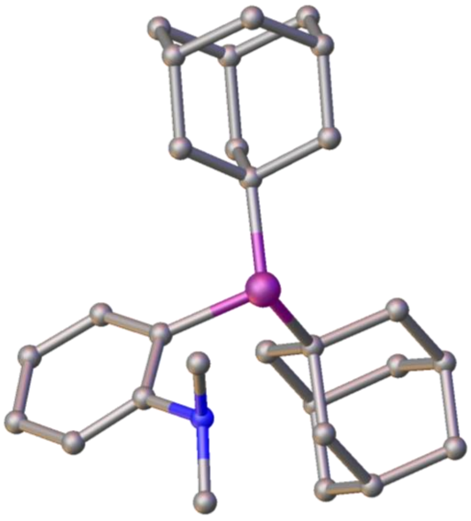

3. Crystallographic information

X-ray diffraction experiments on **S8** and **12** (Figures S17 and S18, respectively) were carried out at 100(2) K on a Bruker APEX II diffractometer using Mo-Ka radiation (λ = 0.71073 Å). Intensities were integrated in SAINT[23] and absorbtion corrections based on equivalent reflections were applied in SADABS[24] for **S8** and TWINABS[25] for **12**.

S8 and **12** was solved using was solved using ShelXT[26] and refined as a two-component twin by full matrix least squares against F^2 in ShelXL[27] using Olex2.[28] **12** was refined as a two-component twin. All of the non-hydrogen atoms were refined anisotropically, while all of the hydrogen atoms were located geometrically and refined using a riding model.

Crystal structure and refinement data are given in Table S1. Crystallographic data for compounds **S8** and **12** have been depositied with the Cambridge Crystallographic Data Centre as supplementary publication. CCDC deposition numbers: 2124037 (**S8**) and 2124038 (**12**). Copies of the data can be obtained free of charge on application to the CCDC, 12 Union Road, Cambridge, CB2 1EZ, U.K. Fax: (+44) 1223 336033. Email: deposit@ccdc.cam.ac.uk.

Figure S17: Thermal ellipsoid plot of **S8**. Thermal ellipsoids are shown at the 50% probability level with the SbF_6 counterion, solvent of crystallization (THF) and hydrogens omitted for clarity. Selected bond lengths (Å) and bond angles (°): Au1-P1 2.2972(7); Au1-C1 1.991(3); C1-N1 1.139(4); P1-Au1-N1 176.24(9). Non-bonding distances (Å): Au1-N2 2.681.

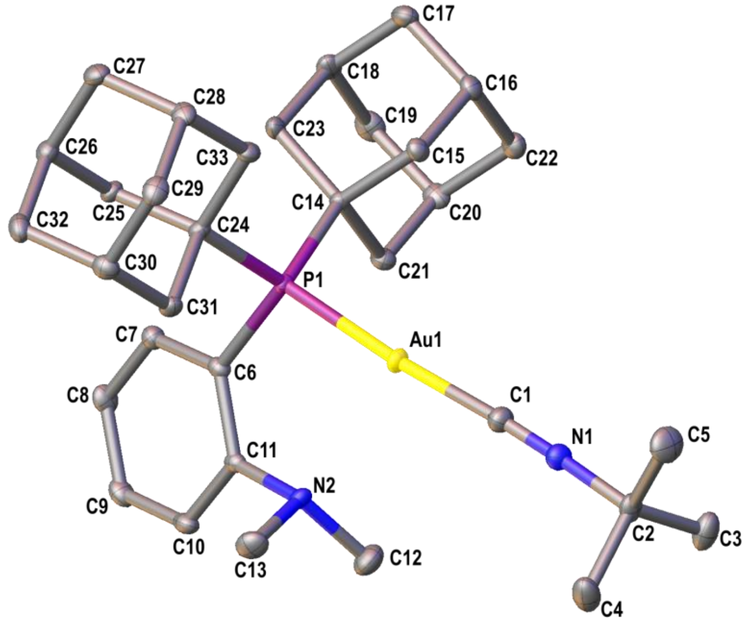

Figure S18: Thermal ellipsoid plot of **12**. Thermal ellipsoids are shown at the 50% probability level with the SbF_6 counterion, solvent of crystallization (CH_2Cl_2) and hydrogens omitted for clarity. Selected bond lengths (Å) and bond angles (°): Au1-C1 2.057(6); Au1-C8 2.084(6); Au1-P1 2.3812(17); Au1-N1 2.260(5); C1-Au1-C8 76.5(2); P1-Au1-N1 83.01(14).

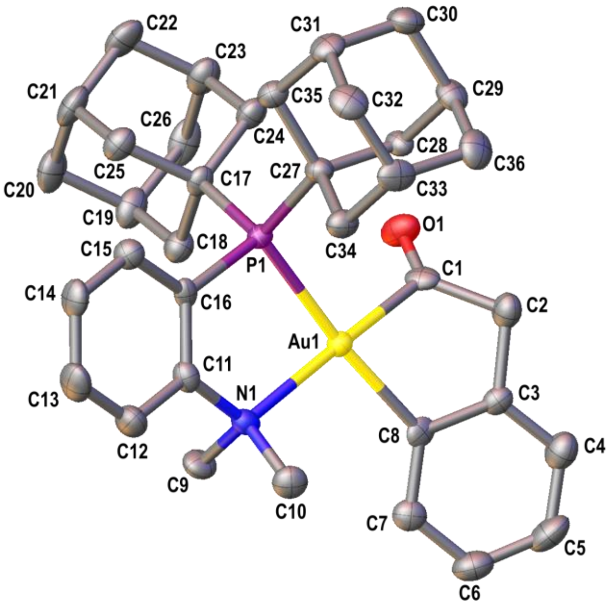

Table S1: Crystal data and structure refinement for **S8** and **12**.

**Identification code**	**S8**	**12**
**Empirical formula**	$C_{37}H_{57}AuF_6N_2OPSb$	$C_{37}H_{48}AuCl_2F_6NOPSb$
**Formula weight**	1009.53	1057.35
**Temperature/K**	100(2)	100(2)
**Crystal system**	triclinic	monoclinic
**Space group**	*P-1*	$P2_1/n$
***a*/Å**	11.6179(2)	11.8557(4)
***b*/Å**	17.7462(4)	14.3296(4)
***c*/Å**	19.9736(4)	22.4007(9)
**α/°**	101.9460(10)	90
**β/°**	90.9070(10)	91.370(3)
**γ/°**	106.8260(10)	90
**Volume/Å^3**	3843.73(14)	3804.5(2)
**Z**	4	4
**ρ_{calc}g/cm^3**	1.745	1.846
**μ/mm^{-1}**	4.618	4.805
**F(000)**	2000.0	2072.0
**Crystal size/mm^3**	0.367 × 0.29 × 0.061	0.244 × 0.129 × 0.03
**Radiation**	MoKα (λ = 0.71073)	MoKα (λ = 0.71073)
**2θ range for data collection/°**	2.09 to 56.002	3.638 to 55.026
**Index ranges**	-15 ≤ h ≤ 15, -23 ≤ k ≤ 23, -26 ≤ l ≤ 26	-15 ≤ h ≤ 15, 0 ≤ k ≤ 18, 0 ≤ l ≤ 29
**Reflections collected**	70753	11069
**R_{int} / R_{sigma}**	0.0336 / 0.0323	/ 0.0691
**Data/restraints/parameters**	18531/136/930	11069/378/546
**Goodness-of-fit on F^2**	1.027	1.019
**Final R indexes [I>=2σ (I)]**	R_1 = 0.0243, wR_2 = 0.0456	R_1 = 0.0446, wR_2 = 0.0768
**Final R indexes [all data]**	R_1 = 0.0419, wR_2 = 0.0501	R_1 = 0.0698, wR_2 = 0.0835
**Largest diff. peak/hole / e Å^{-3}**	0.81/-0.78	1.19/-1.45

4. Mechanistic studies

The mechanistic studies in this Section provide evidence for the proposed carbonylation pathway. A summary of the proposed mechanism is given in Scheme S1. Aurafluorene complex **6** most likely exists in equilibrium with Au(I) complex **7** *via* a C–C reductive elimination. This was shown when isolated after the carbonylation reaction in trace yield with MeDalPhos and in 32% when the IPr-derived biphenylene complex **4** was used. Existence of an equilibrium was also shown in a competition experiment outlined in Section 4.1 with complex **6** and biphenylene-d_8 (**2g**). Upon addition of carbon monoxide, bound complex **8** is intially formed shown by FTIR spectroscopy in Section 4.2 and $^{31}P\{^{1}H\}$ NMR spectroscopy in Section 4.3. Carbon monoxide can then undergo a migratory insertion forming complex **9**. Evidence from ^{13}C-labelling and mass spectrometry in Sections 4.3 and 4.4 respectively are consistent with complex **9**. Reductive elimination occurs forming 9-fluorenone **4** and Au(I) complex **10**, which was also directly observed by nanospray mass spectrometry (Section 4.4).

Scheme S1: Summary of proposed carbonylation mechanism forming 9-fluorenone.

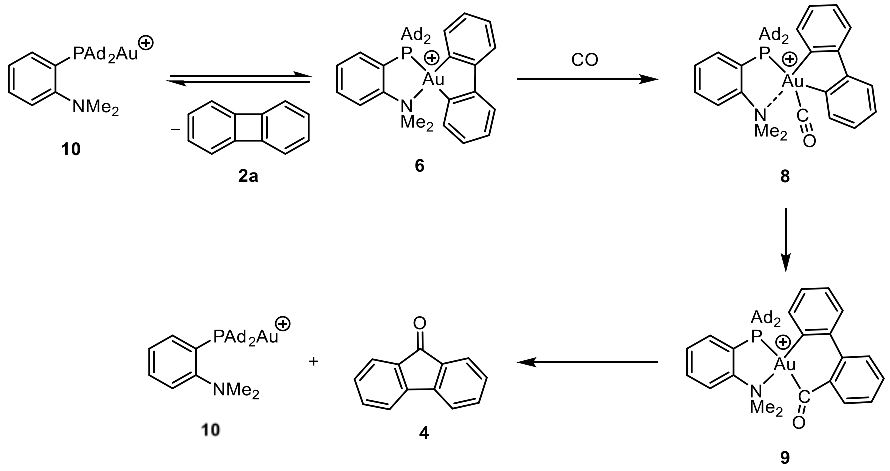

4.1 *Biphenylene-d_8 cross-over experiment*

To a solution of MeDalPhos aurafluorene complex **6** (27 mg, 0.0268 mmol) in dichloromethane (2.5 mL), biphenylene-d_8 (**2g**, 4 mg, 0.0268 mmol) was added. After stirring at room temperature for two hours, an aliquot was taken for analysis by nanospray mass spectrometry. The nanospray mass spectrum obtained (Figure S19) shows the cross-over between the Au(III) biphenylene complex and biphenylene-d_8.

Figure S19: Nanospray mass spectrum of cross-over experiment reaction mixture.

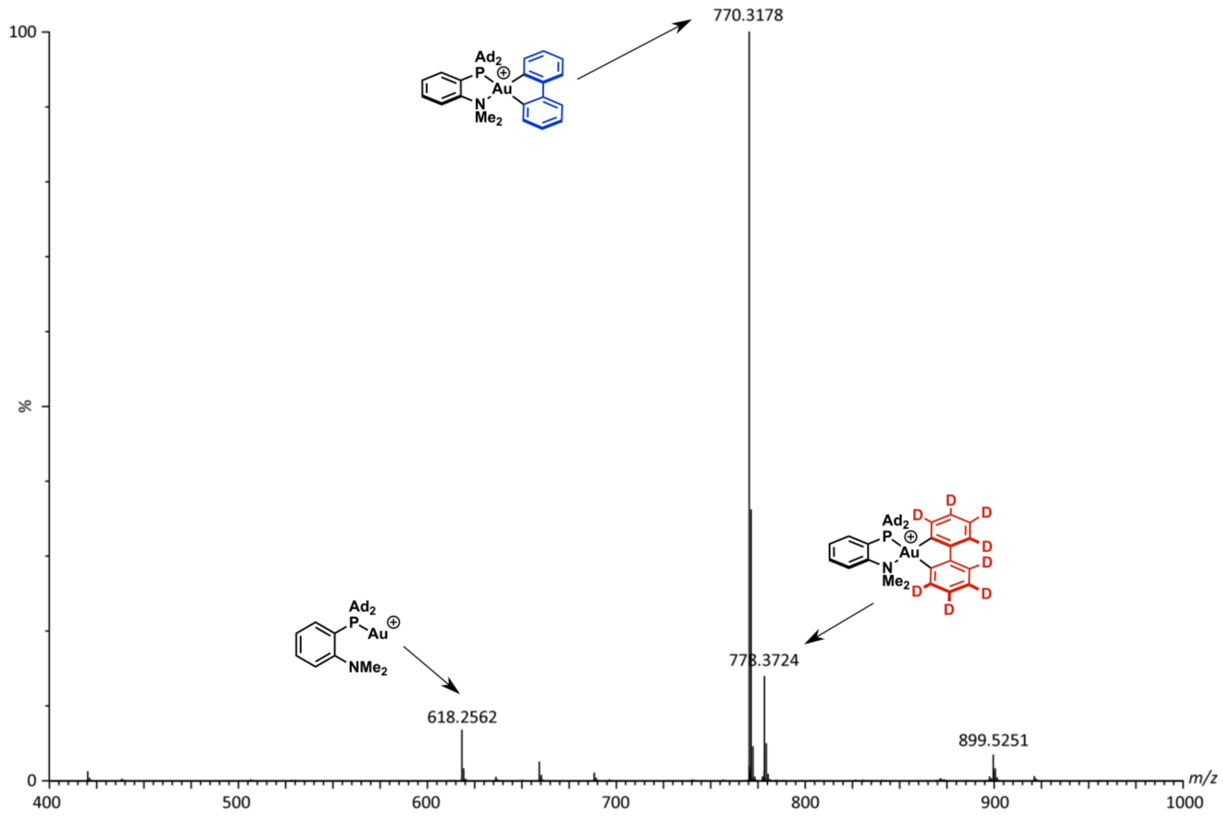

4.2 *FTIR reaction mixture analysis*

Following the general carbonylation procedure above on a 0.0612 mmol scale, the autoclave was cooled and vented after five hours. An aliquot of the reaction mixture was taken (0.5 mL) ***immediately*** and diluted with dichloromethane (5 mL). The resultant solution was added to a CaF_2 IR solution cell and the spectrum given in Figure S20 was collected.

Figure S20: FTIR spectrum of the carbonylation reaction mixture.

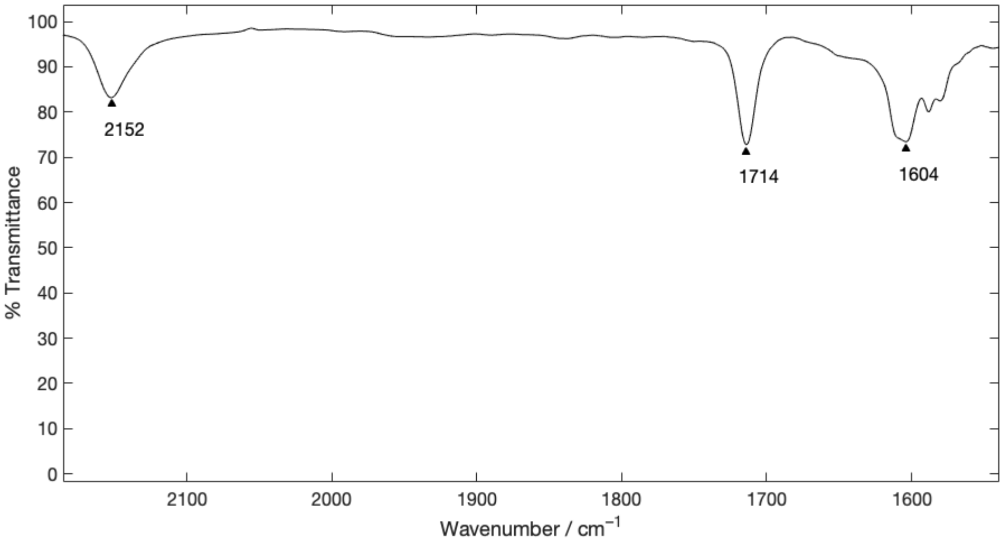

Signal at ν = 2152 cm^{-1} is consistent with a bound carbon monoxide complex *cf.* complex **8** in Scheme S1. It was not possible to assign signals at ν = 1714 cm^{-1} and ν = 1604 cm^{-1}; however, these are most likely related to the product **4** and/or migratory insertion complex **9**.

4.3 ^{13}C-labelling NMR study

Following a procedure similar to the general carbonylation procedure above, MeDalPhos aurafluorene complex **6** (40 mg, 0.0612 mmol) was added to a 100 mL stainless steel autoclave with a PTFE insert and dissolved in dichloromethane (4 mL). The autoclave was then sealed and cooled to –130 °C (pentane/N_2). Once cooled, the autoclave was pressurised to 4 bar of ^{13}C-labelled carbon monoxide. The autoclave was allowed to *SLOWLY* warm to room temperature, during which time the pressure increased to 8 bar. The reaction mixture was heated at 50 °C for five hours, cooled on ice and vented. An aliquot of the reaction mixture (0.5 mL) was taken and ***immediately*** subjected to analysis by ^{13}C NMR spectroscopy to give the spectrum in Figure S22. The remaining reaction mixture was evaporated *in vacuo* and purified by normal phase flash chromatography (0 to 10% ethyl acetate in hexane) to give ^{13}C-labelled 9-fluorenone (Figure S21); **^{1}H NMR (500 MHz, $CDCl_3$)** δ 7.67 (dd, *J* = 7.4, 3.0 Hz, 2H, C_{Ar}-*H*), 7.56 – 7.45 (m, 4H, C_{Ar}-*H*), 7.30 (tt, *J* = 7.4, 1.1 Hz, 2H, C_{Ar}-*H*); **^{13}C{^{1}H} NMR (126 MHz, $CDCl_3$)** δ 194.1 (*C*=O), 144.6 (d, *J* = 8.0 Hz, *C*$_{Ar}$), 134.8 (*C*$_{Ar}$), 134.3 (d, *J* = 55.2 Hz, *C*$_{Ar}$), 129.2 (d, *J* = 3.7 Hz, *C*$_{Ar}$), 124.5 (d, *J* = 2.6 Hz, *C*$_{Ar}$), 120.5 (d, *J* = 3.8 Hz, *C*$_{Ar}$); **GC-MS (EI$^+$)**

calcd. 181.1, found 181.0 (100%), 152.0 (42%), 151.0 (24%), 76.0 (15%), 150.0 (15%), 182.0 (14%), 126.0 (8%), 63.0 (7%), 75.0 (7%), 183.0 (5%).

Figure S21: $^{13}C\{^1H\}$ NMR spectrum of ^{13}C-enriched 9-fluorenone.

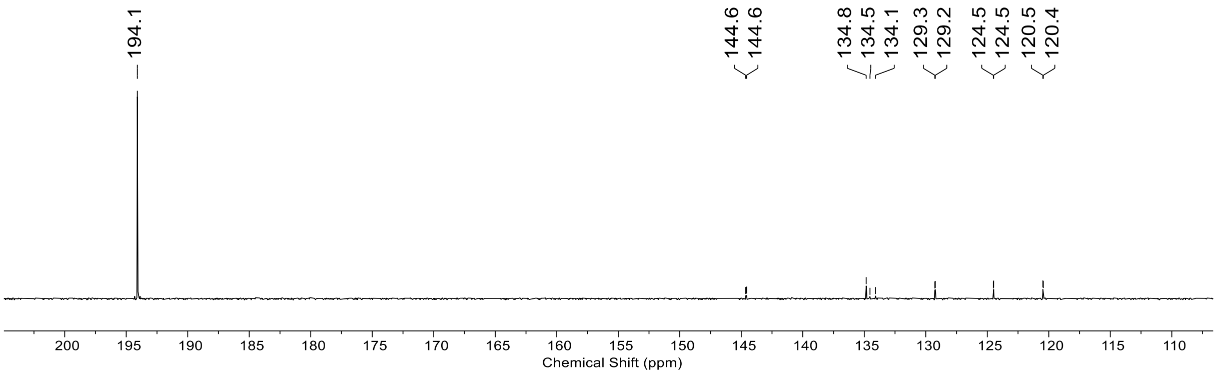

Figure S22: $^{13}C\{^1H\}$ NMR spectrum of the carbonylation reaction mixture with ^{13}C-labelled carbon monoxide.

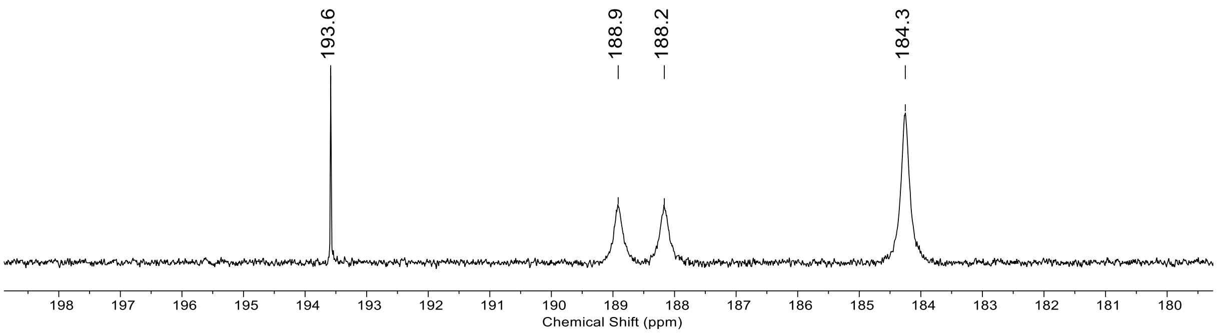

Figure S23: $^{31}P\{^1H\}$ NMR spectrum of the carbonylation reaction mixture with ^{13}C-labelled carbon monoxide.

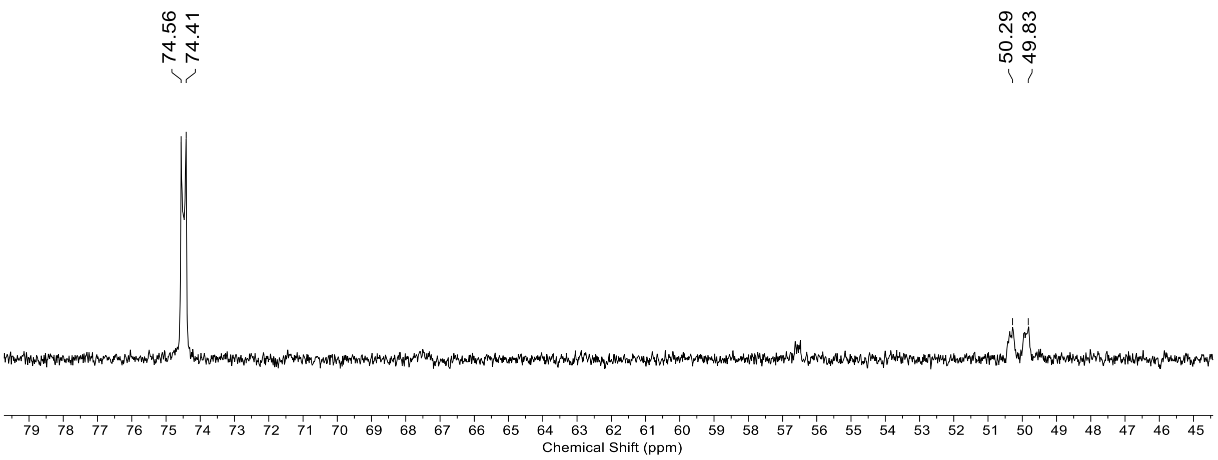

Three signals are observed in the $^{13}C\{^1H\}$ NMR spectrum given in Figure S22. The signal at δ_C = 193.6 ppm corresponds to the product, 9-fluorenone. The signal at δ_C = 184.3 ppm either corresponds to free carbon monoxide or a bound species *cf.* complex **8**. The doublet at δ_C = 188.5 ppm (d, J = 94.3 Hz) is consistent with migratory insertion complex **9**.

Two major signals are observed in the $^{31}P\{^1H\}$ NMR spectrum given in Figure S23. The signal at δ_P = 74.49 ppm (d, J = 30.0 Hz) is consistent with bound carbon monoxide complex **8**. The signal at δ_P = 50.06 ppm (d, J = 93.1 Hz) corresponds to the migratory insertion complex **9**. The coupling observed here in the $^{31}P\{^1H\}$ NMR spectrum directly corresponds to that observed in the $^{13}C\{^1H\}$ NMR spectrum in Figure S22.

4.4 Reaction mixture analysis by nanospray mass spectrometry

Following a procedure identical to that described in Section 4.2, after five hours heating at 50 °C, an aliquot was taken for analysis by nanospray mass spectrometry.

Figure S24: Nanospray mass spectrum of the carbonylation reaction mixture showing migratory insertion complex **13**.

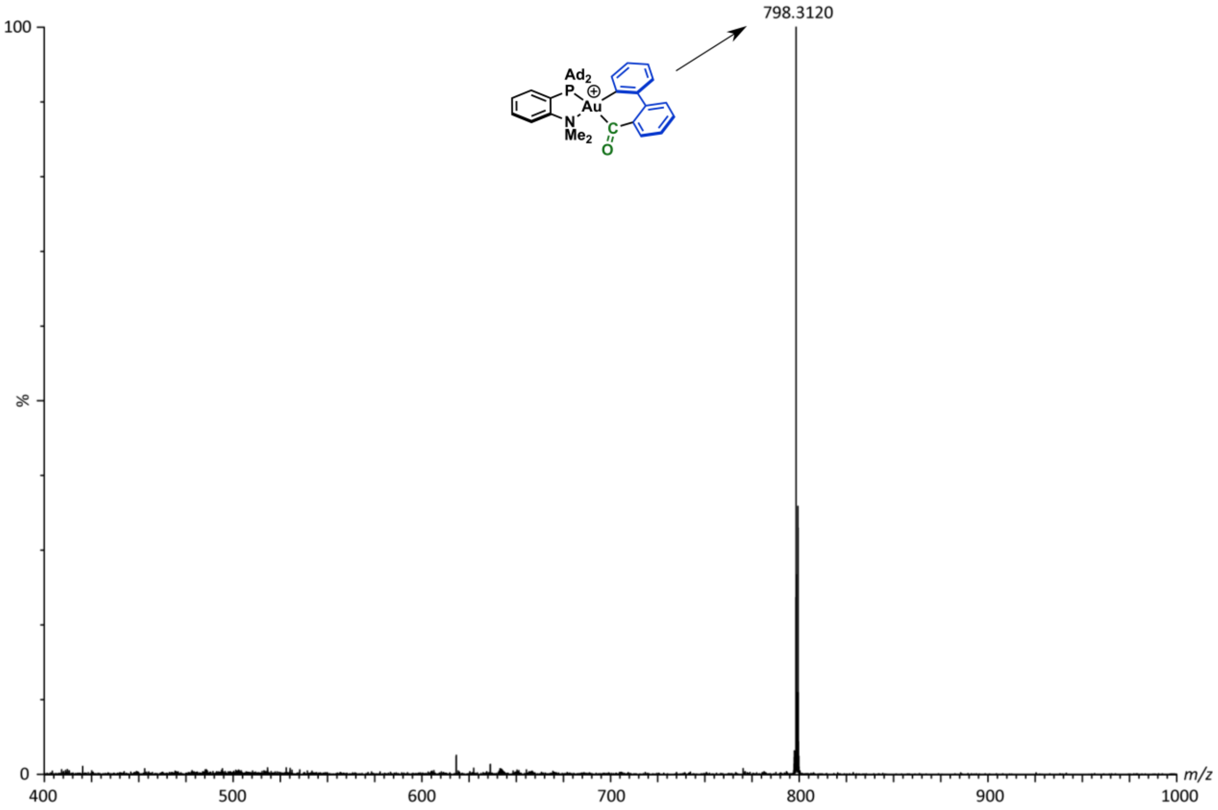

Figure 25: Nanospray MS/MS of *m/z* 798.3120.

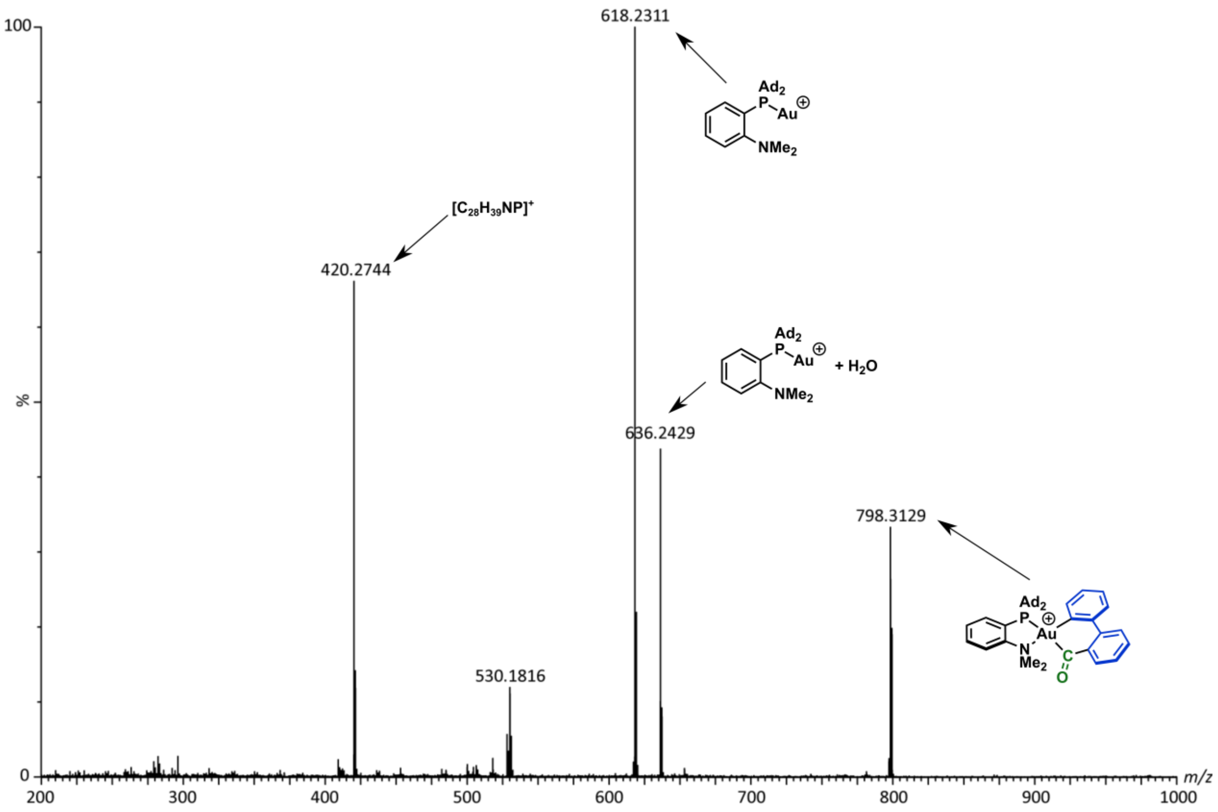

4.5 *CO incompatability with oxidative addition (^{31}P NMR monitoring)*

Arrays of $^{31}P\{^1H\}$ NMR spectra were collected at 202 MHz on a Varian VNMRS 500 MHz spectrometer and were implemented using the standard Varian software. Triphenylphosphate was used as an internal standard which was weighed directly into the oxidative addition reaction mixture (see below). Each acquisition had 8 scans per spectrum, a pulse angle of 60° and a relaxation delay of 20 s. An acquisition was run every 3 minutes. NMR array data was processed using *MestReNova 14.0*. Spectrometer probe temperature was periodically calibrated using the ethylene glycol/methanol-thermometer methods.[29]

MeDalPhosAuCl (20 mg, 0.030 mmol) and $AgSbF_6$ (10 mg, 0.030 mmol) were pre-mixed in a vial in dichloromethane (0.5 mL) in air. After five minutes, the mixture was cooled to –78 °C and a solution of biphenylene (10 mg, 0.068 mmol) and triphenylphosphate internal standard (10 mg, 0.030 mmol) was added. After stirring for one minute, the reaction mixture was filtered (using a Millipore Millex-HV 0.45 μm PVDF membrane syringe filter) directly into a J. Young's valve NMR tube at –78 °C. The tube was either under an atmosphere of dinitrogen or was pressurized to 2 bar of carbon monoxide at this point. The tube was sealed and the contents

were mixed by shaking. The sample was then removed from the cold bath and manually loaded into the NMR spectrometer with the probe temperature set at 25 °C. The spectrometer was pre-tuned to ^{31}P and shimmed on a sample of triphenylphosphate in dichloromethane. The kinetics experiment was started immediately without tuning or shimming. The time between removing the NMR tube from the cold bath and the middle of the first acquisition (*i.e.*, 4th scan, typically <30 s) were measured using a stopwatch.

Figure S26: ^{31}P NMR reaction monitoring of the oxidative addition of biphenylene with MeDalPhosAuCl under (A) dinitrogen (1 atm.) and (B) 2 bar carbon monoxide. Curves in (A) have been fitted with arbitrary polynomial functions.

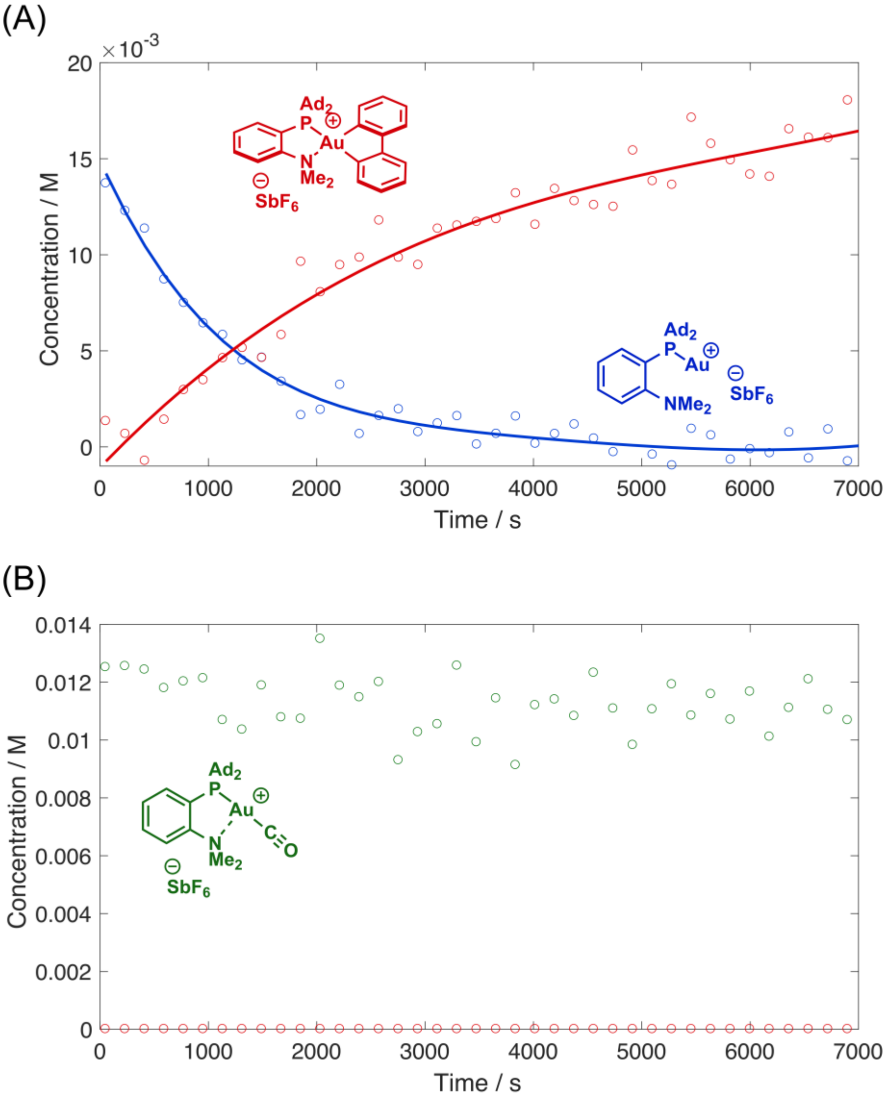

The MeDalPhosAu(I) CO complex was also observed by nanospray mass spectrometry giving *m*/*z* 646.2503 for $[M{-}SbF_6]^+$ $[C_{29}H_{40}AuNOP]^+$, calcd. *m*/*z* 646.2513.

4.6. *Benzocyclobutenone competition experiment*

Complex **12** (12 mg, 0.0123 mmol) was dissolved in 1,2-dichlorobenzene (1.2 mL) and naphthyl benzocyclobutenone (**2i**, 2 mg, 0.0123 mmol) was added. After stirring at 130 °C for four hours, an aliquot (*approx.* 0.05 mL) was taken for analysis by nanospray mass spectrometry. The remainer of the reaction mixture was evaporated *in vacuo*, re-dissolved in CD_2Cl_2 and subjected to analysis by ^1H NMR spectroscopy (Figure S27).

Similarly to the isomerization reactions in Section 2.2.3, a significant amount of decomposition was observed which gave rise to a complicated array of signals in the ^1H NMR spectrum. However, imporatantly, doublets associated with the isomerized complex CH_2 signals were observed. In total, four doublets were observed in the δ_H = 4 – 5 ppm region (Figure S27). The doublets at δ_H = 4.69 ppm and δ_H = 4.04 ppm were assigned to be isomerized complexes **13a** and **13b**. The doublets at δ_H = 4.91 ppm and δ_H = 4.24 ppm were assigned to be isomerized complexes **S11a** and **S11b**. (See Section 2.2.3 for details on assignment). These results show a clear cross-over resulting in the formation of both isomerization complexes. Additionally, analysis by nanospray mass spectrometry also showed the cross-over. These result combined provide evidence for a reductive elimination, re-oxidative addition pathway for isomerization as opposed to one involving retro-carbonylation.

Figure S27: ^1H NMR spectrum of the benzocyclobutenone competition experiment reaction mixture.

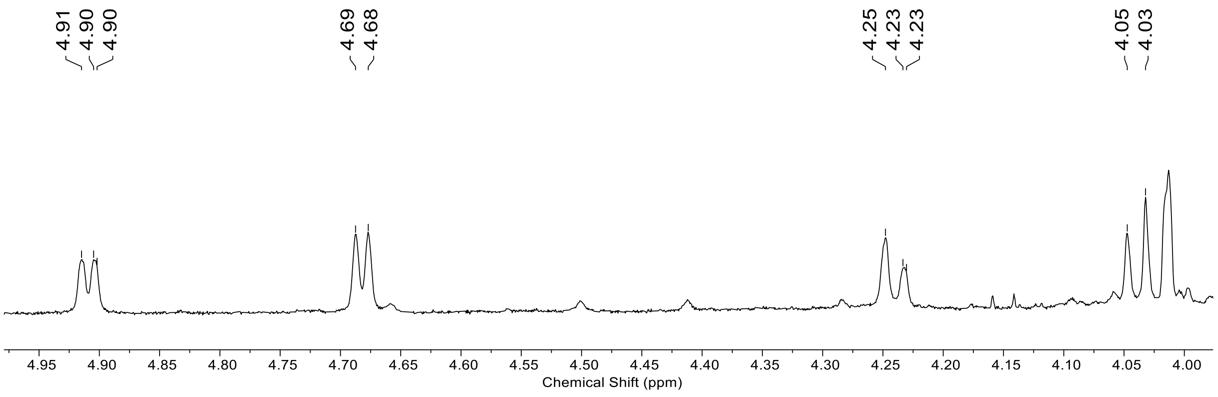

Figure S28: Nanospray mass spectrum of benzocyclobutenone complex competition experiment. Only one isomer for each complex is shown.

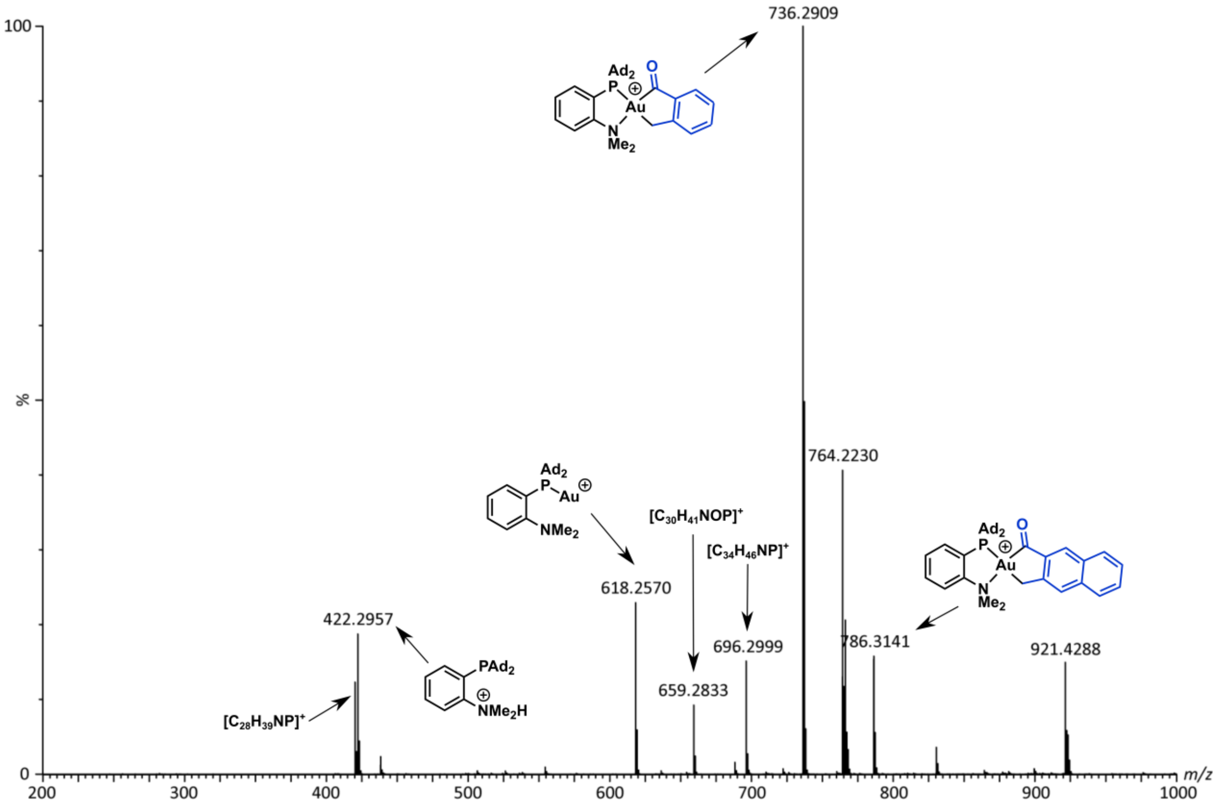

Scheme S2: Isomerization of complex **12** under an atmosphere of ^{13}CO (1 bar), other product isomer not shown.

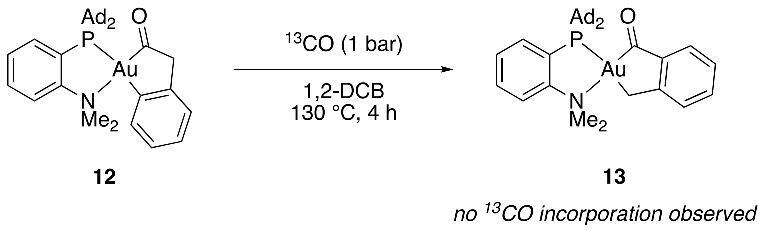

Performing the isomerization of acyl complex **12** under an atmosphere of ^{13}CO (1 bar) did not show incorporation of ^{13}CO in either product isomers by ^{13}C{^{1}H} NMR spectroscopy. This provides further evidence that a retro-carbonylation (Pathway A in manuscript Scheme 5) is unlikely to be operative and isomerization takes place *via* C–C reductive elimination and re-oxidative addition (Pathway B).

5. Computational details

5.1. *General considerations*

All calculations were performed using *Gaussian 09, Revision D.01*.[30] The ωB97-XD functional[31] with an ultrafine integration grid was used throughout with Ahlrichs' def2-TZVP basis set on Au; def2-SVP on C, P, N and def2-SV on all other atoms.[32] The 60-electron def2 pseudopotential was used for Au.[33] Solvation (dichloromethane) was modelled using the SMD model.[34] The nature of all stationary points was confirmed by analysis of the harmonic vibrational frequencies. All calculations were performed at standard temperature and pressure (298.15 K and 1 atm.).

5.2. *Oxidative addition of cyclopropane-containing substrates*

5.2.1 *Potential energy surfaces*

To computationally assess the apparent lack of activity of cyclopropane-containing substrates, alkylidene cyclopropane **2d** was selected. This type of substrate is known to undergo oxidative insertion into the proximal or distal C–C bond. The potential energy surface given in Figure S29 shows insertion into the proximal C–C bond.

Figure S29: Calculated potential energy surface for the proximal C–C bond insertion for alkylidene cyclopropane **2d**.

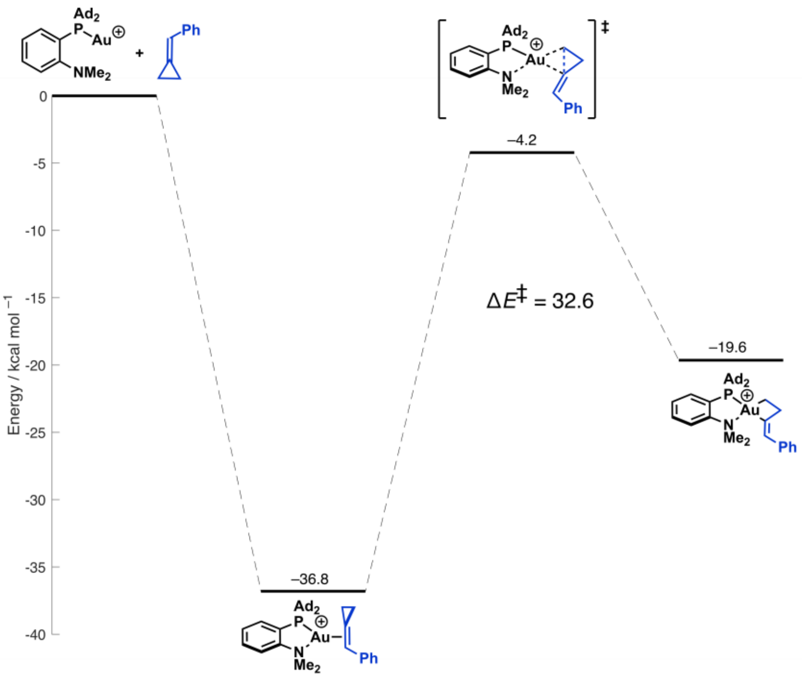

Oxidative addition into the proximal C–C bond is preceeded by formation of a π-complex with the MeDalPhosAu$^+$ fragment. Complexes of this type have been observed by mass spectrometry (see above). The barrier for this process is $\Delta E^{\ddagger}$ = 32.6 kcal mol^{-1} and is endothermic by ΔE = 17.2 kcal mol^{-1} with respect to the π-complex.

Figure S30: Calculated potential energy surface for the distal C–C bond insertion for alkylidene cyclopropane **2d**.

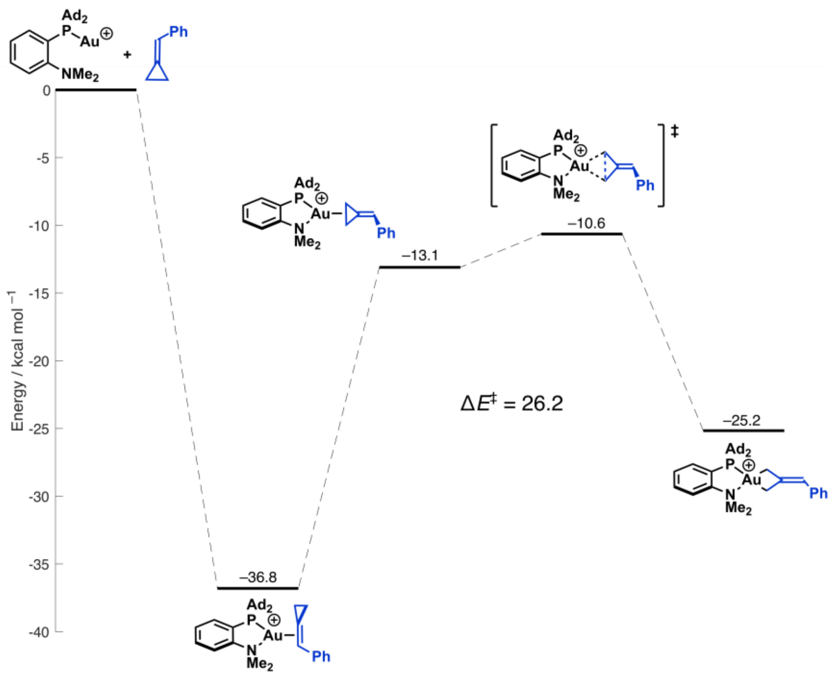

For insertion into the distal C–C bond, a similar potential energy surface was calculated (Figure S30). After forming an initial π-complex, a σ-complex can be formed prior to oxidative addition. The barrier for insertion into this C–C bond is lower than the proximal example giving $\Delta E^{\ddagger}$ = 26.2 kcal mol^{-1} from the initial π-complex. The overall transformation is also endothermic (ΔE = 11.6 kcal mol^{-1}).

Overall, oxidative addition looks kinetically feasible for alkylidene cyclopropane **2d**. However, the key impediment to this C–C bond activation appears to be linked to the formation of thermodynamically unfavorable 4-membered metallocycles (see reference 20 in the manuscript for examples of aurocyclobutanes formed using different methods).

5.2.2. Energies (au) and Cartesian coordinates (Å) for stationary points

MeDalPhosAu⁺

SCF = –1621.63193
ZPE = –1620.99290

Au	0.21020	-1.21760	-1.79350
N	0.96980	-3.03980	0.21040
P	-0.03470	0.00620	0.12070
C	0.15510	-4.21340	-0.06840
C	2.39100	-3.28360	0.01510
C	0.55680	-2.65030	3.89700
C	0.11030	-1.33430	4.00340
C	-0.05020	-0.57080	2.85320
C	0.22490	-1.08740	1.57390
C	0.67160	-2.42440	1.47040
C	0.83010	-3.17990	2.64160
H	0.69260	-3.26460	4.79160
H	-0.11470	-0.89790	4.98020
H	-0.40680	0.45350	2.96340
H	1.17980	-4.21260	2.55840
H	2.79220	-4.06850	0.69300
H	2.56720	-3.60820	-1.02490
H	2.95860	-2.35550	0.18560
H	0.39570	-5.08160	0.58250
H	-0.91180	-3.96690	0.05900
H	0.31420	-4.52490	-1.11520
C	3.81010	1.60710	-0.09200
C	3.55940	2.61550	-1.22240
C	2.24110	3.35700	-0.96050
C	1.07890	2.35210	-0.92230
C	1.31060	1.31550	0.19940
C	2.65730	0.59190	-0.05110
H	4.75020	1.06120	-0.27930
H	3.51300	2.09180	-2.19470
H	4.39470	3.33630	-1.27900
H	2.05190	4.08020	-1.77140
H	0.13770	2.89890	-0.74640
H	0.98880	1.84190	-1.89890
H	2.62210	0.03720	-1.00640
H	2.83720	-0.14500	0.75190
C	2.31500	4.09150	0.38560
H	1.37170	4.63700	0.57120
H	3.12710	4.84050	0.36600
C	3.89380	2.34550	1.25060
H	4.09790	1.62870	2.06660
H	4.72780	3.06990	1.23510
C	1.42090	2.04730	1.55610
H	1.63330	1.32370	2.35930
H	0.48020	2.56000	1.80780
C	2.56770	3.07350	1.50620
H	2.60810	3.59210	2.47900
C	-3.49500	2.29100	1.13400
C	-3.89710	2.72460	-0.28100
C	-3.70430	1.54330	-1.24070
C	-2.23270	1.10080	-1.23350
C	-1.80190	0.66800	0.19220
C	-2.01490	1.86890	1.13990
H	-3.61520	3.13550	1.83330
H	-3.28070	3.58290	-0.60490
H	-4.95130	3.05510	-0.29290
H	-3.96950	1.84750	-2.26740
H	-2.10580	0.25630	-1.93400
H	-1.59510	1.92280	-1.59740
H	-1.39640	2.72060	0.81470
H	-1.72040	1.61880	2.17050
C	-4.58750	0.36840	-0.80080
H	-4.47040	-0.47720	-1.50290
H	-5.65170	0.66440	-0.81960
C	-4.37220	1.11450	1.58570
H	-4.09740	0.80690	2.61120
H	-5.43320	1.42140	1.60960
C	-2.71350	-0.50190	0.63470
H	-2.44910	-0.83100	1.65290
H	-2.57030	-1.36850	-0.03800
C	-4.18610	-0.06180	0.61640
H	-4.80870	-0.91320	0.93920

Alkylidene cyclopropane (ACP)

SCF = –386.61024
ZPE = –386.44204

C	0.99880	-0.98370	-0.00010
C	2.12930	-0.28170	-0.00100
H	1.06820	-2.07880	0.00050
C	3.59560	-0.28650	0.00060
C	2.80060	1.02200	-0.00010
H	4.12130	-0.56630	-0.92200
H	4.11920	-0.56720	0.92420
H	2.78670	1.61740	0.92280
H	2.78940	1.61750	-0.92290
C	-3.01820	0.53740	0.00010
C	-2.77330	-0.83610	0.00000
C	-1.46260	-1.31300	-0.00010
C	-0.36960	-0.43110	-0.00010
C	-0.63180	0.95050	0.00000

C	-1.94050	1.42730	0.00010
H	-4.04500	0.91510	0.00020
H	-3.60820	-1.54360	0.00010
H	-1.27780	-2.39230	-0.00010
H	0.19980	1.66040	0.00000
H	-2.12300	2.50630	0.00020

MeDalPhosAu(ACP) η^2 intermediate

SCF = –2008.30459
ZPE = –2007.49359

N	-1.16870	-1.07890	2.15390
P	1.00320	0.21870	0.34560
C	-2.40880	-0.37010	2.51250
C	-1.29350	-2.52460	2.39970
C	1.04990	-0.02150	4.98080
C	2.08020	0.63910	4.31290
C	2.06080	0.71030	2.92490
C	1.02420	0.12950	2.17450
C	-0.02660	-0.51190	2.85760
C	0.00620	-0.58580	4.25630
H	1.05160	-0.09100	6.07210
H	2.89900	1.10070	4.87110
H	2.86840	1.23880	2.41620
H	-0.80230	-1.08690	4.79430
H	-1.48880	-2.75010	3.46530
H	-2.13160	-2.92060	1.80510
H	-0.36660	-3.03220	2.09040
H	-2.65240	-0.48130	3.58640
H	-2.30010	0.70080	2.28050
H	-3.24520	-0.77670	1.92280
C	3.35700	-3.09390	-0.52620
C	3.43170	-2.90220	-2.04770
C	3.72900	-1.42920	-2.36210
C	2.61070	-0.53940	-1.79760
C	2.50470	-0.72160	-0.26710
C	2.22880	-2.21620	0.03770
H	3.13210	-4.14830	-0.29210
H	2.47690	-3.20520	-2.51520
H	4.22200	-3.54690	-2.47250
H	3.77630	-1.28260	-3.45440
H	2.83430	0.51300	-2.03850
H	1.64830	-0.79150	-2.27880
H	1.26810	-2.52450	-0.41430
H	2.14360	-2.36750	1.12930
C	5.06390	-1.02000	-1.72340
H	5.28720	0.03690	-1.95810
H	5.88730	-1.62800	-2.13950
C	4.69460	-2.69280	0.11020
H	4.65710	-2.84790	1.20380
H	5.50640	-3.33100	-0.28330
C	3.85370	-0.34670	0.38180
H	3.80940	-0.52010	1.46880
H	4.08520	0.71830	0.22090
C	4.98050	-1.21790	-0.20350
H	5.93260	-0.91530	0.26440
C	2.08780	4.25800	-0.44530
C	1.53240	4.34990	-1.87340
C	0.17810	3.63000	-1.93860
C	0.35760	2.15440	-1.55170
C	0.93820	2.03670	-0.12060
C	2.28730	2.77950	-0.06760
H	3.06480	4.76770	-0.39130
H	2.24150	3.89060	-2.58620
H	1.41370	5.40850	-2.16640
H	-0.22140	3.67710	-2.96590
H	-0.62000	1.64430	-1.59270
H	1.01560	1.65540	-2.28160
H	3.00040	2.31830	-0.77030
H	2.72570	2.71530	0.94170
C	-0.80820	4.28960	-0.96550
H	-1.79100	3.78600	-1.02250
H	-0.96600	5.34780	-1.24160
C	1.10500	4.91500	0.53440
H	1.50490	4.86500	1.56350
H	0.97880	5.98370	0.28400
C	-0.05770	2.71970	0.84880
H	0.31570	2.66310	1.88470
H	-1.03200	2.19480	0.82120
C	-0.24940	4.19440	0.46070
H	-0.95620	4.65490	1.17150
Au	-1.01570	-0.89040	-0.23830
C	-2.79140	-1.71330	-1.17960
C	-1.84700	-1.31440	-2.13860
H	-2.84480	-2.78250	-0.93390
C	-1.15770	-1.91570	-3.30390
C	-1.77970	-0.52820	-3.39050
H	-1.66310	-2.74850	-3.80780
H	-0.06370	-1.97470	-3.30360
H	-1.09630	0.32600	-3.44900
H	-2.71600	-0.40710	-3.94940
C	-6.24540	0.54040	0.03710
C	-6.13760	-0.81830	0.33470
C	-5.01540	-1.53880	-0.07510
C	-3.98460	-0.91700	-0.79390
C	-4.10530	0.45210	-1.09010
C	-5.22370	1.17170	-0.67740
H	-7.12290	1.10800	0.36010
H	-6.93260	-1.32290	0.89170
H	-4.93350	-2.60320	0.16760

H	-3.31020	0.96240	-1.64140
H	-5.29810	2.23720	-0.91470

MeDalPhosAu(ACP) proximal TS

SCF = –2008.25136
ZPE = –2007.44167

N	1.04410	-0.37450	2.29530
P	-1.27460	0.12870	0.25300
C	1.48760	-1.71650	2.67260
C	2.06560	0.63000	2.57290
C	-1.54910	0.34050	4.88970
C	-2.67720	0.59250	4.10960
C	-2.57900	0.53070	2.72450
C	-1.36790	0.21860	2.08260
C	-0.23250	-0.03800	2.87710
C	-0.34320	0.02860	4.27340
H	-1.60780	0.38550	5.98100
H	-3.63480	0.83560	4.57780
H	-3.47320	0.72310	2.13050
H	0.53710	-0.17140	4.89010
H	2.31280	0.70030	3.65230
H	2.98840	0.36530	2.03160
H	1.72420	1.61740	2.22400
H	1.70620	-1.80610	3.75660
H	0.71170	-2.45300	2.40830
H	2.40850	-1.96190	2.11840
C	-0.18550	4.08550	-0.45560
C	-0.25550	4.05400	-1.98940
C	-1.54100	3.33750	-2.42590
C	-1.53830	1.89590	-1.89390
C	-1.46210	1.89740	-0.35090
C	-0.17550	2.64590	0.08110
H	0.74300	4.58720	-0.13430
H	0.62780	3.53130	-2.39990
H	-0.24140	5.08340	-2.39080
H	-1.59530	3.30590	-3.52730
H	-2.45760	1.38720	-2.22860
H	-0.68030	1.33950	-2.31530
H	0.71800	2.12100	-0.30410
H	-0.10170	2.65650	1.18330
C	-2.76390	4.07840	-1.86600
H	-3.69270	3.57270	-2.18780
H	-2.79800	5.10860	-2.26410
C	-1.40310	4.83540	0.10140
H	-1.34910	4.88130	1.20440
H	-1.41010	5.87620	-0.26950
C	-2.66890	2.67260	0.22070
H	-2.59950	2.71890	1.31970
H	-3.61570	2.17140	-0.03210
C	-2.68320	4.10920	-0.33330
H	-3.56470	4.63100	0.07610
C	-5.05780	-1.50120	-0.88300
C	-4.66500	-1.99610	-2.28120
C	-3.26310	-2.61660	-2.21980
C	-2.25100	-1.56520	-1.73900
C	-2.63580	-1.03860	-0.33260
C	-4.05280	-0.43330	-0.41670
H	-6.06100	-1.04400	-0.91720
H	-4.67710	-1.15530	-2.99870
H	-5.39600	-2.74300	-2.64000
H	-2.96060	-2.95830	-3.22440
H	-1.24840	-2.02180	-1.70120
H	-2.20320	-0.73640	-2.46390
H	-4.06360	0.40730	-1.12960
H	-4.37260	-0.03960	0.56100
C	-3.26200	-3.80130	-1.24470
H	-2.25950	-4.26610	-1.21330
H	-3.97120	-4.57680	-1.58630
C	-5.05870	-2.67950	0.10140
H	-5.35550	-2.33390	1.10850
H	-5.79900	-3.43580	-0.21580
C	-2.65140	-2.24650	0.63550
H	-2.93080	-1.92140	1.65100
H	-1.63920	-2.68970	0.70110
C	-3.65660	-3.30420	0.15230
H	-3.64940	-4.14620	0.86500
Au	0.89580	-0.66750	-0.34760
C	1.78360	-1.74010	-2.10830
C	3.10860	-2.30020	-1.63580
H	1.80340	-1.08910	-2.98870
H	0.92600	-2.42740	-2.07250
C	3.70590	-0.04830	-0.51470
C	2.91090	-1.09020	-0.83620
H	3.26300	0.92700	-0.28190
C	7.98550	-0.05540	-0.26770
C	7.27230	1.14280	-0.32870
C	5.88080	1.12350	-0.40020
C	5.17580	-0.09220	-0.42320
C	5.90570	-1.29120	-0.34460
C	7.29660	-1.27060	-0.27010
H	9.07780	-0.04320	-0.20820
H	7.80290	2.09940	-0.31780
H	5.32480	2.06560	-0.44450
H	5.38360	-2.25080	-0.31650
H	7.84840	-2.21300	-0.20550
H	3.94710	-2.29180	-2.34640
H	3.00560	-3.26170	-1.11200

MeDalPhosAu(ACP) proximal prod

SCF = –2008.27903
ZPE = –2007.46625

N	-0.95270	0.06650	2.25880
P	1.35300	-0.01780	0.20890
C	-1.68740	1.28430	2.67300
C	-1.66060	-1.15670	2.70700
C	1.79510	0.15230	4.81540
C	2.93980	0.11870	4.01980
C	2.80940	0.05450	2.63830
C	1.54600	0.02260	2.02440
C	0.40000	0.07460	2.83130
C	0.53750	0.13250	4.22380
H	1.87800	0.19950	5.90460
H	3.93370	0.14370	4.47380
H	3.71070	0.04130	2.02460
H	-0.34580	0.16310	4.86550
H	-1.79430	-1.16170	3.80290
H	-2.64940	-1.20530	2.23230
H	-1.07730	-2.04090	2.40950
H	-1.80870	1.33100	3.76910
H	-1.13680	2.17350	2.33110
H	-2.68350	1.27980	2.21020
C	1.60720	-4.11060	-0.47590
C	1.60420	-4.06630	-2.01060
C	2.56390	-2.96870	-2.49110
C	2.10910	-1.60410	-1.95080
C	2.09210	-1.62520	-0.40540
C	1.13920	-2.75230	0.06950
H	0.91000	-4.88900	-0.12250
H	0.58240	-3.86790	-2.38260
H	1.91560	-5.04510	-2.41760
H	2.56180	-2.92720	-3.59340
H	2.80270	-0.82700	-2.31090
H	1.10670	-1.35960	-2.34210
H	0.11000	-2.55690	-0.28130
H	1.11100	-2.77880	1.17430
C	3.98310	-3.26070	-1.98410
H	4.67930	-2.47840	-2.33810
H	4.34030	-4.22450	-2.38930
C	3.02460	-4.41190	0.02890
H	3.03080	-4.46620	1.13270
H	3.36090	-5.39390	-0.34990
C	3.50780	-1.95340	0.11620
H	3.49620	-2.02170	1.21590
H	4.22440	-1.16540	-0.16400
C	3.97550	-3.30660	-0.44980
H	4.99490	-3.50510	-0.07830
C	4.24920	2.80420	-1.10950
C	3.64880	3.10240	-2.49020
C	2.12310	3.20760	-2.36560
C	1.54810	1.88530	-1.83390
C	2.16150	1.54610	-0.45310
C	3.69590	1.46500	-0.59270
H	5.34620	2.72060	-1.18950
H	3.91770	2.30040	-3.20170
H	4.06250	4.04600	-2.88910
H	1.67960	3.40660	-3.35570
H	0.45510	1.98860	-1.73510
H	1.73840	1.07600	-2.55750
H	3.97100	0.66520	-1.29950
H	4.16340	1.22760	0.37680
C	1.75740	4.33870	-1.39550
H	0.65870	4.43330	-1.32140
H	2.14470	5.30330	-1.77030
C	3.88440	3.92940	-0.13060
H	4.32670	3.72700	0.86190
H	4.30130	4.88930	-0.48470
C	1.79990	2.70000	0.51410
H	2.22060	2.51030	1.51510
H	0.70120	2.76880	0.62600
C	2.35620	4.03140	-0.01660
H	2.08730	4.82990	0.69520
Au	-1.03890	-0.00220	-0.02380
C	-1.38410	-0.23170	-2.04060
C	-2.86760	0.13980	-2.01940
H	-1.20640	-1.29590	-2.26110
H	-0.72530	0.40250	-2.64810
C	-4.14730	0.04230	0.22980
C	-3.03880	0.08390	-0.52700
H	-4.04440	-0.06640	1.31580
C	-8.28450	0.05660	-0.94470
C	-7.88810	-0.42220	0.30570
C	-6.54220	-0.40560	0.66450
C	-5.55660	0.07630	-0.21650
C	-5.97440	0.57050	-1.46470
C	-7.32250	0.55750	-1.82300
H	-9.34060	0.04710	-1.23000
H	-8.63310	-0.80910	1.00760
H	-6.23960	-0.77920	1.64850
H	-5.24850	0.99420	-2.16050
H	-7.62440	0.94940	-2.79910
H	-3.52220	-0.54330	-2.59300
H	-3.02860	1.16130	-2.40910

MeDalPhosAu(ACP) σ intermediate

SCF = –2008.26491
ZPE = –2007.45582

N	-0.12380	-1.07570	2.67040
P	1.47490	0.10770	0.17790
C	-1.16720	-0.38440	3.40950
C	-0.23050	-2.52250	2.76110
C	2.95700	-0.20340	4.58360
C	3.77720	0.32250	3.58690
C	3.30970	0.38980	2.27940
C	2.02380	-0.05810	1.92550
C	1.19680	-0.58990	2.94000
C	1.68380	-0.65240	4.25420
H	3.30850	-0.26520	5.61750
H	4.78190	0.68260	3.82370
H	3.96850	0.81280	1.52100
H	1.04200	-1.06800	5.03560
H	-0.12080	-2.90270	3.80060
H	-1.21870	-2.84120	2.38580
H	0.54290	-2.99570	2.13550
H	-1.14570	-0.58670	4.50230
H	-1.07860	0.70350	3.25370
H	-2.15310	-0.70660	3.03160
C	2.82050	-3.56930	-1.25300
C	2.50260	-3.34710	-2.73860
C	2.96990	-1.94560	-3.15550
C	2.23490	-0.88390	-2.32240
C	2.52670	-1.08970	-0.81920
C	2.08070	-2.51670	-0.41300
H	2.47660	-4.57040	-0.94210
H	1.41620	-3.45190	-2.91430
H	3.00810	-4.11400	-3.35270
H	2.73970	-1.77650	-4.22100
H	2.56990	0.11730	-2.63990
H	1.14690	-0.94360	-2.51040
H	0.99150	-2.63200	-0.56080
H	2.28680	-2.68090	0.66000
C	4.48140	-1.80960	-2.92210
H	4.82310	-0.80500	-3.23210
H	5.02960	-2.54530	-3.53780
C	4.33260	-3.44350	-1.02460
H	4.57280	-3.62470	0.03880
H	4.86990	-4.20640	-1.61650
C	4.04930	-0.99120	-0.57960
H	4.27680	-1.18510	0.48090
H	4.42320	0.01500	-0.82370
C	4.78720	-2.03690	-1.43560
H	5.87020	-1.92810	-1.25680
C	3.13650	3.86190	-1.01850
C	2.22560	4.13260	-2.22250
C	0.79100	3.71950	-1.86870
C	0.75190	2.22440	-1.51540
C	1.67640	1.92480	-0.30590
C	3.10910	2.36060	-0.68120
H	4.17440	4.14310	-1.26470
H	2.57940	3.56440	-3.10200
H	2.25750	5.20420	-2.49020
H	0.12830	3.88970	-2.73400
H	-0.28660	1.93980	-1.27100
H	1.05310	1.62670	-2.39100
H	3.46060	1.78910	-1.55530
H	3.81040	2.16770	0.14510
C	0.29730	4.53620	-0.66700
H	-0.74400	4.25810	-0.42100
H	0.29930	5.61350	-0.91240
C	2.65220	4.67460	0.19050
H	3.31370	4.49640	1.05770
H	2.69620	5.75460	-0.03820
C	1.18160	2.77260	0.89140
H	1.81890	2.60000	1.77410
H	0.15240	2.47290	1.16640
C	1.21230	4.26730	0.53510
H	0.86150	4.84220	1.40880
Au	-0.74690	-0.46300	-0.03530
C	-2.78750	-1.78380	-0.43670
C	-3.16730	-0.20140	-0.21910
C	-5.43080	-1.55930	-0.59310
C	-4.14380	-1.25640	-0.45520
H	-5.69030	-2.60830	-0.77710
C	-8.78040	1.11350	-0.40640
C	-8.96570	-0.25150	-0.62800
C	-7.86530	-1.10600	-0.68500
C	-6.56000	-0.61470	-0.52160
C	-6.38780	0.76260	-0.29790
C	-7.48700	1.61590	-0.24110
H	-9.64150	1.78670	-0.36200
H	-9.97420	-0.65500	-0.75800
H	-8.01650	-2.17600	-0.85940
H	-5.38370	1.17410	-0.16740
H	-7.33350	2.68500	-0.06640
H	-2.49910	-2.38730	0.43560
H	-2.35480	-2.09120	-1.39920
H	-2.99350	0.50310	-1.04490
H	-3.13380	0.22680	0.79310

MeDalPhosAu(ACP) distal TS

SCF = –2008.25987
ZPE = –2007.45190

N	-0.52960	-0.38820	2.62290
P	1.45470	0.00230	0.21270
C	-1.33890	0.66020	3.23030
C	-1.08380	-1.71610	2.85280
C	2.55700	-0.29760	4.72350
C	3.55240	-0.11670	3.76400
C	3.20240	-0.03540	2.42130
C	1.86510	-0.12740	1.99750
C	0.86210	-0.29930	2.97380
C	1.22850	-0.38690	4.32480
H	2.81480	-0.36790	5.78420
H	4.60260	-0.03860	4.05790
H	3.99480	0.11000	1.68730
H	0.45010	-0.52820	5.07960
H	-1.13150	-1.98080	3.93060
H	-2.10890	-1.75960	2.44640
H	-0.47310	-2.47130	2.33360
H	-1.40890	0.57250	4.33470
H	-0.91840	1.64820	2.98170
H	-2.36390	0.60600	2.82620
C	1.75370	-4.03150	-0.78260
C	1.58140	-3.88890	-2.30170
C	2.45170	-2.72920	-2.80610
C	2.01600	-1.42100	-2.12750
C	2.17030	-1.53780	-0.59420
C	1.31220	-2.72880	-0.09760
H	1.12170	-4.85560	-0.41040
H	0.52000	-3.70010	-2.54690
H	1.87110	-4.82810	-2.80660
H	2.32920	-2.61730	-3.89660
H	2.63760	-0.59480	-2.50990
H	0.96600	-1.19250	-2.38870
H	0.24400	-2.54470	-0.31510
H	1.41250	-2.83080	0.99800
C	3.92600	-3.00130	-2.47390
H	4.55690	-2.17320	-2.84550
H	4.26620	-3.92310	-2.97920
C	3.22580	-4.31370	-0.45400
H	3.35380	-4.43980	0.63640
H	3.54760	-5.25630	-0.93260
C	3.64370	-1.84820	-0.25350
H	3.75770	-1.98280	0.83410
H	4.30190	-1.01990	-0.55880
C	4.08620	-3.14560	-0.95440
H	5.14520	-3.33030	-0.70650
C	4.16080	2.99580	-1.19990
C	3.40910	3.37570	-2.48240
C	1.90340	3.41830	-2.18960
C	1.43060	2.03950	-1.70340
C	2.18800	1.62540	-0.41420
C	3.69930	1.60600	-0.72620
H	5.24450	2.95300	-1.40130
H	3.62070	2.63900	-3.27880
H	3.75310	4.36030	-2.84750
H	1.35010	3.67240	-3.10960
H	0.34610	2.08020	-1.50010
H	1.58270	1.29260	-2.49960
H	3.91300	0.86630	-1.51480
H	4.28270	1.31930	0.16280
C	1.61470	4.46310	-1.10310
H	0.52850	4.51300	-0.90380
H	1.93100	5.46540	-1.44400
C	3.87750	4.03600	-0.10680
H	4.42980	3.77800	0.81520
H	4.23080	5.03120	-0.43200
C	1.90470	2.69890	0.66490
H	2.43180	2.45090	1.60090
H	0.82250	2.72460	0.89570
C	2.36890	4.08070	0.17750
H	2.16120	4.82010	0.96950
Au	-0.89490	0.02260	-0.06570
C	-2.43480	0.09190	-1.73040
C	-3.08880	0.05140	0.03760
C	-5.04640	0.10710	-1.72280
C	-3.80500	0.08940	-1.23860
H	-5.15880	0.13000	-2.81310
C	-8.75750	0.08270	0.43260
C	-8.73700	0.07920	-0.96260
C	-7.52080	0.08750	-1.64380
C	-6.29970	0.09880	-0.94860
C	-6.33660	0.10370	0.45750
C	-7.55210	0.09540	1.13810
H	-9.71010	0.07650	0.97030
H	-9.67480	0.07010	-1.52600
H	-7.51280	0.08480	-2.73850
H	-5.40840	0.11680	1.03330
H	-7.55810	0.09990	2.23230
H	-2.07860	-0.82950	-2.21450
H	-2.06450	1.03020	-2.16820
H	-3.13840	0.97530	0.63750
H	-3.17080	-0.89110	0.60470

MeDalPhosAu(ACP) distal prod

SCF = –2008.28652
ZPE = –2007.47504

N	-0.40950	-0.39260	2.61790
P	1.39600	0.06310	0.14630
C	-1.14320	0.74180	3.22730
C	-0.93320	-1.67530	3.14030
C	2.79420	-0.15210	4.55950
C	3.73040	0.09160	3.55560
C	3.31240	0.15210	2.23180
C	1.96510	-0.03560	1.88100
C	1.02610	-0.26030	2.90080
C	1.45350	-0.32010	4.23270
H	3.10410	-0.20240	5.60680
H	4.78470	0.24000	3.80290
H	4.05020	0.36440	1.45660
H	0.73410	-0.48850	5.03680
H	-0.81470	-1.74290	4.23550
H	-2.00490	-1.74700	2.90490
H	-0.39900	-2.51140	2.66460
H	-1.05010	0.72940	4.32770
H	-0.73910	1.68880	2.84000
H	-2.20860	0.66780	2.96390
C	2.15130	-3.84380	-1.07260
C	1.78250	-3.67190	-2.55320
C	2.41070	-2.37760	-3.08880
C	1.87690	-1.17360	-2.29720
C	2.22650	-1.32600	-0.79910
C	1.60760	-2.64740	-0.27540
H	1.69240	-4.76560	-0.67690
H	0.68370	-3.63650	-2.66910
H	2.14430	-4.53770	-3.13640
H	2.14400	-2.24480	-4.15080
H	2.32750	-0.25110	-2.69920
H	0.78370	-1.09170	-2.42810
H	0.50800	-2.62110	-0.37400
H	1.84050	-2.77160	0.79810
C	3.93730	-2.43960	-2.93890
H	4.39400	-1.51320	-3.33270
H	4.34310	-3.28060	-3.52950
C	3.67700	-3.91690	-0.92670
H	3.95160	-4.06240	0.13390
H	4.06990	-4.78380	-1.48800
C	3.75820	-1.42860	-0.63850
H	4.01380	-1.59110	0.42090
H	4.25320	-0.50060	-0.96360
C	4.29570	-2.61670	-1.45690
H	5.39210	-2.65070	-1.34000
C	3.43420	3.46970	-1.38030
C	2.49070	3.77050	-2.55330
C	1.03730	3.57970	-2.09840
C	0.82240	2.13160	-1.63090
C	1.78450	1.79550	-0.46440
C	3.23860	2.01130	-0.93120
H	4.48150	3.59900	-1.70160
H	2.71510	3.09810	-3.40150
H	2.64360	4.80600	-2.90700
H	0.35250	3.78140	-2.93940
H	-0.22100	2.01300	-1.29290
H	0.97360	1.43930	-2.47530
H	3.47070	1.34230	-1.77560
H	3.94540	1.77850	-0.11780
C	0.72940	4.53450	-0.93710
H	-0.32260	4.41780	-0.61840
H	0.85910	5.58270	-1.26150
C	3.12890	4.41820	-0.21240
H	3.81400	4.21420	0.63070
H	3.29410	5.46570	-0.52230
C	1.47390	2.77270	0.69560
H	2.13630	2.57400	1.55430
H	0.43300	2.62700	1.04100
C	1.67220	4.22530	0.23350
H	1.44870	4.89500	1.08100
Au	-0.94970	-0.37460	0.41420
C	-1.81230	-0.52070	-1.46000
C	-2.98280	-0.81170	0.48520
C	-4.33500	-0.13670	-1.61640
C	-3.22400	-0.50600	-0.95990
H	-4.21800	0.16580	-2.66510
C	-8.37870	0.20080	-0.18850
C	-7.97160	0.82850	-1.36680
C	-6.65620	0.70070	-1.80870
C	-5.70830	-0.04740	-1.08630
C	-6.13960	-0.68540	0.09400
C	-7.45690	-0.55890	0.53400
H	-9.41100	0.29610	0.16100
H	-8.68460	1.42050	-1.94880
H	-6.34930	1.19390	-2.73690
H	-5.45250	-1.30600	0.67610
H	-7.76790	-1.06790	1.45150
H	-1.50000	-1.49970	-1.86430
H	-1.51490	0.29410	-2.13740
H	-3.53100	-0.18290	1.20700
H	-3.09340	-1.88140	0.74050

5.3. *Biphenylene vs benzocyclobutene oxidative addition*

5.3.1. *Potential energy surfaces*

Comparisons between the two 4-membered strained-ring substrates were investigated to aid explanation of why biphenylene readily undergoes oxidative addition, while benzocyclobutene does not.

Figure S31: Calculated potential energy surfaces for the oxidative addition of (left) biphenylene and (right) benzocyclobutenone to MeDalPhosAu$^+$.

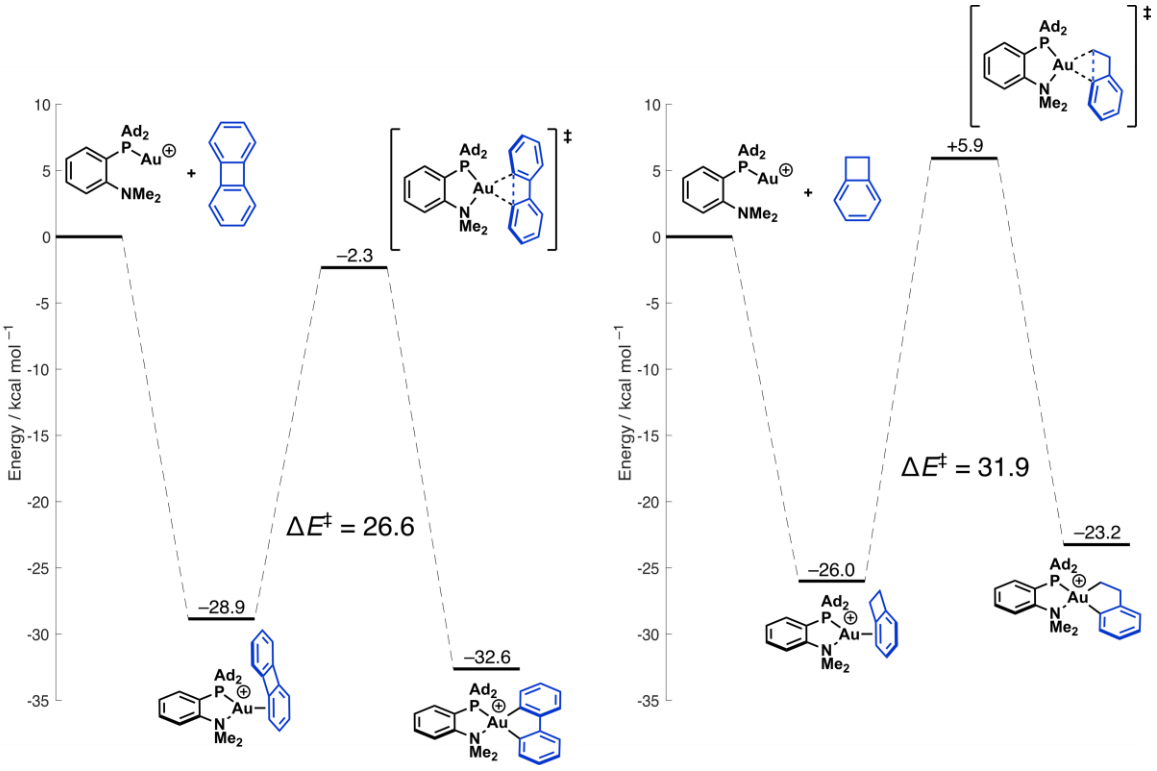

Compared to biphenylene, the oxidative addition of benzocyclobutene (**2f**) proceeds with a higher barrier ($\Delta E^{\ddagger}$ = 31.9 *vs* 26.6 kcal mol–1) and is more exothermic (ΔE = 2.8 *vs* –3.7 kcal mol^{-1}). The fact that oxidative addition of biphenylene generates two Au–C(sp^2) bonds, compared to the one in benzocyclobutene {and one Au–C(sp^3)}, may provide an explanation for these findings. A Au–C(sp^2) bond will be stronger than a Au–C(sp^3) bond so having two formed in the biphenylene example may cause a lowering of the transition state and product energies.

5.3.2. Energies (au) and Cartesian coordinates (Å) for stationary points

Biphenylene

SCF = –461.54079
ZPE = –461.38052

C	1.91360	-1.44510	0.00010
C	1.91360	1.44510	-0.00000
C	3.11910	0.69490	0.00000
C	3.11910	-0.69490	0.00000
H	1.93260	-2.53800	0.00010
H	1.93260	2.53800	-0.00000
H	4.07520	1.22710	0.00000
H	4.07520	-1.22710	0.00000
C	0.75440	-0.70960	0.00000
C	0.75440	0.70960	-0.00000
C	-0.75440	0.70960	0.00000
C	-0.75440	-0.70960	-0.00000
C	-1.91360	-1.44510	-0.00000
C	-3.11910	-0.69490	0.00000
C	-3.11910	0.69490	0.00000
C	-1.91360	1.44510	0.00000
H	-1.93260	-2.53800	-0.00000
H	-4.07520	-1.22710	0.00000
H	-4.07520	1.22710	0.00000
H	-1.93260	2.53800	0.00010

MeDalPhosAu(biphenylene) η^2 complex

SCF = –2083.22025
ZPE = –2082.41940

C	2.61540	-0.45290	-0.71450
C	3.86370	-0.21520	-0.11340
H	3.91490	0.29310	0.85020
C	5.05140	-0.61870	-0.71270
H	6.00550	-0.42080	-0.21690
C	5.01200	-1.27220	-1.94360
H	5.93700	-1.59160	-2.43210
C	3.78660	-1.52080	-2.55140
H	3.76290	-2.03480	-3.51610
C	2.58090	-1.12780	-1.95370
C	1.13540	-2.85610	-2.83440
H	1.87200	-3.30320	-3.53380
H	0.12930	-3.01920	-3.25400
H	1.19990	-3.38360	-1.86890
C	1.18590	-0.65710	-3.86130
H	1.30840	0.41640	-3.64740
H	0.17400	-0.81700	-4.26980
H	1.92160	-0.94840	-4.64000
C	1.08150	1.95420	0.13810
C	2.37700	2.60660	0.66500
H	3.23390	2.29810	0.04450
H	2.58310	2.29110	1.70010
C	2.25620	4.14030	0.60410
H	3.18670	4.57880	1.00270
C	1.06060	4.60890	1.44550
H	1.20010	4.31600	2.50220
H	0.98890	5.71110	1.41850
C	-0.22640	3.98110	0.89230
H	-1.09160	4.30340	1.49630
C	-0.11620	2.45110	0.97840
H	0.01620	2.16240	2.03370
H	-1.05320	1.98530	0.62140
C	-0.42590	4.40610	-0.56860
H	-0.53120	5.50410	-0.63310
H	-1.35710	3.96350	-0.96550
C	0.77600	3.94140	-1.40340
H	0.63470	4.23330	-2.45780
C	2.06130	4.57580	-0.85460
H	2.92940	4.26360	-1.46300
H	1.99670	5.67710	-0.91660
C	0.88240	2.41020	-1.32920
H	-0.03640	1.95660	-1.74460
H	1.72880	2.06230	-1.94860
C	1.02250	-0.73050	1.81660
C	1.88950	-0.06140	2.90260
H	1.55980	0.97810	3.06320
H	2.94760	-0.03180	2.59660
C	1.77150	-0.84040	4.22500
H	2.40960	-0.34760	4.97790
C	2.23960	-2.28770	4.01650
H	2.18290	-2.84420	4.96940
H	3.29600	-2.30180	3.69180
C	1.35490	-2.96400	2.95870
H	1.69320	-4.00150	2.79760
C	1.47950	-2.19810	1.63200
H	0.86410	-2.69030	0.85470
H	2.52760	-2.23360	1.29060
C	0.31140	-0.83320	4.69730
H	-0.02860	0.20540	4.86270
H	0.22110	-1.36570	5.66130
C	-0.56490	-1.51110	3.63580
H	-1.62130	-1.49390	3.95440
C	-0.10600	-2.96040	3.42880
H	-0.20290	-3.53000	4.37080
H	-0.74770	-3.45600	2.67710

C	-0.44840	-0.75050	2.30730
H	-0.83040	0.27490	2.43400
H	-1.08510	-1.24210	1.55170
C	-3.49760	0.73370	-0.62900
C	-3.58170	2.07310	-0.92410
H	-3.25510	2.50000	-1.87530
C	-4.11410	2.88700	0.10260
H	-4.20320	3.96410	-0.06570
C	-4.51890	2.35790	1.32720
H	-4.91610	3.03430	2.08980
C	-4.43450	0.97510	1.61360
H	-4.75890	0.57870	2.57870
C	-3.92200	0.18970	0.60590
C	-3.53020	-1.18730	0.15500
C	-3.58040	-2.52850	0.38120
H	-3.98640	-2.96630	1.29620
C	-3.09970	-3.37030	-0.67790
H	-3.11270	-4.45390	-0.53230
C	-2.66420	-2.86450	-1.88140
H	-2.35210	-3.54910	-2.67420
C	-2.62410	-1.44140	-2.14890
H	-2.50890	-1.07540	-3.17440
C	-3.03490	-0.62940	-1.08190
N	1.34130	-1.42480	-2.62840
P	1.07060	0.07880	0.11680
Au	-0.73450	-0.71060	-1.15170

MeDalPhosAu(biphenylene) TS

SCF = –2083.17766
ZPE = –2082.37714

C	1.46930	-0.98440	1.88280
C	2.70720	-0.80610	2.52490
H	3.45340	-0.13990	2.09050
C	3.01010	-1.45200	3.71790
H	3.98080	-1.29010	4.19400
C	2.06850	-2.30190	4.29720
H	2.29100	-2.82030	5.23430
C	0.83970	-2.49130	3.67610
H	0.10650	-3.16250	4.13090
C	0.52130	-1.84350	2.47410
C	-1.88000	-1.68120	2.72550
H	-1.97190	-2.30700	3.63730
H	-2.82070	-1.76860	2.15620
H	-1.75510	-0.62990	3.03090
C	-0.90490	-3.46120	1.40820
H	-0.07560	-3.71350	0.72890
H	-1.85400	-3.56410	0.85840
H	-0.91370	-4.19240	2.24370
C	2.20920	-0.81200	-0.98470
C	3.70480	-0.90190	-0.61480
H	3.83240	-1.52190	0.28740
H	4.11740	0.09510	-0.39560
C	4.49390	-1.54560	-1.76950
H	5.55960	-1.57960	-1.48630
C	4.32370	-0.71400	-3.04870
H	4.71130	0.30930	-2.89220
H	4.90910	-1.16250	-3.87140
C	2.83660	-0.65990	-3.42710
H	2.70510	-0.05820	-4.34230
C	2.05140	0.00540	-2.28600
H	2.42760	1.03120	-2.13870
H	0.98270	0.08140	-2.55900
C	2.30640	-2.08160	-3.66000
H	2.84830	-2.55590	-4.49790
H	1.23740	-2.04590	-3.94000
C	2.48750	-2.90940	-2.37950
H	2.09840	-3.92980	-2.53500
C	3.97540	-2.97000	-2.00860
H	4.11530	-3.58220	-1.09910
H	4.54900	-3.45290	-2.82020
C	1.69740	-2.25260	-1.23750
H	0.62280	-2.23330	-1.50000
H	1.80090	-2.85270	-0.31570
C	1.36160	1.75440	0.67220
C	2.82810	2.23260	0.63970
H	3.26110	2.05800	-0.35870
H	3.44080	1.67800	1.36750
C	2.90040	3.73610	0.96150
H	3.95950	4.04450	0.94770
C	2.30180	4.00020	2.35080
H	2.37650	5.07490	2.59660
H	2.87150	3.44840	3.12080
C	0.83110	3.55830	2.36660
H	0.39830	3.73650	3.36550
C	0.75470	2.05310	2.06470
H	-0.29940	1.71700	2.09540
H	1.29780	1.50020	2.84890
C	2.11650	4.52240	-0.09760
H	2.55160	4.34780	-1.09860
H	2.18550	5.60640	0.10480
C	0.64970	4.07360	-0.07360
H	0.07750	4.61640	-0.84510
C	0.04440	4.34560	1.30980
H	0.08040	5.42640	1.53730
H	-1.01880	4.04280	1.32260
C	0.55810	2.56980	-0.37200
H	0.92420	2.36740	-1.39190
H	-0.50010	2.26640	-0.34260
C	-2.54660	0.73550	-1.44110

C	-1.96690	1.36070	-2.52850
H	-1.18280	0.89320	-3.12860
C	-2.38290	2.68210	-2.79810
H	-1.90340	3.22890	-3.61520
C	-3.36850	3.30300	-2.03850
H	-3.66060	4.33070	-2.27190
C	-4.04000	2.60700	-1.01200
H	-4.89550	3.05420	-0.49910
C	-3.62160	1.32220	-0.73350
C	-4.17360	0.10390	-0.09910
C	-5.35670	-0.30120	0.47960
H	-6.09530	0.41810	0.84270
C	-5.61730	-1.68850	0.52350
H	-6.53950	-2.04490	0.99120
C	-4.75850	-2.60380	-0.07370
H	-5.01110	-3.66790	-0.06900
C	-3.55760	-2.18690	-0.69040
H	-2.90060	-2.91320	-1.17530
C	-3.25020	-0.83980	-0.61210
N	-0.76490	-2.08450	1.87130
P	1.09220	-0.08260	0.33260
Au	-1.17910	-0.47110	-0.24250

MeDalPhosAu(biphenylene) prod

SCF = –2083.23068
ZPE = –2082.42542

C	1.44480	1.40400	1.53530
C	2.73830	1.90980	1.76500
H	3.57160	1.58400	1.14760
C	2.99810	2.81520	2.78340
H	4.01460	3.18810	2.93190
C	1.95850	3.23130	3.61370
H	2.14340	3.93710	4.42790
C	0.67550	2.74730	3.40260
H	-0.12610	3.08870	4.05990
C	0.41020	1.84590	2.36170
C	-1.78450	2.63980	1.90420
H	-1.87850	3.25170	2.81640
H	-2.78750	2.38270	1.55080
H	-1.28100	3.22510	1.12130
C	-1.39750	0.68160	3.38950
H	-0.65280	-0.10040	3.59890
H	-2.37040	0.19920	3.24740
H	-1.46260	1.36490	4.25260
C	2.10640	-1.33790	0.53980
C	3.34240	-1.06200	1.42860
H	3.04400	-0.55660	2.36020
H	4.05140	-0.40550	0.89730
C	4.05910	-2.37530	1.79070
H	4.92430	-2.12560	2.42800
C	4.53670	-3.07260	0.51210
H	5.25500	-2.42910	-0.02750
H	5.06160	-4.01200	0.76240
C	3.32090	-3.36350	-0.37450
H	3.64720	-3.84760	-1.31030
C	2.62110	-2.04260	-0.73810
H	3.33000	-1.38810	-1.26910
H	1.80910	-2.26570	-1.44160
C	2.34150	-4.28180	0.37090
H	2.83080	-5.24490	0.60160
H	1.46700	-4.50380	-0.26780
C	1.88440	-3.59870	1.66810
H	1.17870	-4.25350	2.20640
C	3.09880	-3.29560	2.55440
H	2.77190	-2.81110	3.49250
H	3.61340	-4.23280	2.83220
C	1.16240	-2.28860	1.31910
H	0.26650	-2.51920	0.72030
H	0.81710	-1.79330	2.24590
C	1.50300	1.18170	-1.39200
C	3.00750	1.47920	-1.57170
H	3.58740	0.54320	-1.61990
H	3.38790	2.06480	-0.72290
C	3.23250	2.30000	-2.85440
H	4.31360	2.49370	-2.95570
C	2.47600	3.63160	-2.74170
H	2.65090	4.24430	-3.64420
H	2.85110	4.20820	-1.87660
C	0.97430	3.35350	-2.58080
H	0.42600	4.30570	-2.48390
C	0.74110	2.52910	-1.30330
H	-0.34160	2.34500	-1.17180
H	1.08530	3.10860	-0.42970
C	2.72670	1.51750	-4.07240
H	3.27440	0.56160	-4.16430
H	2.91370	2.09250	-4.99710
C	1.22520	1.25020	-3.90870
H	0.85050	0.67200	-4.77000
C	0.46380	2.57770	-3.80230
H	0.61090	3.17680	-4.71890
H	-0.62030	2.38460	-3.70680
C	0.98500	0.42510	-2.63530
H	1.48560	-0.55060	-2.73390
H	-0.09430	0.23440	-2.53240
C	-1.82150	-1.29390	-1.09270
C	-0.90640	-2.08020	-1.78420
H	0.14210	-2.08030	-1.50970
C	-1.30800	-2.87940	-2.85810
H	-0.56400	-3.48240	-3.38670

C	-2.64320	-2.89430	-3.25160
H	-2.96410	-3.50580	-4.09960
C	-3.57800	-2.14260	-2.54150
H	-4.63140	-2.18630	-2.83060
C	-3.18240	-1.35950	-1.45230
C	-4.09670	-0.65430	-0.54540
C	-5.48840	-0.64830	-0.67730
H	-5.96150	-1.11810	-1.54400
C	-6.28660	-0.06150	0.30240
H	-7.37510	-0.06000	0.19580
C	-5.68550	0.50060	1.42410
H	-6.29620	0.94190	2.21740
C	-4.29100	0.50800	1.54870
H	-3.88990	0.96170	2.45440
C	-3.46830	-0.04220	0.56170
N	-0.97940	1.41250	2.16280
P	1.07180	0.22720	0.17850
Au	-1.36550	-0.01150	0.43340

Benzocyclobutene (BCB)

SCF = –309.30186
ZPE = –309.16613

C	0.71800	-1.43900	0.00020
C	1.91300	-0.70110	0.00010
C	1.91300	0.70110	-0.00010
C	0.71800	1.43900	-0.00020
H	0.73440	-2.53320	0.00010
H	2.87130	-1.23030	-0.00000
H	2.87130	1.23030	0.00000
H	0.73440	2.53320	-0.00010
C	-0.45530	-0.69720	-0.00000
C	-0.45530	0.69720	0.00000
C	-1.97100	0.78540	0.00030
C	-1.97100	-0.78540	-0.00030
H	-2.41670	1.24610	0.89780
H	-2.41710	1.24700	-0.89650
H	-2.41710	-1.24700	0.89650
H	-2.41670	-1.24610	-0.89780

MeDalPhosAu(BCB) η^2 complex

SCF = –1930.97668

ZPE = –1930.20046

C	1.70470	0.62020	1.71890
C	2.90370	1.35320	1.67300
H	3.28050	1.72020	0.71750
C	3.62950	1.64090	2.82350
H	4.55730	2.21470	2.75170
C	3.16500	1.19240	4.05940
H	3.72560	1.40720	4.97380
C	1.97960	0.46960	4.12530
H	1.61600	0.11800	5.09490
C	1.23510	0.17450	2.97380
C	-0.98740	0.09920	3.91750
H	-0.70700	0.17960	4.99000
H	-1.93490	-0.46320	3.86000
H	-1.16470	1.11430	3.52530
C	0.23520	-1.95560	3.50140
H	0.95810	-2.42960	2.81840
H	-0.71660	-2.50990	3.43340
H	0.61810	-2.05560	4.54060
C	1.83270	-0.89480	-0.83390
C	3.30150	-0.47160	-1.04920
H	3.80560	-0.35190	-0.07660
H	3.36050	0.49250	-1.57730
C	4.04610	-1.54900	-1.85910
H	5.08520	-1.21200	-2.01310
C	3.36070	-1.75270	-3.21750
H	3.37720	-0.81160	-3.79700
H	3.90780	-2.50990	-3.80770
C	1.91120	-2.20590	-2.99270
H	1.40840	-2.34740	-3.96440
C	1.16150	-1.11900	-2.20750
H	1.16860	-0.18320	-2.79060
H	0.10420	-1.41320	-2.06970
C	1.89480	-3.52220	-2.20290
H	2.41060	-4.31380	-2.77540
H	0.85270	-3.85770	-2.04760
C	2.58870	-3.31310	-0.84930
H	2.57110	-4.25180	-0.27030
C	4.04010	-2.86880	-1.07650
H	4.55350	-2.73700	-0.10680
H	4.59090	-3.64590	-1.63660
C	1.83620	-2.23440	-0.05540
H	0.79890	-2.56950	0.12900
H	2.31390	-2.09160	0.93040
C	0.44960	1.95270	-0.66860
C	1.62350	2.54370	-1.47730
H	1.89980	1.85940	-2.29620
H	2.51380	2.67610	-0.84190
C	1.22300	3.90530	-2.07300
H	2.08560	4.30840	-2.63000
C	0.83600	4.87210	-0.94470
H	0.57430	5.85950	-1.36600
H	1.69370	5.02430	-0.26430
C	-0.35770	4.29850	-0.16630
H	-0.63410	4.98510	0.65170
C	0.04240	2.94710	0.44600

H	-0.80190	2.53300	1.02990
H	0.87920	3.10180	1.14690
C	0.03300	3.71900	-3.02350
H	0.30870	3.03660	-3.84830
H	-0.24530	4.68670	-3.47830
C	-1.15230	3.14360	-2.23770
H	-2.00900	2.98390	-2.91440
C	-1.55010	4.10930	-1.11360
H	-1.85690	5.08260	-1.53710
H	-2.41640	3.70540	-0.55790
C	-0.76170	1.79080	-1.62470
H	-0.53000	1.07090	-2.42650
H	-1.62520	1.38650	-1.06790
C	-2.79760	-3.04550	-1.29990
C	-3.21360	-2.13410	-2.50720
C	-3.63860	-1.10210	-1.48090
C	-4.28710	0.10260	-1.30240
H	-4.66610	0.70380	-2.13340
C	-4.45380	0.53700	0.03130
H	-4.95100	1.49170	0.22490
C	-4.02770	-0.22820	1.11510
H	-4.21620	0.13020	2.13080
C	-3.38790	-1.49400	0.93980
H	-3.24580	-2.17020	1.79000
C	-3.19550	-1.88600	-0.39880
N	0.01710	-0.57220	3.10670
P	0.78090	0.29280	0.16760
Au	-1.27720	-0.69440	0.60820
H	-4.02130	-2.53480	-3.14010
H	-2.37350	-1.82150	-3.14900
H	-1.74050	-3.35100	-1.26930
H	-3.43380	-3.93610	-1.17250

MeDalPhosAu(BCB) TS

SCF = –1930.92539
ZPE = –1930.14960

C	0.92880	-0.32380	2.09190
C	2.15000	-0.24070	2.78490
H	3.07280	-0.03780	2.24020
C	2.22070	-0.40020	4.16400
H	3.18640	-0.32660	4.67110
C	1.05550	-0.65090	4.88770
H	1.09310	-0.77870	5.97340
C	-0.15980	-0.74180	4.22010
H	-1.07060	-0.94680	4.78930
C	-0.24420	-0.58570	2.82870
C	-2.49800	0.27550	2.63120
H	-2.80250	0.14500	3.69130
H	-3.40400	0.19490	2.00680
H	-2.08200	1.28910	2.50870
C	-2.04780	-2.07290	2.27270
H	-1.30910	-2.78500	1.87220
H	-2.96650	-2.14460	1.66850
H	-2.29910	-2.37280	3.31250
C	1.74520	-1.51740	-0.49470
C	3.13150	-1.86010	0.08990
H	3.04330	-2.07100	1.16810
H	3.82910	-1.01690	-0.02920
C	3.70560	-3.10560	-0.60970
H	4.70160	-3.31520	-0.18460
C	3.82870	-2.84820	-2.11810
H	4.51260	-2.00010	-2.30480
H	4.26190	-3.73260	-2.61920
C	2.43980	-2.54220	-2.69690
H	2.52090	-2.34990	-3.78010
C	1.87420	-1.28560	-2.01670
H	2.54430	-0.43280	-2.21570
H	0.88650	-1.03670	-2.44740
C	1.50160	-3.73210	-2.44970
H	1.89070	-4.63310	-2.95710
H	0.50330	-3.52260	-2.87590
C	1.38850	-3.98700	-0.93970
H	0.70460	-4.83220	-0.75310
C	2.77600	-4.30170	-0.36460
H	2.70090	-4.50760	0.71860
H	3.18810	-5.20870	-0.84270
C	0.81770	-2.73570	-0.25550
H	-0.19280	-2.52840	-0.65480
H	0.71140	-2.91720	0.82910
C	1.71150	1.62180	-0.04370
C	3.25430	1.60750	-0.07530
H	3.61050	0.94390	-0.88010
H	3.66550	1.22770	0.87310
C	3.79250	3.02860	-0.32040
H	4.89500	2.98770	-0.31990
C	3.30820	3.96620	0.79500
H	3.71590	4.98140	0.64040
H	3.67630	3.61170	1.77510
C	1.77320	4.01020	0.79510
H	1.41930	4.67630	1.60010
C	1.22930	2.59660	1.05840
H	0.12260	2.61590	1.08410
H	1.57270	2.25930	2.04970
C	3.29240	3.53640	-1.67910
H	3.64980	2.87170	-2.48660
H	3.69620	4.54460	-1.88210
C	1.75900	3.57560	-1.66700
H	1.38450	3.91890	-2.64650
C	1.27200	4.52200	-0.56190

H	1.64600	5.54580	-0.74330
H	0.16740	4.57100	-0.56470
C	1.20260	2.16840	-1.40160
H	1.48350	1.49180	-2.22580
H	0.10160	2.22190	-1.38190
C	-2.43440	0.97540	-2.29930
C	-3.37200	2.07020	-1.78340
C	-4.21050	1.13230	-0.97000
C	-5.53190	1.11670	-0.54370
H	-6.15330	2.01560	-0.58990
C	-6.06620	-0.10090	-0.10470
H	-7.10180	-0.14190	0.24530
C	-5.31720	-1.28050	-0.16770
H	-5.77340	-2.22770	0.13510
C	-3.99320	-1.27300	-0.62140
H	-3.42890	-2.20700	-0.69170
C	-3.42860	-0.03350	-0.94100
N	-1.52490	-0.71620	2.18990
P	0.87800	-0.04460	0.27810
Au	-1.36930	0.01350	-0.49050
H	-2.84630	2.83830	-1.19200
H	-1.40770	1.27050	-2.56710
H	-2.85830	0.39210	-3.12640
H	-3.91620	2.57140	-2.60150

MeDalPhosAu(BCB) prod

SCF = –1930.97675
ZPE = –1930.19608

C	1.06510	-0.05650	2.04430
C	2.31590	0.04100	2.67900
H	3.22560	0.11690	2.08410
C	2.42600	0.05560	4.06320
H	3.41070	0.13480	4.53080
C	1.27380	-0.02920	4.84360
H	1.34010	-0.02210	5.93490
C	0.02950	-0.12250	4.23290
H	-0.85940	-0.19340	4.86260
C	-0.08720	-0.13060	2.83680
C	-2.29650	0.84360	2.75700
H	-2.45340	0.76170	3.84590
H	-3.27450	0.79690	2.26150
H	-1.82740	1.81220	2.52830
C	-1.96560	-1.58180	2.59630
H	-1.26360	-2.35260	2.24540
H	-2.93450	-1.73780	2.10710
H	-2.10040	-1.68300	3.68760
C	1.76100	-1.53430	-0.44760
C	3.15160	-1.81350	0.16290
H	3.06500	-1.94190	1.25320
H	3.83870	-0.97280	-0.02440
C	3.74010	-3.10600	-0.43020
H	4.73660	-3.26690	0.01430
C	3.86520	-2.97550	-1.95380
H	4.54110	-2.13930	-2.21000
H	4.30760	-3.89490	-2.37790
C	2.47480	-2.73140	-2.55500
H	2.55330	-2.62710	-3.65020
C	1.89580	-1.42740	-1.98300
H	2.55750	-0.58660	-2.24970
H	0.91290	-1.23390	-2.44000
C	1.54490	-3.90400	-2.21060
H	1.94160	-4.84080	-2.64130
H	0.54560	-3.73830	-2.65320
C	1.43150	-4.03430	-0.68470
H	0.75650	-4.86830	-0.42900
C	2.82130	-4.28550	-0.08460
H	2.74740	-4.40000	1.01220
H	3.24220	-5.22560	-0.48430
C	0.84280	-2.73630	-0.10930
H	-0.16470	-2.57660	-0.53270
H	0.72950	-2.82620	0.98660
C	1.62370	1.64570	-0.31140
C	3.16630	1.66890	-0.37010
H	3.53010	0.93350	-1.10580
H	3.59900	1.40320	0.60700
C	3.65840	3.07050	-0.76970
H	4.76120	3.05790	-0.79220
C	3.17180	4.10200	0.25790
H	3.54540	5.10690	-0.00860
H	3.57220	3.85830	1.25890
C	1.63670	4.10420	0.29080
H	1.28010	4.83560	1.03550
C	1.13770	2.70990	0.70380
H	0.03220	2.70570	0.75320
H	1.51370	2.48080	1.71390
C	3.11430	3.42470	-2.15970
H	3.47270	2.69110	-2.90480
H	3.48550	4.41740	-2.47210
C	1.58100	3.42640	-2.11450
H	1.17750	3.66270	-3.11350
C	1.09250	4.46580	-1.09720
H	1.43600	5.47480	-1.38810
H	-0.01250	4.48800	-1.07840
C	1.06770	2.03770	-1.70140
H	1.35850	1.29460	-2.46180
H	-0.03360	2.06810	-1.66500
C	-1.53520	-0.17400	-2.11170
C	-2.70090	0.67280	-2.59830
C	-3.87360	0.36410	-1.71550

C	-5.20400	0.48990	-2.12360
H	-5.43050	0.84670	-3.13390
C	-6.24070	0.14930	-1.25490
H	-7.28220	0.24510	-1.57680
C	-5.93970	-0.32630	0.02030
H	-6.74400	-0.60930	0.70640
C	-4.60800	-0.44940	0.42960
H	-4.42610	-0.83160	1.43680
C	-3.55130	-0.09660	-0.41970
N	-1.42760	-0.24430	2.24780
P	0.89490	-0.00840	0.22450
Au	-1.48780	-0.13220	-0.04760
H	-2.44540	1.74680	-2.53240
H	-0.57850	0.14530	-2.53820
H	-1.69050	-1.24060	-2.34940
H	-2.91130	0.46380	-3.66300

5.4. *Carbonylation potential energy surfaces*

5.4.1. *MeDalPhos vs IPr*

Figure S32: CO migratory insertion (MI) for MeDalPhos.

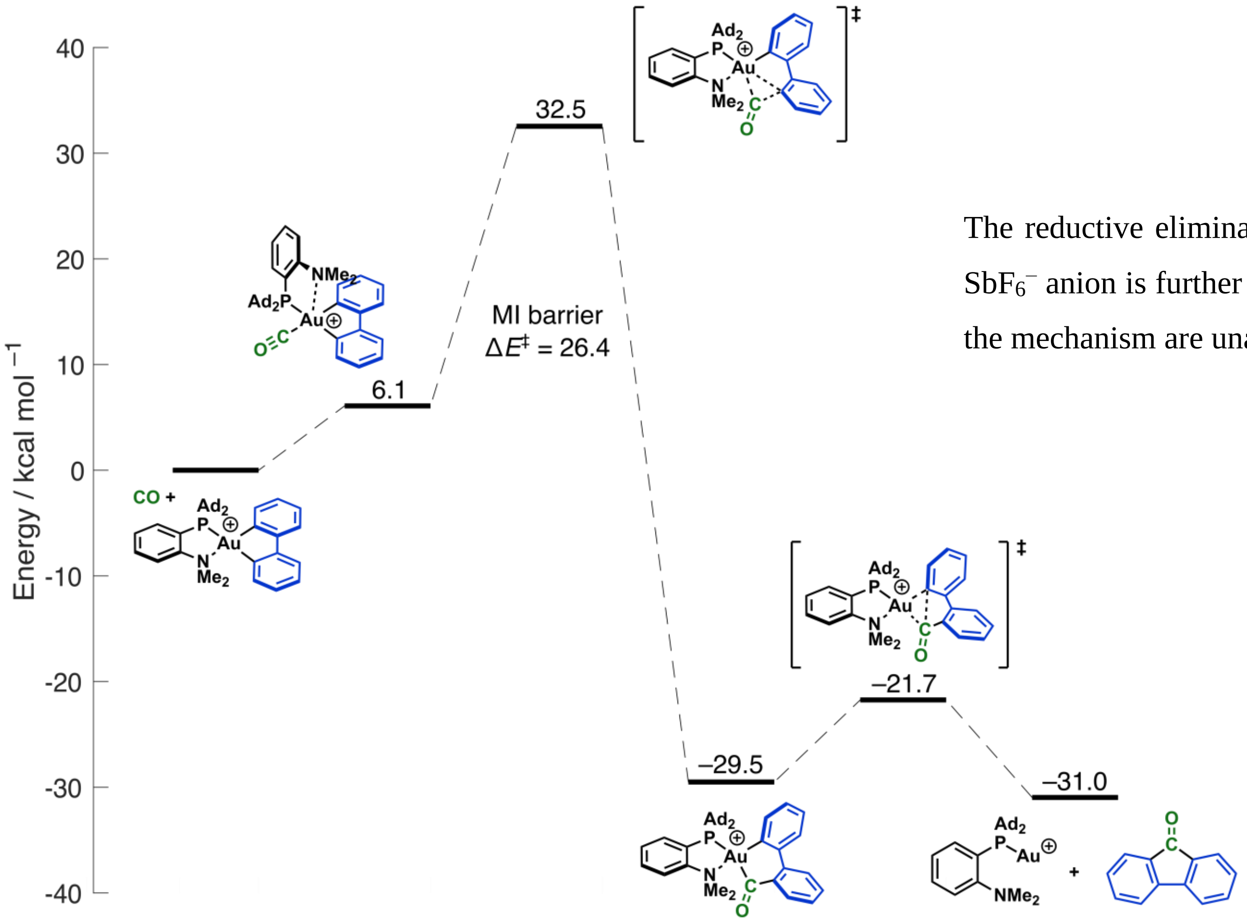

The reductive elimination forming 9-fluorenone including the SbF_6^- anion is further downhill at 29 kcal mol^{-1}. Other parts of the mechanism are unaffected by inclusion of the SbF_6^- anion.

Figure S33: CO migratory insertion (MI) for IPr.

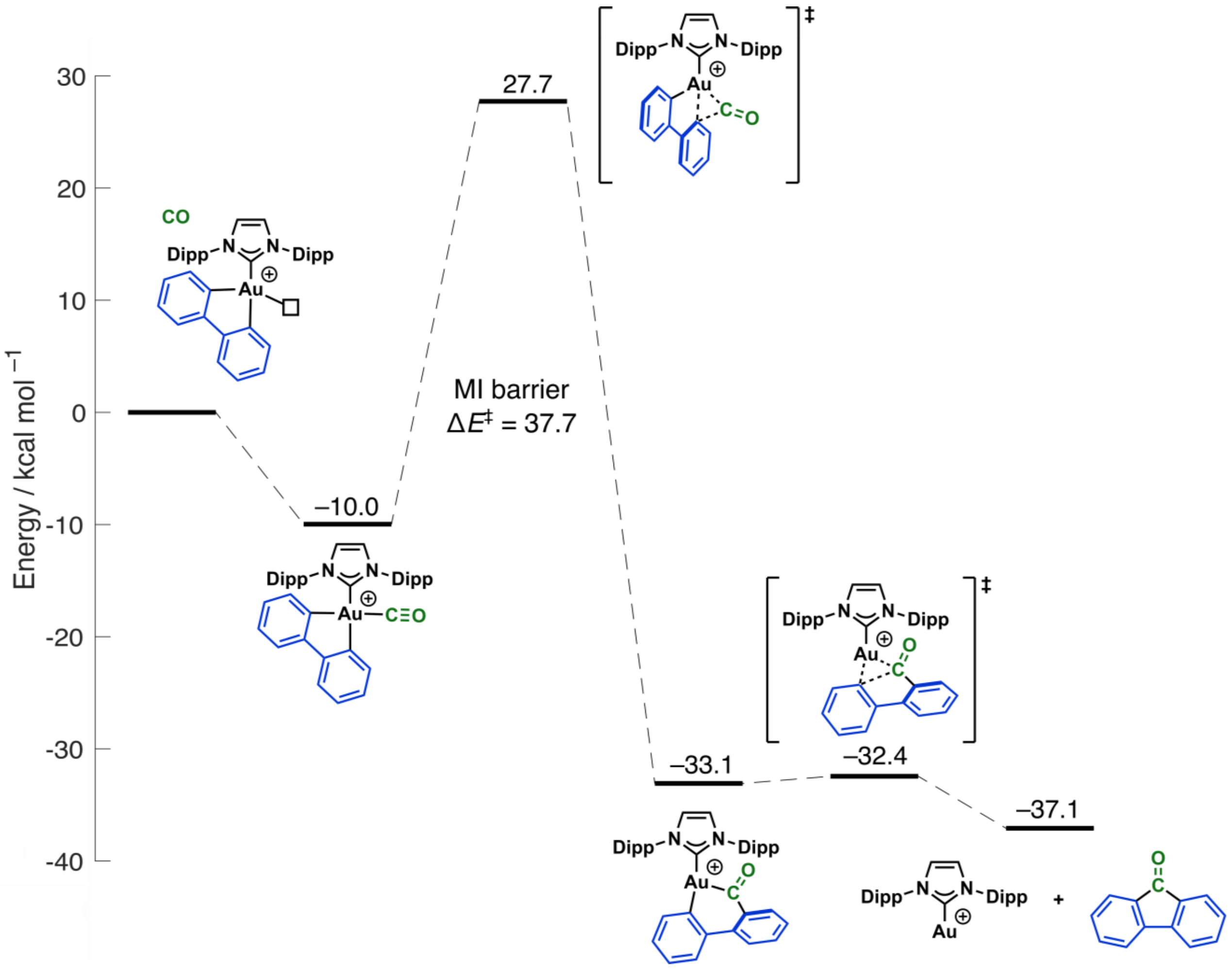

While both carbonylations with MeDalPhos- (Figure S32) and IPr- (Figure S33) ligated Au(III) complexes are both thermodynamically favorable, there are differences in their migratory insertion barriers. The barrier for the IPr complex is higher ($\Delta E^{\ddagger}$ = 37.7 kcal mol^{-1}) than that of the MeDalPhos complex ($\Delta E^{\ddagger}$ = 26.4 kcal mol^{-1}), the latter reactivity due to the change in coordination aided by the hemi-lability of the NMe_2 unit (see Scheme 4 in the manuscript). This was also further exemplified by a gas phase coordinate scan of the Au···C≡O distance (Figure S34). Upon decreasing the distance of the CO ligand to the Au center, there is a clear change in coordination such that the CO moves into the square plane w.r.t. to Au and the NMe_2 arm is at the apex of the distored square-based pyramid. The stability of the resultant intermediate was highlighted by a dihedral scan of the Au–P–C–C axis (Figure S35) where the NMe_2 group is moved away from the Au center at 15° increments. Increasing the dihedral angle gave, in general, progressively higher energy species.

Figure S34: Gas phase coordinate scan of the Au···C≡O distance *vs* the Au–N distance highlighting the hemi-lability of the NMe_2 group.

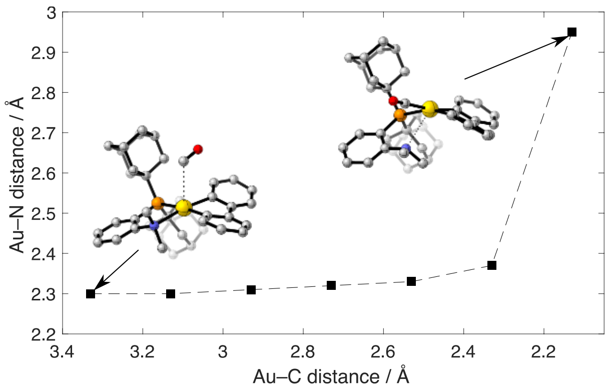

Figure S35: Gas phase dihedral scan of the Au–P–C–C axis (depicted below).

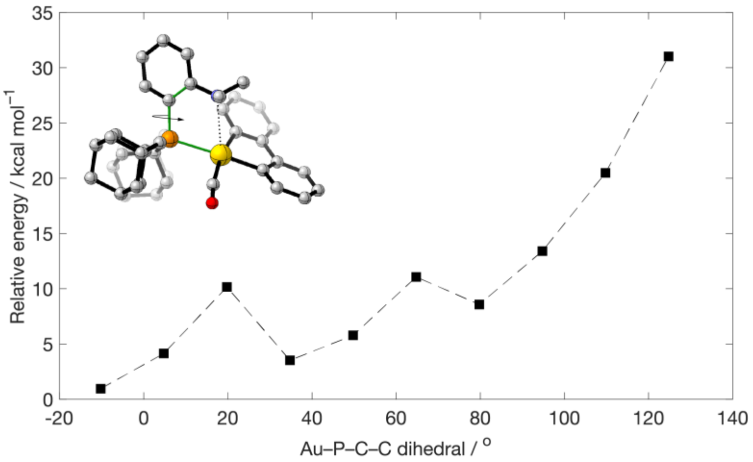

5.4.2. Energies (au) and Cartesian coordinates (Å) for stationary points

MeDalPhos(biphenylene)(CO) axial (Int1)

SCF = –2196.40255
ZPE = -2195.60748

N	-0.86440	-1.47980	2.14950
C	-2.76820	2.93180	-3.06910
C	-3.62360	2.41330	-2.09750
C	-3.18330	1.41840	-1.22010
C	-1.84610	0.96800	-1.27860
C	-1.02680	1.44370	-2.29570
C	-1.47890	2.42330	-3.18710
H	-3.12320	3.70910	-3.75170
H	-4.65550	2.77060	-2.04630
H	-0.02090	1.06250	-2.42390
H	-0.80640	2.78530	-3.97020
C	-6.19470	-0.09200	0.52380
C	-5.57000	-1.18520	1.11710
C	-4.18060	-1.33470	1.03330
C	-3.39310	-0.40070	0.35700
C	-4.04900	0.65440	-0.31240
C	-5.43300	0.82050	-0.20380
H	-7.27780	0.03840	0.60310
H	-6.15830	-1.93380	1.65610
H	-3.75110	-2.20950	1.52170
H	-5.93100	1.65290	-0.70860
P	1.14430	-0.01910	0.30640
C	-1.72500	-0.88880	3.20920
C	-1.20410	-2.90200	1.86860
C	2.12410	-2.19520	4.29530
C	3.15010	-1.57650	3.58360
C	2.85590	-0.90300	2.40660
C	1.54160	-0.82050	1.90840
C	0.52500	-1.45380	2.62730
C	0.82350	-2.13440	3.81590
H	2.33430	-2.73380	5.22320
H	4.18210	-1.61920	3.94080
H	3.67770	-0.43630	1.86950
H	0.03530	-2.63810	4.37750
H	-1.32270	-3.47080	2.80630
H	-2.13240	-2.97400	1.29200
H	-0.39390	-3.34570	1.27470
H	-1.66290	-1.47030	4.14290
H	-1.39230	0.14240	3.39930
H	-2.77060	-0.86870	2.88680
C	2.40580	-3.48520	-1.66070
C	1.89260	-3.16500	-3.07070
C	2.23530	-1.70920	-3.40860
C	1.56240	-0.77920	-2.38710
C	2.07200	-1.07370	-0.95940
C	1.73470	-2.55170	-0.64010
H	2.15360	-4.52630	-1.39720
H	0.79850	-3.31850	-3.12230
H	2.35330	-3.84710	-3.80750
H	1.85160	-1.45600	-4.41100
H	1.77400	0.26600	-2.66310
H	0.47110	-0.93390	-2.43050
H	0.64010	-2.70090	-0.67000
H	2.07880	-2.81720	0.37320
C	3.75500	-1.50460	-3.36490
H	4.00570	-0.46060	-3.62750
H	4.24740	-2.15680	-4.10830
C	3.92750	-3.28740	-1.60650
H	4.30550	-3.53310	-0.59740
H	4.42380	-3.97210	-2.31740
C	3.60470	-0.89040	-0.93170
H	4.00650	-1.12720	0.06400
H	3.87120	0.15490	-1.15940
C	4.26570	-1.83060	-1.95590
H	5.35740	-1.68020	-1.90930
C	3.32380	3.48270	1.43310
C	3.54260	4.10030	0.04740
C	2.26310	3.92720	-0.77800
C	1.94510	2.43030	-0.92660
C	1.70810	1.78850	0.45800
C	2.99120	1.98570	1.29680
H	4.24410	3.57380	2.03460
H	4.39130	3.60630	-0.45990
H	3.79600	5.17160	0.14280
H	2.40570	4.35060	-1.78650
H	1.05360	2.32780	-1.55440
H	2.78010	1.92750	-1.44210
H	3.84140	1.46370	0.82590
H	2.85280	1.57630	2.30870
C	1.09160	4.63420	-0.08090
H	0.17020	4.52250	-0.68100
H	1.29910	5.71620	0.00310
C	2.16650	4.19700	2.14390
H	2.02310	3.77490	3.15530
H	2.40000	5.26980	2.26690
C	0.55240	2.53430	1.17120
H	0.37870	2.08930	2.16860
H	-0.38230	2.43000	0.59490
C	0.88700	4.02750	1.31500

H	0.04280	4.52720	1.81920
Au	-1.30060	-0.27810	0.25650
O	-2.39930	-2.47220	-2.89030
C	-1.87760	-2.73110	-1.92430

MeDalPhosAu(biphenylene)CO bound (Int2)

SCF = –2196.40340
ZPE = –2195.59040

Au	1.23830	-0.66650	-0.40540
O	0.17730	-2.79330	-2.50250
C	0.44440	-2.04740	-1.70430
C	5.75430	-1.64150	-1.97610
C	5.45550	-0.51990	-1.20230
C	4.15610	-0.31140	-0.73200
C	3.13770	-1.22760	-1.06190
C	3.45010	-2.36120	-1.81400
C	4.75800	-2.56870	-2.26800
H	6.77460	-1.79660	-2.33800
H	6.25250	0.18590	-0.95310
H	2.70380	-3.11950	-2.06160
H	4.98810	-3.46250	-2.85500
C	4.18450	2.55430	1.76030
C	2.87810	2.45460	2.23150
C	1.99650	1.53230	1.66150
C	2.40140	0.70490	0.61680
C	3.74870	0.75310	0.19720
C	4.62110	1.69350	0.75540
H	4.87530	3.28410	2.19210
H	2.53070	3.09590	3.04660
H	0.98940	1.47020	2.07040
H	5.66130	1.74230	0.42240
P	-1.01140	0.21160	0.26700
C	-1.44080	0.25670	4.89970
C	-2.18300	1.11880	4.09670
C	-2.01420	1.07880	2.71680
C	-1.10320	0.20280	2.10400
C	-0.33560	-0.66600	2.92530
C	-0.53370	-0.61930	4.31230
H	-1.56450	0.26530	5.98630
H	-2.89740	1.81890	4.53740
H	-2.61620	1.74900	2.10220
H	0.05010	-1.28890	4.94900
C	1.97490	-1.39770	3.05410
N	0.67350	-1.54940	2.40380
C	0.31860	-2.96440	2.41890
H	2.21470	-0.33950	3.21640
H	2.02150	-1.92300	4.03120
H	2.75580	-1.82840	2.40360
H	-0.67010	-3.13670	1.98430
H	1.06080	-3.52690	1.82630
H	0.31730	-3.38160	3.44960
C	-0.47970	4.39310	-0.39980
C	0.13070	4.27920	-1.80330
C	-0.66840	3.25170	-2.61550
C	-0.59880	1.88240	-1.91880
C	-1.21680	1.96220	-0.49440
C	-0.41250	3.02060	0.29150
H	0.10630	5.10620	0.20440
H	1.18870	3.96760	-1.73260
H	0.11030	5.26160	-2.30850
H	-0.23030	3.14750	-3.62240
H	-1.11740	1.13040	-2.53440
H	0.45940	1.57280	-1.84980
H	0.64180	2.72670	0.33190
H	-0.78020	3.10390	1.32830
C	-2.12960	3.70570	-2.72580
H	-2.71150	2.97540	-3.31730
H	-2.18710	4.67450	-3.25370
C	-1.93290	4.86420	-0.50410
H	-2.37280	4.97040	0.50420
H	-1.98060	5.85550	-0.98940
C	-2.67510	2.46840	-0.61350
H	-3.13440	2.56830	0.38300
H	-3.29220	1.77050	-1.19310
C	-2.72430	3.83520	-1.31760
H	-3.78150	4.14260	-1.38670
C	-4.97990	-1.35960	0.03370
C	-5.15750	-1.30910	-1.48990
C	-3.86710	-1.79080	-2.16700
C	-2.71870	-0.84860	-1.77250
C	-2.50550	-0.86700	-0.24180
C	-3.82090	-0.43870	0.46270
H	-5.89870	-1.00180	0.52800
H	-5.39200	-0.27860	-1.81370
H	-6.00680	-1.94760	-1.79190
H	-3.98340	-1.75250	-3.26300
H	-1.80190	-1.14160	-2.30910
H	-2.97000	0.16260	-2.12060
H	-4.08940	0.59930	0.23240
H	-3.69790	-0.51160	1.55530
C	-3.54380	-3.22390	-1.72690
H	-2.61760	-3.57270	-2.21830
H	-4.35370	-3.90830	-2.03640
C	-4.68400	-2.79990	0.46610
H	-4.58770	-2.85530	1.56540
H	-5.51610	-3.46540	0.17460
C	-2.24370	-2.31490	0.22260

H	-2.17910	-2.31950	1.32070
H	-1.28740	-2.70700	-0.16080
C	-3.38190	-3.25670	-0.20230
H	-3.12620	-4.27730	0.12830

MeDalPhosAu(biphenylene)(CO) TS (TS1)

SCF = –2196.35867
ZPE = –2195.54823

Au	1.15280	-0.24340	-0.11240
O	1.38940	-1.37690	-2.90940
C	1.45020	-1.24350	-1.76730
C	5.54840	-2.28410	-0.56190
C	5.34970	-0.90800	-0.65440
C	4.06110	-0.35860	-0.69460
C	2.97570	-1.27110	-0.68630
C	3.17160	-2.65300	-0.53980
C	4.46470	-3.16060	-0.47880
H	6.56850	-2.67580	-0.52350
H	6.21840	-0.24540	-0.63000
H	2.31490	-3.33340	-0.52360
H	4.62410	-4.23770	-0.38580
C	4.49890	3.40150	-0.54310
C	3.25700	3.77480	-0.02780
C	2.27580	2.80730	0.20200
C	2.50560	1.45000	-0.04180
C	3.78360	1.08270	-0.54100
C	4.76000	2.05880	-0.79760
H	5.26450	4.15570	-0.74660
H	3.04540	4.82640	0.19040
H	1.31070	3.13820	0.58960
H	5.73100	1.77430	-1.21360
P	-1.18440	0.06590	0.28270
C	-1.77170	-0.37960	4.86080
C	-2.82260	0.05660	4.05480
C	-2.62290	0.19380	2.68660
C	-1.38020	-0.09200	2.09530
C	-0.31960	-0.51800	2.91450
C	-0.53510	-0.66120	4.29200
H	-1.91310	-0.49870	5.93860
H	-3.79830	0.28920	4.48900
H	-3.45350	0.53750	2.06810
H	0.28160	-1.00100	4.93370
C	2.02920	0.03440	2.98220
N	0.99250	-0.80970	2.37360
C	1.33580	-2.22960	2.51390
H	1.75870	1.09500	2.87110
H	2.17400	-0.19370	4.05600
H	2.98390	-0.13700	2.46080
H	0.57290	-2.85240	2.02300
H	2.30920	-2.41200	2.03250
H	1.41340	-2.53350	3.57650
C	-1.79150	4.20570	0.38800
C	-1.03670	4.55150	-0.90330
C	-1.43090	3.55740	-2.00420
C	-1.05380	2.13710	-1.55820
C	-1.81070	1.75990	-0.25790
C	-1.44950	2.77860	0.84760
H	-1.50430	4.90730	1.18930
H	0.05360	4.51480	-0.73400
H	-1.28330	5.58200	-1.21630
H	-0.87960	3.78860	-2.93110
H	-1.29170	1.41620	-2.35930
H	0.03470	2.08890	-1.38850
H	-0.38220	2.69790	1.10900
H	-2.01600	2.55860	1.76630
C	-2.94120	3.63690	-2.25990
H	-3.22850	2.93330	-3.06260
H	-3.21600	4.65150	-2.60000
C	-3.30350	4.29270	0.13540
H	-3.85770	4.06750	1.06470
H	-3.58020	5.31750	-0.17080
C	-3.32960	1.86300	-0.52570
H	-3.90190	1.60930	0.38140
H	-3.63520	1.16300	-1.31820
C	-3.68970	3.29450	-0.96470
H	-4.77790	3.33620	-1.14050
C	-4.31930	-2.68140	-0.58980
C	-4.29870	-2.55030	-2.11880
C	-2.84340	-2.49660	-2.60430
C	-2.15190	-1.27080	-1.98810
C	-2.15830	-1.37540	-0.44720
C	-3.61930	-1.46920	0.05350
H	-5.36200	-2.70530	-0.23090
H	-4.83510	-1.63540	-2.43030
H	-4.82160	-3.40800	-2.57860
H	-2.81850	-2.39680	-3.70230
H	-1.11920	-1.20080	-2.36530
H	-2.67740	-0.35920	-2.31430
H	-4.18280	-0.55590	-0.18450
H	-3.63470	-1.59610	1.14780
C	-2.10290	-3.77270	-2.18160
H	-1.05550	-3.73450	-2.53380
H	-2.57300	-4.65670	-2.64860
C	-3.59800	-3.96990	-0.17500
H	-3.63250	-4.08950	0.92310
H	-4.10240	-4.84800	-0.61680
C	-1.44950	-2.68390	-0.02170

H	-1.46630	-2.77380	1.07910
H	-0.39310	-2.66820	-0.33590
C	-2.14210	-3.90190	-0.65220
H	-1.60100	-4.81100	-0.33970

MeDalPhosAu(biphenylene)(CO) TS (TS2)

SCF = –2196.33627
ZPE = –2195.52694

N	-0.81390	-1.82210	1.84120
C	-2.90280	3.06640	-2.92860
C	-3.71060	2.43660	-1.99160
C	-3.20240	1.38180	-1.21160
C	-1.85030	0.97280	-1.35650
C	-1.08700	1.56260	-2.36960
C	-1.59590	2.61240	-3.13430
H	-3.30110	3.88840	-3.53050
H	-4.75880	2.73220	-1.88810
H	-0.06950	1.22900	-2.55970
H	-0.96260	3.08510	-3.89090
C	-5.95660	-0.06680	0.98070
C	-5.39710	-1.33350	1.19960
C	-4.17290	-1.70090	0.67240
C	-3.39250	-0.78160	-0.15970
C	-4.00950	0.54960	-0.34220
C	-5.25120	0.85100	0.20280
H	-6.92300	0.19850	1.41500
H	-5.93720	-2.07080	1.80180
H	-3.82640	-2.72640	0.80710
H	-5.65880	1.85390	0.04100
P	1.24600	-0.09570	0.28400
C	-1.78650	-1.38260	2.86680
C	-1.07310	-3.20810	1.38820
C	2.06430	-2.58350	4.11910
C	3.11460	-1.90310	3.50470
C	2.86660	-1.15930	2.35920
C	1.58000	-1.07100	1.79720
C	0.53980	-1.78170	2.40680
C	0.79110	-2.52610	3.56820
H	2.23600	-3.17040	5.02550
H	4.12610	-1.94900	3.91650
H	3.69930	-0.63730	1.89060
H	-0.01370	-3.08280	4.05210
H	-1.06490	-3.91620	2.23570
H	-2.05090	-3.26990	0.89700
H	-0.29390	-3.50020	0.66880
H	-1.82690	-2.08550	3.71630
H	-1.49480	-0.38590	3.23090
H	-2.79250	-1.32530	2.42790
C	3.05880	-3.07200	-2.01960
C	2.51070	-2.65960	-3.39220
C	2.62910	-1.13790	-3.54280
C	1.82250	-0.45150	-2.42970
C	2.36480	-0.84360	-1.03670
C	2.25350	-2.38250	-0.90640
H	2.96750	-4.16390	-1.89230
H	1.45400	-2.96910	-3.48900
H	3.07460	-3.16740	-4.19530
H	2.21650	-0.82380	-4.51640
H	1.87810	0.64060	-2.56540
H	0.76280	-0.74990	-2.51120
H	1.19150	-2.68830	-0.96670
H	2.63460	-2.71620	0.07270
C	4.10180	-0.71850	-3.44140
H	4.19580	0.37510	-3.57180
H	4.68840	-1.19520	-4.24730
C	4.53360	-2.65740	-1.90670
H	4.94000	-2.96680	-0.92650
H	5.13040	-3.16880	-2.68330
C	3.85090	-0.43320	-0.95780
H	4.28310	-0.70840	0.01640
H	3.94810	0.65940	-1.06720
C	4.65080	-1.13490	-2.07030
H	5.70790	-0.83180	-1.98360
C	3.11100	3.39860	1.89990
C	3.16110	4.23710	0.61620
C	1.85320	4.04220	-0.16070
C	1.69560	2.55840	-0.52520
C	1.63840	1.69140	0.74990
C	2.93830	1.90750	1.55500
H	4.05390	3.51650	2.46060
H	4.02100	3.93020	-0.00720
H	3.30300	5.30400	0.86630
H	1.87990	4.62950	-1.09410
H	0.77370	2.42860	-1.10840
H	2.54250	2.24780	-1.15960
H	3.81400	1.55290	0.98720
H	2.89680	1.34230	2.49870
C	0.66130	4.48830	0.69800
H	-0.28090	4.35510	0.13490
H	0.74970	5.56260	0.94080
C	1.93120	3.85650	2.76830
H	1.90500	3.27730	3.70930
H	2.05070	4.92050	3.04180
C	0.45450	2.17270	1.62730
H	0.39830	1.56380	2.54860
H	-0.49950	2.04320	1.08720
C	0.62480	3.65700	1.98900

H	-0.23340	3.96740	2.60860
Au	-1.21980	-0.45300	0.01920
O	-4.17600	-1.88950	-2.38950
C	-3.53950	-1.44460	-1.55110

MeDalPhosAu(C=O)(biphenylene)

(Int3)

SCF = –2196.46149
ZPE = –2195.64711

Au	-0.81400	-0.99910	-0.66520
N	-0.29610	-2.74290	0.69160
C	-2.41410	2.79710	-3.21660
C	-3.19750	2.23210	-2.21290
C	-2.78900	1.06820	-1.54450
C	-1.54340	0.49950	-1.86680
C	-0.80360	1.02220	-2.92810
C	-1.22980	2.17080	-3.59760
H	-2.75190	3.70370	-3.72620
H	-4.16620	2.68020	-1.97600
H	0.12840	0.55180	-3.23810
H	-0.62640	2.57500	-4.41560
C	-5.62220	0.37690	0.90870
C	-5.65010	-1.01750	0.90040
C	-4.71160	-1.71920	0.14500
C	-3.74380	-1.02340	-0.58330
C	-3.73060	0.38490	-0.61310
C	-4.68190	1.07210	0.14560
H	-6.34580	0.93460	1.50990
H	-6.39840	-1.55920	1.48510
H	-4.71200	-2.81270	0.13270
H	-4.69080	2.16530	0.15040
P	1.13110	0.00940	0.44200
C	0.10580	-3.83560	-0.22860
C	2.11300	-3.30260	3.51910
C	2.77230	-2.07680	3.59640
C	2.44180	-1.06880	2.70030
C	1.44300	-1.24890	1.72840
C	0.77120	-2.47850	1.67100
C	1.12460	-3.49980	2.56230
H	2.36980	-4.11570	4.20320
H	3.55370	-1.91110	4.34240
H	2.99240	-0.12830	2.74120
H	0.62920	-4.47150	2.51730
H	1.05020	-3.56510	-0.72400
H	0.23740	-4.78690	0.31560
H	-0.67790	-3.97040	-0.99010
C	4.66610	-1.18920	-1.46360
C	4.32420	-0.70150	-2.87770
C	3.70130	0.69780	-2.78910
C	2.42670	0.62140	-1.93620
C	2.75610	0.16160	-0.50010
C	3.39530	-1.24490	-0.59880
H	5.09940	-2.20240	-1.51140
H	3.61860	-1.40060	-3.36240
H	5.23710	-0.67480	-3.49940
H	3.42680	1.05090	-3.79760
H	1.91580	1.59850	-1.92800
H	1.73930	-0.11140	-2.38980
H	2.67140	-1.95880	-1.03440
H	3.66210	-1.62000	0.40210
C	4.69860	1.67170	-2.14770
H	4.26010	2.68490	-2.09490
H	5.61140	1.74200	-2.76630
C	5.67180	-0.22450	-0.81810
H	5.94220	-0.57770	0.19380
H	6.60210	-0.19430	-1.41320
C	3.77800	1.13450	0.12280
H	4.03900	0.81970	1.14660
H	3.35220	2.14860	0.18380
C	5.05270	1.17790	-0.73800
H	5.76690	1.87460	-0.26740
C	0.80350	3.36880	3.06040
C	0.67440	4.45280	1.98150
C	-0.23070	3.94060	0.85260
C	0.40010	2.68610	0.22980
C	0.54340	1.57680	1.29750
C	1.42150	2.09260	2.45820
H	1.46460	3.72360	3.86900
H	1.67110	4.71140	1.57970
H	0.25110	5.37370	2.42170
H	-0.32600	4.71020	0.06830
H	-0.22560	2.33210	-0.60580
H	1.38870	2.94440	-0.18550
H	2.44610	2.30600	2.11840
H	1.48230	1.33060	3.25170
C	-1.61860	3.59640	1.41130
H	-2.27030	3.22790	0.59780
H	-2.09420	4.50140	1.83060
C	-0.58340	3.04320	3.63070
H	-0.49760	2.28210	4.42750
H	-1.02960	3.94590	4.08550
C	-0.85730	1.26040	1.88420
H	-0.77190	0.46910	2.65110
H	-1.53280	0.88780	1.09630
C	-1.47920	2.52310	2.50000
H	-2.47490	2.26170	2.89700
C	-2.64020	-1.78600	-1.24120
O	-2.76830	-2.80440	-1.86190

C	-1.53920	-3.11490	1.41390
H	-1.88510	-2.26070	2.01490
H	-2.31240	-3.39470	0.68740
H	-1.37480	-3.98170	2.07430

MeDalPhosAu(C=O)(biphenylene) TS (TS3)

SCF = –2196.44664
ZPE = –2195.63474

Au	-0.76560	-0.94840	-0.64130
N	-0.31420	-2.74780	0.85580
C	-2.21940	2.76670	-3.21330
C	-3.00240	2.36460	-2.12840
C	-2.79680	1.11150	-1.55360
C	-1.77060	0.27840	-2.04210
C	-1.06110	0.64160	-3.19020
C	-1.27800	1.89530	-3.76460
H	-2.37870	3.75180	-3.66050
H	-3.80530	3.01070	-1.76230
H	-0.31330	-0.03060	-3.61830
H	-0.70060	2.19440	-4.64370
C	-5.48660	0.26260	1.02930
C	-5.48140	-1.12520	0.88580
C	-4.59040	-1.73810	-0.00240
C	-3.70990	-0.93650	-0.71870
C	-3.72450	0.46110	-0.59620
C	-4.61920	1.06660	0.27830
H	-6.18430	0.73220	1.72840
H	-6.17870	-1.73670	1.46500
H	-4.58160	-2.82390	-0.13010
H	-4.65040	2.15480	0.37930
P	1.15030	-0.02780	0.50630
C	0.04070	-3.88070	-0.02080
C	2.08940	-3.26200	3.68660
C	2.83650	-2.08510	3.66030
C	2.54430	-1.11300	2.71120
C	1.50590	-1.28460	1.78070
C	0.76000	-2.47780	1.80920
C	1.06550	-3.45440	2.76600
H	2.30670	-4.04080	4.42280
H	3.64960	-1.92670	4.37360
H	3.14700	-0.20350	2.68560
H	0.50080	-4.38910	2.79370
H	0.99510	-3.66720	-0.52620
H	0.13810	-4.82320	0.55040
H	-0.74620	-4.01040	-0.78030
C	4.51620	-1.17440	-1.67730
C	4.07460	-0.64320	-3.04770
C	3.47680	0.75980	-2.87550
C	2.26600	0.67730	-1.93390
C	2.69840	0.16630	-0.53810
C	3.30990	-1.24250	-0.72670
H	4.93390	-2.18950	-1.78610
H	3.32540	-1.32180	-3.49490
H	4.93760	-0.60940	-3.73690
H	3.13620	1.14470	-3.85190
H	1.77940	1.66340	-1.85850
H	1.52450	-0.01900	-2.35900
H	2.54590	-1.93130	-1.13530
H	3.63710	-1.65320	0.24270
C	4.53150	1.70400	-2.28180
H	4.11140	2.72030	-2.17030
H	5.39760	1.78090	-2.96360
C	5.57620	-0.23760	-1.07900
H	5.91360	-0.62310	-0.09960
H	6.46230	-0.19920	-1.73800
C	3.77130	1.11330	0.03450
H	4.09930	0.77200	1.03060
H	3.35800	2.12830	0.14970
C	4.98130	1.16830	-0.91500
H	5.73690	1.84440	-0.48040
C	0.87060	3.33970	3.11510
C	0.65020	4.40820	2.03450
C	-0.29700	3.85730	0.95890
C	0.33960	2.61600	0.31740
C	0.57170	1.52360	1.38680
C	1.49660	2.07840	2.49020
H	1.56060	3.72530	3.88450
H	1.61590	4.69310	1.57860
H	0.22210	5.32040	2.48800
H	-0.45510	4.61600	0.17410
H	-0.31390	2.23240	-0.48650
H	1.29970	2.90010	-0.14490
H	2.49150	2.32120	2.08620
H	1.63300	1.32400	3.28240
C	-1.64240	3.47730	1.59270
H	-2.32240	3.07850	0.81740
H	-2.12450	4.37200	2.02630
C	-0.47430	2.97770	3.75990
H	-0.32410	2.22710	4.55700
H	-0.92280	3.87090	4.23110
C	-0.78680	1.17090	2.04720
H	-0.63830	0.39010	2.81490
H	-1.48910	0.76430	1.29720
C	-1.41400	2.42030	2.68290
H	-2.37930	2.13570	3.13530
C	-2.61490	-1.45550	-1.60010
O	-2.65360	-2.38220	-2.36130

C	-1.59040	-3.00210	1.55060
H	-1.86980	-2.11950	2.14630
H	-2.37140	-3.19310	0.80120
H	-1.53180	-3.88330	2.21520

9-Fluorenone product

SCF = –574.82846
ZPE = –574.65654

C	-2.53940	0.98440	-0.00010
C	-3.46460	-0.06720	0.00020
C	-3.02590	-1.39360	0.00030
C	-1.65910	-1.70950	-0.00010
C	-0.74330	-0.66600	-0.00050
C	0.74320	-0.66600	-0.00050
C	1.65910	-1.70950	0.00000
C	3.02580	-1.39360	0.00030
C	3.46460	-0.06720	0.00020
C	2.53940	0.98440	-0.00020
C	1.18970	0.66670	-0.00050
C	0.00000	1.57910	-0.00010
C	-1.18970	0.66670	-0.00040
H	-2.86650	2.02810	-0.00020
H	-4.53660	0.14930	0.00070
H	-3.76280	-2.20240	0.00110
H	-1.33180	-2.75270	0.00010
H	1.33170	-2.75270	0.00030
H	3.76280	-2.20240	0.00110
H	4.53660	0.14910	0.00060
H	2.86640	2.02810	-0.00030
O	0.00000	2.78790	0.00060

IPrAu(biphenylene)

SCF = –1756.12219
ZPE = –1755.38177

Au	-0.66510	0.66080	-0.04470
C	0.46620	-1.13960	-0.11590
N	1.73440	-1.49850	-0.40800
C	1.81460	-2.86510	-0.60130
C	0.56990	-3.36910	-0.39830
N	-0.24230	-2.29190	-0.11250
H	2.75010	-3.35390	-0.86080
H	0.19490	-4.38880	-0.42830
C	-4.36010	-2.21720	0.79950
C	-3.95160	-2.31280	-0.52620
C	-2.59720	-2.36630	-0.88110
C	-1.65870	-2.32450	0.17210
C	-2.04770	-2.25810	1.52710
C	-3.41350	-2.19320	1.81640
H	-5.42550	-2.16350	1.04000
H	-4.70360	-2.34200	-1.32040
H	-3.73960	-2.12440	2.85790
C	4.94200	1.22120	-0.71790
C	4.84740	0.38360	0.38700
C	3.80410	-0.54120	0.52800
C	2.85280	-0.59410	-0.51180
C	2.93650	0.22820	-1.65300
C	3.99370	1.13900	-1.73000
H	5.76150	1.94190	-0.78990
H	5.59850	0.45280	1.17980
H	4.07900	1.79200	-2.60270
C	2.49200	-1.71150	2.46080
C	3.83410	-1.39360	1.79760
C	4.66180	-2.66990	1.58940
H	1.81830	-0.83890	2.47950
H	1.97500	-2.54880	1.96720
H	2.66690	-2.01740	3.50690
H	4.39380	-0.77970	2.52240
H	5.66510	-2.43630	1.19360
H	4.78910	-3.19970	2.55020
H	4.16970	-3.36350	0.88650
C	1.34910	1.47870	-3.19300
C	1.96080	0.12740	-2.81280
C	2.62450	-0.54880	-4.01870
H	0.89240	1.97630	-2.31880
H	2.09690	2.16810	-3.62250
H	0.55970	1.33480	-3.95200
H	1.13460	-0.52030	-2.50340
H	3.01740	-1.54550	-3.75140
H	1.89220	-0.67920	-4.83540
H	3.46410	0.05540	-4.40710
C	-1.03170	-0.90500	3.39810
C	-1.04480	-2.25240	2.66800
C	-1.28750	-3.41330	3.63910
H	-0.81360	-0.07370	2.70360
H	-2.00550	-0.69850	3.87770
H	-0.25870	-0.90290	4.18610
H	-0.04640	-2.40190	2.24010
H	-1.28900	-4.38290	3.11120
H	-0.48730	-3.44050	4.39960
H	-2.25010	-3.31080	4.17090
C	-1.15440	-1.63910	-2.91460
C	-2.28930	-2.50660	-2.36910
C	-2.09530	-3.97840	-2.75840
H	-1.19630	-0.60870	-2.51440
H	-0.16490	-2.06750	-2.69140
H	-1.23430	-1.57080	-4.01320
H	-3.20780	-2.16770	-2.87560

H	-2.95520	-4.59240	-2.44010
H	-1.99820	-4.07000	-3.85460
H	-1.18350	-4.40130	-2.30310
C	-3.85270	4.24060	-0.43400
C	-2.58830	4.48360	0.11040
C	-1.67860	3.43280	0.22820
C	-2.03930	2.14500	-0.20380
C	-3.29890	1.89880	-0.73840
C	-4.20930	2.95890	-0.85360
H	-4.56850	5.06230	-0.52750
H	-2.32050	5.49080	0.44320
H	-3.59070	0.89470	-1.06680
H	-5.20270	2.77660	-1.27390
C	1.63740	4.40730	1.85600
C	0.35060	4.56230	1.33420
C	-0.32470	3.46810	0.79070
C	0.33390	2.22450	0.78340
C	1.60120	2.04740	1.30420
C	2.25840	3.16060	1.84640
H	2.15660	5.27200	2.27840
H	-0.13310	5.54310	1.35240
H	2.09280	1.07470	1.29350
H	3.26390	3.03750	2.25840

IPrAu(biphenylene)(CO) bound

SCF = –1869.32212
ZPE = –1868.57282

C	-0.24230	-1.24770	-0.14630
N	0.66590	-2.21900	-0.37130
C	0.03820	-3.41070	-0.69070
C	-1.29870	-3.18000	-0.62870
N	-1.46150	-1.84050	-0.32110
H	0.60050	-4.31680	-0.90210
H	-2.14830	-3.84060	-0.78120
C	-5.22190	-0.01590	0.23090
C	-4.74630	-0.23490	-1.05350
C	-3.49360	-0.82250	-1.29120
C	-2.73640	-1.19070	-0.15990
C	-3.19050	-0.96210	1.16070
C	-4.44420	-0.37120	1.32830
H	-6.20350	0.44260	0.38100
H	-5.35730	0.05340	-1.91410
H	-4.82200	-0.18120	2.33510
C	4.83150	-1.58110	-0.33460
C	4.22200	-2.22640	0.73080
C	2.83620	-2.45480	0.76490
C	2.09060	-2.00790	-0.33980
C	2.68730	-1.35930	-1.44950
C	4.06710	-1.15010	-1.41560
H	5.91250	-1.41440	-0.32660
H	4.82840	-2.56600	1.57640
H	4.55870	-0.64740	-2.25030
C	0.92390	-2.90720	2.50770
C	2.32470	-3.22280	1.98560
C	2.49930	-4.73170	1.74950
H	0.79850	-1.82540	2.67790
H	0.11720	-3.25980	1.84770
H	0.78830	-3.41110	3.48020
H	3.01160	-2.93710	2.79910
H	3.53870	-4.97910	1.47370
H	2.24200	-5.29780	2.66230
H	1.83890	-5.08090	0.93560
C	2.32590	0.37670	-3.30420
C	1.87950	-0.94510	-2.67450
C	1.87200	-2.08270	-3.70440
H	2.37230	1.18580	-2.55450
H	3.31360	0.30110	-3.79070
H	1.60300	0.67810	-4.08260
H	0.84210	-0.79600	-2.35810
H	1.48050	-3.01700	-3.26590
H	1.23720	-1.81760	-4.56830
H	2.89270	-2.28440	-4.07750
C	-2.49780	-0.41450	3.55470
C	-2.37450	-1.37920	2.37470
C	-2.73230	-2.81570	2.78120
H	-2.30730	0.62820	3.24300
H	-3.49550	-0.45330	4.02700
H	-1.75870	-0.67830	4.33180
H	-1.31830	-1.38290	2.08340
H	-2.58430	-3.51640	1.93980
H	-2.09830	-3.14960	3.62110
H	-3.78890	-2.88580	3.09840
C	-1.67740	-0.85880	-3.17950
C	-3.13620	-1.03300	-2.76510
C	-3.69900	-2.37380	-3.26170
H	-1.25300	0.09210	-2.80760
H	-1.04670	-1.69250	-2.84280
H	-1.61810	-0.84090	-4.28180
H	-3.69390	-0.24230	-3.29300
H	-4.77830	-2.44820	-3.04400
H	-3.56020	-2.47240	-4.35300
H	-3.19320	-3.22740	-2.77780
C	-2.34120	4.85190	-0.33340
C	-0.94670	4.94560	-0.28110
C	-0.18090	3.79110	-0.11320
C	-0.84310	2.55650	-0.00820
C	-2.22120	2.44440	-0.04540
C	-2.97530	3.61570	-0.21210
H	-2.94400	5.75520	-0.46560

H	-0.46000	5.92170	-0.36500
H	-2.72190	1.47810	0.04700
H	-4.06640	3.54690	-0.24880
C	3.61280	4.21250	0.16940
C	2.28350	4.60270	-0.01560
C	1.27250	3.63970	0.01400
C	1.62600	2.29850	0.22090
C	2.93490	1.89600	0.42720
C	3.93960	2.87460	0.39480
H	4.40820	4.96330	0.14530
H	2.04030	5.65690	-0.17860
H	3.19710	0.84730	0.60820
H	4.98170	2.57890	0.54620
Au	0.22600	0.82940	0.15100
O	1.11470	1.52990	3.61050
C	0.63410	0.73900	2.96370

IPrAu(biphenylene)(CO) TS

SCF = –1869.26058
ZPE = –1868.51273

Au	0.41370	-0.67110	0.11120
C	-0.82880	0.98170	0.11520
N	-2.17080	1.01330	-0.03430
C	-2.64200	2.29600	0.16640
C	-1.56750	3.06580	0.47490
N	-0.45980	2.24450	0.41830
H	-3.69570	2.54260	0.06580
H	-1.48890	4.12220	0.71760
C	3.54010	3.35060	1.14940
C	2.93710	3.68980	-0.05560
C	1.60450	3.35910	-0.33680
C	0.89960	2.65980	0.66190
C	1.47820	2.32200	1.90200
C	2.81130	2.67660	2.12250
H	4.58530	3.61600	1.33140
H	3.51630	4.22370	-0.81520
H	3.28540	2.42220	3.07450
C	-4.54840	-2.28180	-1.15440
C	-4.51430	-1.87530	0.17450
C	-3.74220	-0.78530	0.59640
C	-2.99480	-0.11480	-0.39240
C	-3.01100	-0.50440	-1.74540
C	-3.80050	-1.59970	-2.10550
H	-5.15910	-3.14050	-1.44750
H	-5.10420	-2.41860	0.91910
H	-3.82400	-1.92500	-3.14950
C	-2.57510	0.04060	2.78250
C	-3.85280	-0.41520	2.07530
C	-4.99330	0.58690	2.30640
H	-1.71280	-0.60020	2.53070
H	-2.32180	1.08660	2.54780
H	-2.72460	-0.01360	3.87490
H	-4.15980	-1.35180	2.56960
H	-5.94090	0.22100	1.87510
H	-5.14930	0.74230	3.38880
H	-4.76820	1.56850	1.85570
C	-1.12930	-0.70290	-3.41040
C	-2.20350	0.21290	-2.81340
C	-3.10400	0.81250	-3.89840
H	-0.46770	-1.11030	-2.62500
H	-1.57810	-1.55570	-3.95060
H	-0.49920	-0.14360	-4.12420
H	-1.68330	1.05140	-2.33760
H	-3.85820	1.48950	-3.46010
H	-2.49880	1.39670	-4.61420
H	-3.63650	0.03050	-4.46830
C	1.38290	0.33370	3.47220
C	0.70280	1.62580	3.00870
C	0.44830	2.57910	4.18210
H	1.59010	-0.34010	2.62030
H	2.34160	0.53300	3.98280
H	0.73200	-0.20450	4.18380
H	-0.27930	1.34570	2.61150
H	-0.08600	3.48650	3.85020
H	-0.16980	2.08220	4.95100
H	1.39370	2.89500	4.65870
C	0.05570	2.96280	-2.41630
C	1.06350	3.84960	-1.68080
C	0.53730	5.28680	-1.55230
H	0.34520	1.90000	-2.39360
H	-0.96400	3.06020	-2.01160
H	0.01000	3.27080	-3.47540
H	1.95330	3.89970	-2.33040
H	1.30240	5.95740	-1.12480
H	0.25590	5.67930	-2.54560
H	-0.35600	5.33340	-0.90580
C	4.10060	-1.37380	-3.00930
C	3.61130	-2.34390	-2.13940
C	2.67250	-2.00160	-1.15380
C	2.19440	-0.66520	-1.04860
C	2.71090	0.28840	-1.93010
C	3.66480	-0.05250	-2.89210
H	4.82830	-1.64880	-3.77810
H	3.95770	-3.37720	-2.23620
H	2.37910	1.32740	-1.86350
H	4.06450	0.71700	-3.55970
C	2.82550	-4.62430	1.56310
C	3.08290	-3.96830	0.36100
C	2.23890	-2.95870	-0.11440

C	1.05110	-2.69510	0.63690
C	0.85200	-3.29620	1.90010
C	1.73520	-4.26240	2.35800
H	3.51590	-5.39880	1.90830
H	4.01060	-4.18790	-0.17340
H	-0.04190	-3.05530	2.48310
H	1.55970	-4.74960	3.32000
O	-1.31950	-3.10640	-0.39090
C	-0.55580	-2.41200	0.12640

IPrAu(C=O)(biphenylene) inserted

SCF = –1869.36025
ZPE = –1868.60965

Au	-0.10500	0.78250	-0.44040
C	-0.74850	-1.06330	0.29240
N	-2.05140	-1.29490	0.55300
C	-2.22410	-2.53130	1.13960
C	-0.98990	-3.08730	1.22480
N	-0.09370	-2.16670	0.70890
H	-3.20030	-2.89420	1.45080
H	-0.66080	-4.04160	1.62730
C	3.99080	-3.16330	0.45300
C	3.51160	-2.50980	1.58130
C	2.16980	-2.12040	1.69650
C	1.32390	-2.42340	0.61090
C	1.78970	-3.05610	-0.56190
C	3.13800	-3.42150	-0.61470
H	5.04150	-3.46230	0.39920
H	4.19500	-2.29060	2.40700
H	3.52940	-3.91840	-1.50510
C	-4.90870	1.72700	-0.07410
C	-4.77600	0.70340	-1.00780
C	-3.85640	-0.33820	-0.83390
C	-3.06690	-0.29970	0.33250
C	-3.18480	0.71760	1.30070
C	-4.12200	1.73050	1.07160
H	-5.63450	2.52770	-0.24230
H	-5.40320	0.70690	-1.90450
H	-4.23950	2.53290	1.80510
C	-2.47890	-1.96540	-2.33310
C	-3.84730	-1.44160	-1.88880
C	-4.76730	-2.59800	-1.47220
H	-1.76140	-1.14640	-2.51410
H	-2.04380	-2.66320	-1.60020
H	-2.59290	-2.52340	-3.27880
H	-4.30700	-0.98120	-2.77860
H	-5.78560	-2.23880	-1.24420
H	-4.84100	-3.33720	-2.28940
H	-4.38130	-3.12040	-0.57990
C	-1.58950	2.04470	2.75030
C	-2.36370	0.73510	2.58110
C	-3.24660	0.44070	3.79910
H	-0.96230	2.26210	1.86540
H	-2.26420	2.90540	2.90190
H	-0.92390	1.98380	3.62870
H	-1.62010	-0.07180	2.52530
H	-3.77420	-0.52250	3.68620
H	-2.63020	0.38750	4.71400
H	-4.00540	1.23020	3.94600
C	1.56520	-3.30390	-3.09270
C	0.86560	-3.36460	-1.73160
C	0.18140	-4.72860	-1.56100
H	2.15630	-2.38140	-3.21070
H	2.23260	-4.16890	-3.25240
H	0.80720	-3.32470	-3.89500
H	0.08240	-2.59290	-1.73960
H	-0.40350	-4.79280	-0.62980
H	-0.50710	-4.91670	-2.40350
H	0.93310	-5.53840	-1.54660
C	1.76290	0.15990	2.71550
C	1.79050	-1.35400	2.96340
C	0.53840	-1.82260	3.70940
H	2.68830	0.50220	2.22010
H	0.90880	0.45330	2.08210
H	1.66930	0.69930	3.67460
H	2.63220	-1.53360	3.65180
H	0.52680	-2.91830	3.83910
H	0.52580	-1.36640	4.71490
H	-0.39370	-1.52470	3.20590
C	0.68690	5.49490	-1.14470
C	1.75550	4.68520	-0.76610
C	1.59210	3.29650	-0.64870
C	0.33190	2.72960	-0.90960
C	-0.72990	3.54760	-1.31460
C	-0.55740	4.92860	-1.42280
H	0.83350	6.57460	-1.23870
H	2.73320	5.14200	-0.59080
H	-1.71210	3.11330	-1.52990
H	-1.39610	5.56160	-1.72710
C	1.63300	0.54190	-1.53360
O	1.57240	-0.07150	-2.53500
C	4.91620	2.00130	0.69960
C	3.84200	2.85330	0.43990
C	2.74300	2.41770	-0.30680
C	2.77540	1.09350	-0.78520
C	3.85990	0.24210	-0.55630
C	4.93460	0.69860	0.19940
H	5.75270	2.36430	1.30320
H	3.85780	3.86810	0.84410

H	3.84490	-0.77500	-0.95530
H	5.78270	0.03850	0.39820

IPrAu(C=O)(biphenylene) TS

SCF = –1869.35779
ZPE = –1869.60863

Au	0.00790	0.64800	-0.51350
C	-0.82770	-1.03750	0.32250
N	-2.13960	-1.16450	0.60400
C	-2.39300	-2.38660	1.19460
C	-1.20270	-3.03560	1.26160
N	-0.24920	-2.18080	0.73830
H	-3.38800	-2.67760	1.52160
H	-0.94100	-4.01300	1.65850
C	3.80700	-3.25120	0.37320
C	3.36250	-2.62000	1.52900
C	2.02810	-2.22130	1.68700
C	1.15230	-2.49840	0.61910
C	1.58110	-3.10730	-0.57810
C	2.92430	-3.48180	-0.67610
H	4.85430	-3.55350	0.28460
H	4.07000	-2.42120	2.33950
H	3.28650	-3.96210	-1.58810
C	-4.79760	2.04500	0.02250
C	-4.77990	0.99420	-0.88840
C	-3.92190	-0.10250	-0.73290
C	-3.08060	-0.09510	0.39600
C	-3.07500	0.95600	1.33610
C	-3.95250	2.02360	1.12660
H	-5.47760	2.88770	-0.13070
H	-5.44850	1.02070	-1.75440
H	-3.97940	2.84990	1.84190
C	-2.71480	-1.83390	-2.26120
C	-4.02800	-1.21620	-1.77230
C	-5.00940	-2.30300	-1.31140
H	-1.95090	-1.06610	-2.47570
H	-2.30040	-2.55290	-1.53670
H	-2.90030	-2.39130	-3.19590
H	-4.48760	-0.73420	-2.65070
H	-5.98340	-1.86860	-1.02740
H	-5.18380	-3.02830	-2.12580
H	-4.61640	-2.86010	-0.44380
C	-1.42600	2.24150	2.78550
C	-2.19490	0.93410	2.57740
C	-3.02180	0.55940	3.81340
H	-0.83810	2.51190	1.88970
H	-2.09650	3.08720	3.01750
H	-0.72410	2.13430	3.63030
H	-1.44110	0.14570	2.45000
H	-3.52860	-0.41150	3.67390
H	-2.37040	0.47960	4.70180
H	-3.79520	1.32030	4.02310
C	1.25740	-3.17270	-3.10430
C	0.62270	-3.38010	-1.72670
C	0.01730	-4.78550	-1.61330
H	1.75920	-2.19290	-3.17810
H	1.99450	-3.95850	-3.34550
H	0.47390	-3.20960	-3.88130
H	-0.20090	-2.65740	-1.64780
H	-0.51190	-4.92810	-0.65600
H	-0.70600	-4.95970	-2.42960
H	0.80550	-5.55690	-1.68380
C	1.69100	0.05610	2.73130
C	1.68940	-1.46050	2.96850
C	0.43840	-1.90230	3.73320
H	2.60930	0.37890	2.21060
H	0.82480	0.37190	2.12540
H	1.63880	0.59080	3.69640
H	2.53940	-1.66620	3.63900
H	0.39020	-2.99950	3.83930
H	0.46540	-1.46910	4.74860
H	-0.49420	-1.55890	3.25990
C	0.70220	5.34900	-0.99050
C	1.75400	4.59760	-0.46740
C	1.71600	3.20130	-0.53640
C	0.59640	2.56820	-1.10360
C	-0.42530	3.32570	-1.68740
C	-0.37690	4.71900	-1.61370
H	0.73960	6.44060	-0.93640
H	2.62350	5.10340	-0.03880
H	-1.28050	2.83700	-2.16400
H	-1.18890	5.31400	-2.04110
C	1.76480	0.80630	-1.72470
O	1.70240	0.36080	-2.81360
C	4.92170	1.73520	0.93220
C	3.88970	2.65220	0.71530
C	2.85730	2.33350	-0.16440
C	2.90000	1.09710	-0.82650
C	3.93010	0.17960	-0.62960
C	4.94660	0.50740	0.26660
H	5.72120	1.98500	1.63500
H	3.88840	3.60820	1.24480
H	3.92980	-0.77530	-1.16100
H	5.76360	-0.19700	0.44280

5.4.3. Comparison with a literature Au(III) CO complex

Figure S36: Optimized geometry for a C^N^C-ligated Au(III) CO complex originally reported by Bochmann and co-workers.[35] Starting geometry taken from a computational study by Belanzoni and co-workers.[36]

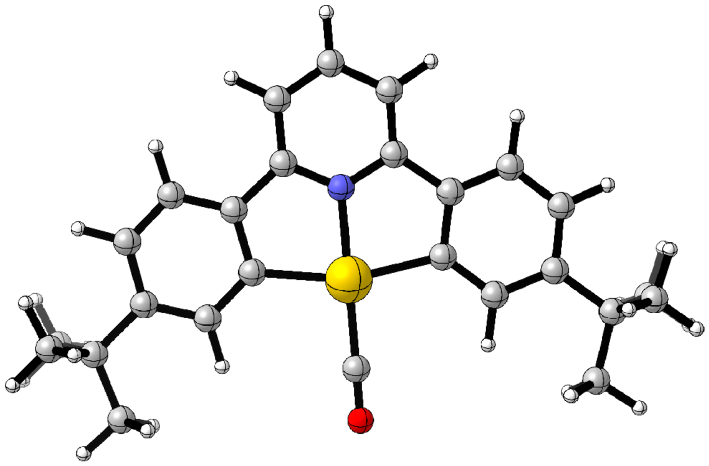

The calculated Au(III)–C=O bond length is 1.94 Å.

Cartesian coordinates (Å):

N	0.00000	0.00000	-1.75780	H	0.00000	2.89240	1.89640
C	0.00000	1.19910	-2.38590	H	0.00000	3.92350	-2.96830
C	0.00000	1.21350	-3.77740	H	0.00000	2.16070	-4.31760
C	0.00000	0.00000	-4.46340	H	0.00000	-2.89240	1.89640
C	0.00000	-1.21350	-3.77740	H	0.00000	0.00000	-5.55640
C	0.00000	-1.19910	-2.38590	H	0.00000	-2.16070	-4.31760
C	0.00000	2.35190	-1.47140	H	0.00000	-3.92350	-2.96830
C	0.00000	3.68160	-1.90180	H	0.00000	-5.74350	-1.33700
C	0.00000	4.71380	-0.97020	C	0.00000	-5.63280	1.40070
C	0.00000	4.46000	0.41050	C	0.00000	5.63280	1.40070
C	0.00000	3.11830	0.82890	C	0.00000	5.16170	2.86070
C	0.00000	2.06820	-0.08740	C	1.25880	6.48950	1.16750
Au	0.00000	0.00000	0.23860	C	-1.25880	6.48950	1.16750
C	0.00000	0.00000	2.17940	H	0.00000	6.03860	3.53130
O	0.00000	0.00000	3.30300	H	-0.89520	4.56230	3.10170
C	0.00000	-2.35190	-1.47140	H	0.89520	4.56230	3.10170
C	0.00000	-2.06820	-0.08740	H	-1.28000	7.33490	1.87830
C	0.00000	-3.11830	0.82890	H	-1.28980	6.90990	0.14760
C	0.00000	-4.46000	0.41050	H	-2.17660	5.89380	1.31880
C	0.00000	-4.71380	-0.97020	H	1.28000	7.33490	1.87830
C	0.00000	-3.68160	-1.90180	H	2.17660	5.89380	1.31880
H	0.00000	5.74350	-1.33700	H	1.28980	6.90990	0.14760

C	0.00000	-5.16170	2.86070
H	0.00000	-6.03860	3.53130
H	0.89520	-4.56230	3.10170
H	-0.89520	-4.56230	3.10170
C	1.25880	-6.48950	1.16750
C	-1.25880	-6.48950	1.16750
H	-1.28980	-6.90990	0.14760
H	-1.28000	-7.33490	1.87830
H	-2.17660	-5.89380	1.31880
H	1.28980	-6.90990	0.14760
H	2.17660	-5.89380	1.31880
H	1.28000	-7.33490	1.87830

6. Selected spectra

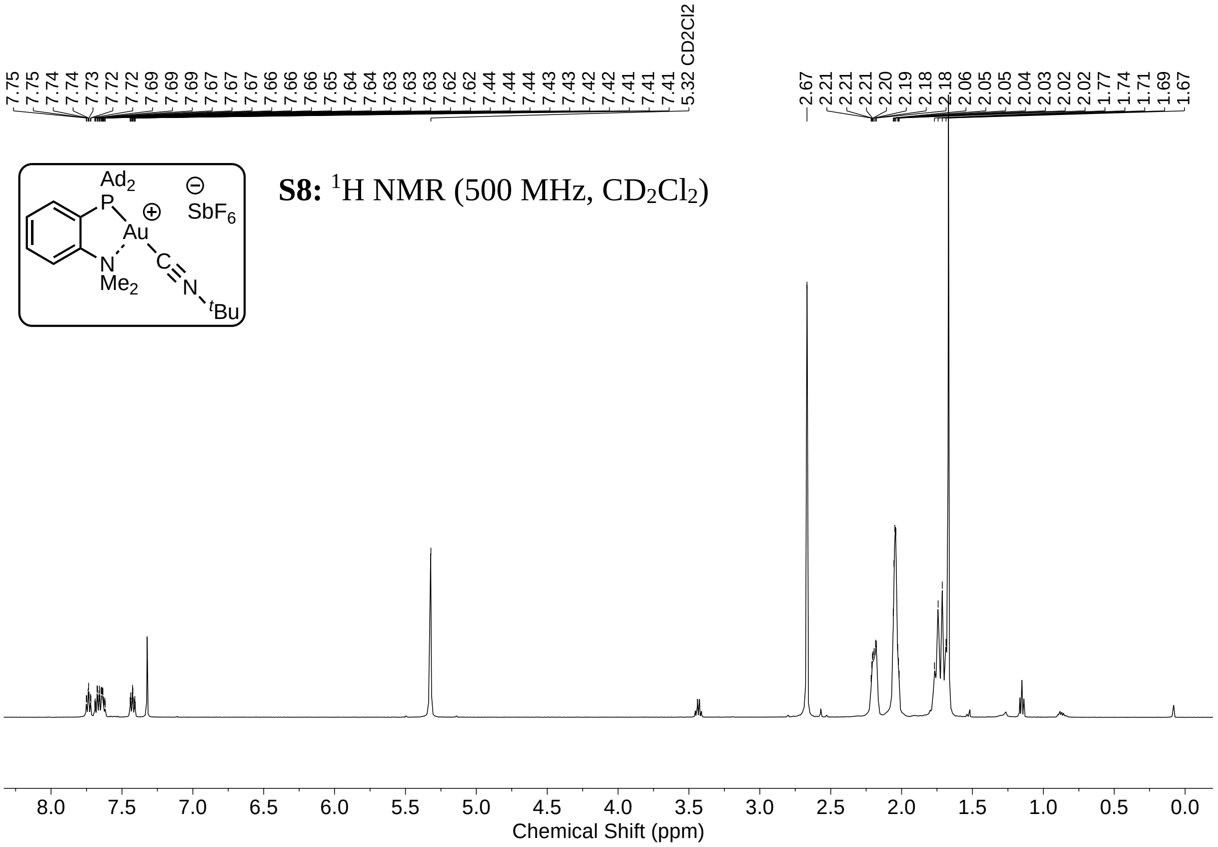

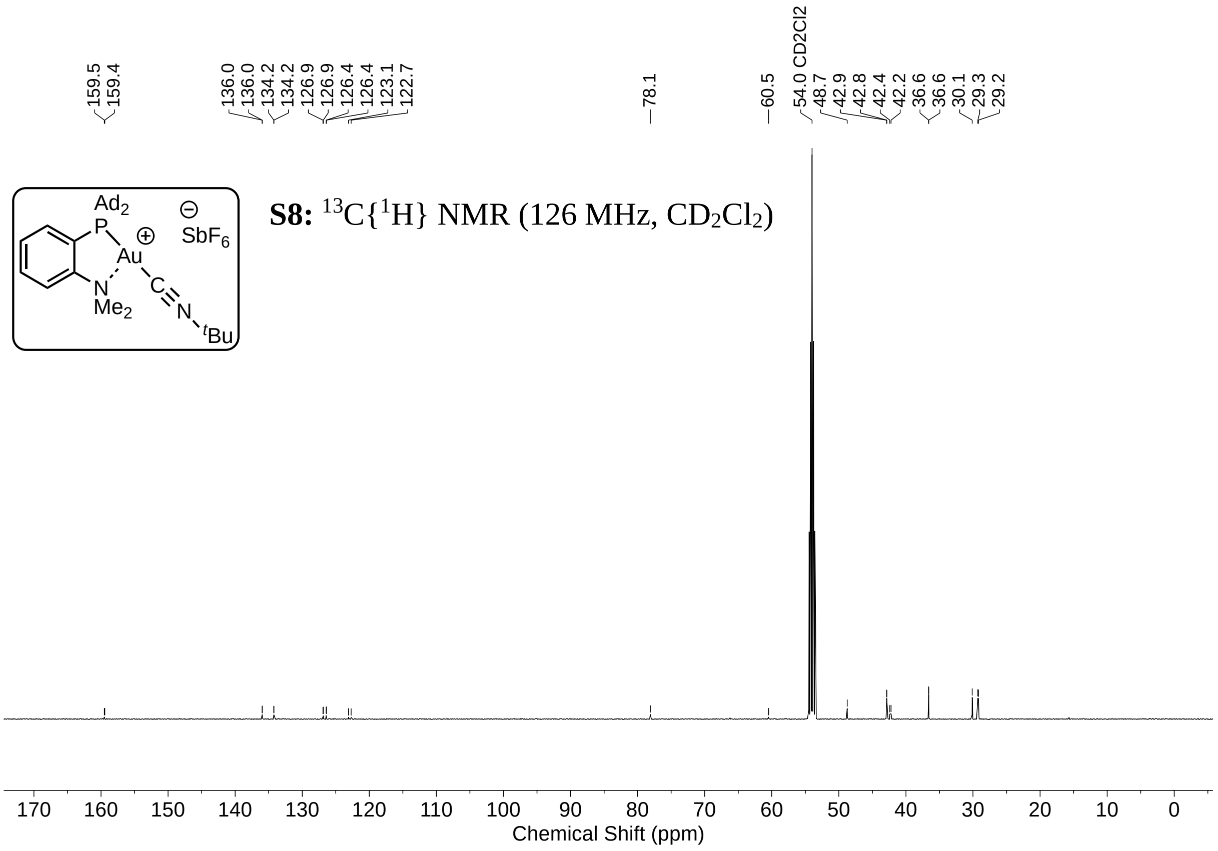

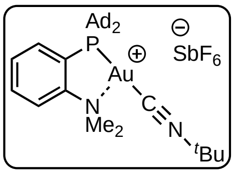

S8: $^{31}P\{^{1}H\}$ NMR (162 MHz, CD_2Cl_2)

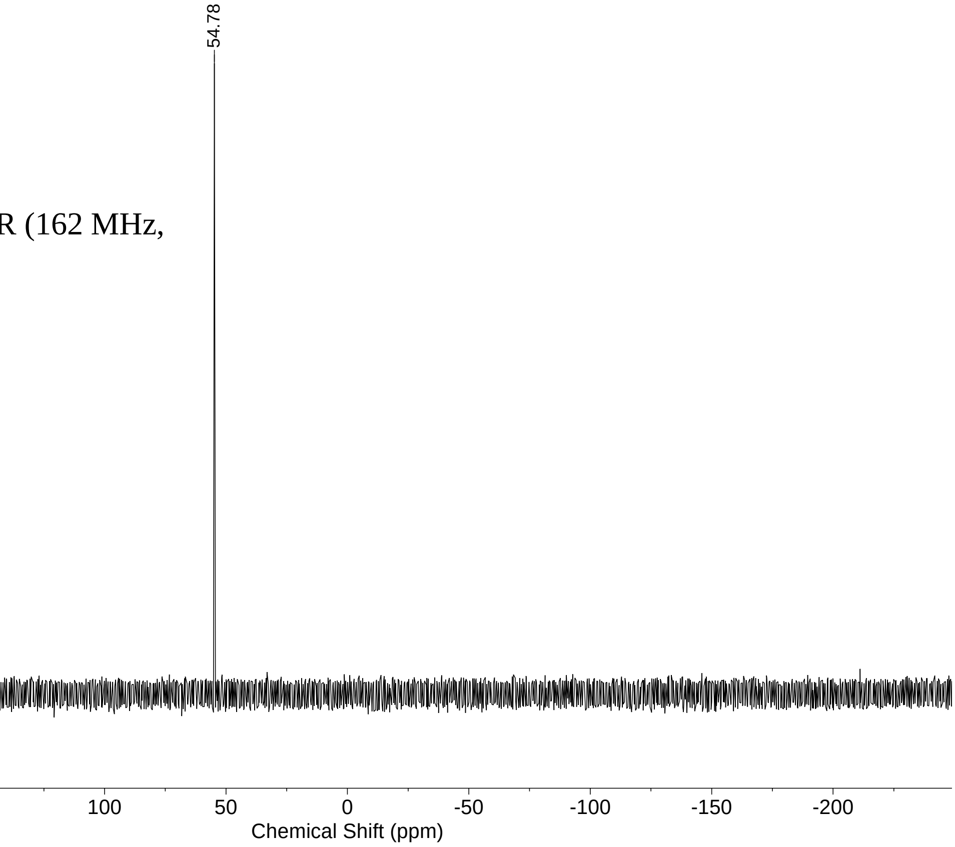

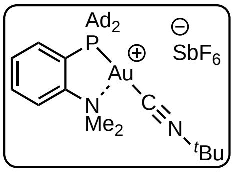

S8: FTIR (ATR, neat)

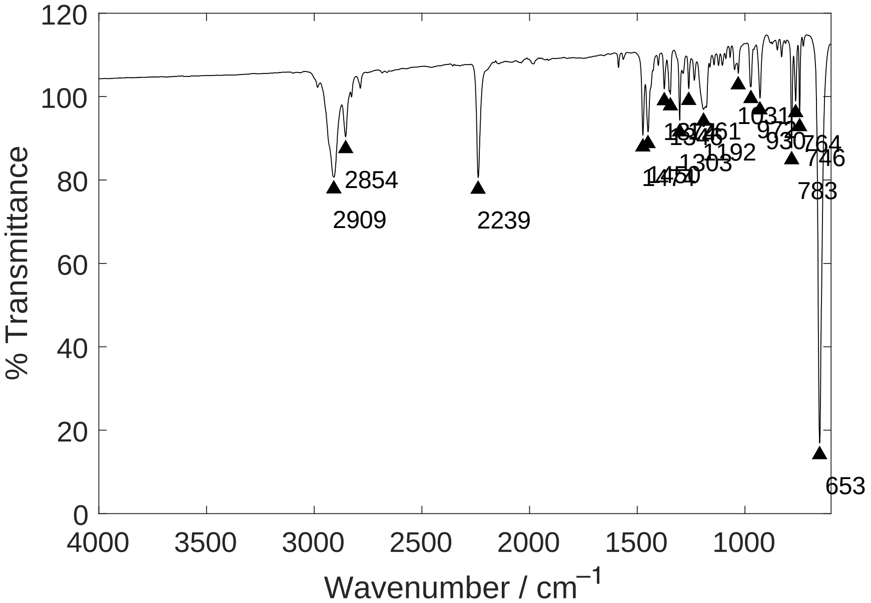

12: ^{1}H NMR (500 MHz, CD_2Cl_2)

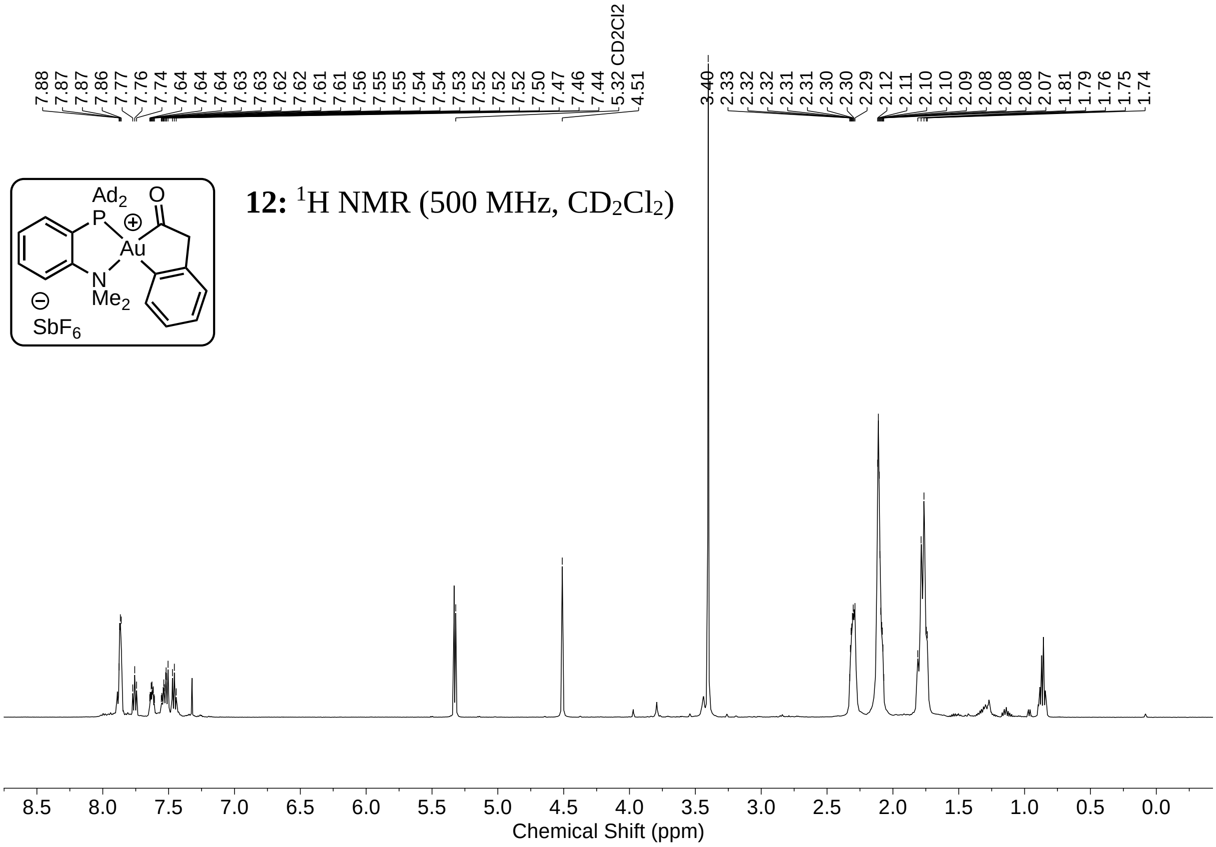

12: ^{13}C{^{1}H} NMR (126 MHz, CD_2Cl_2)

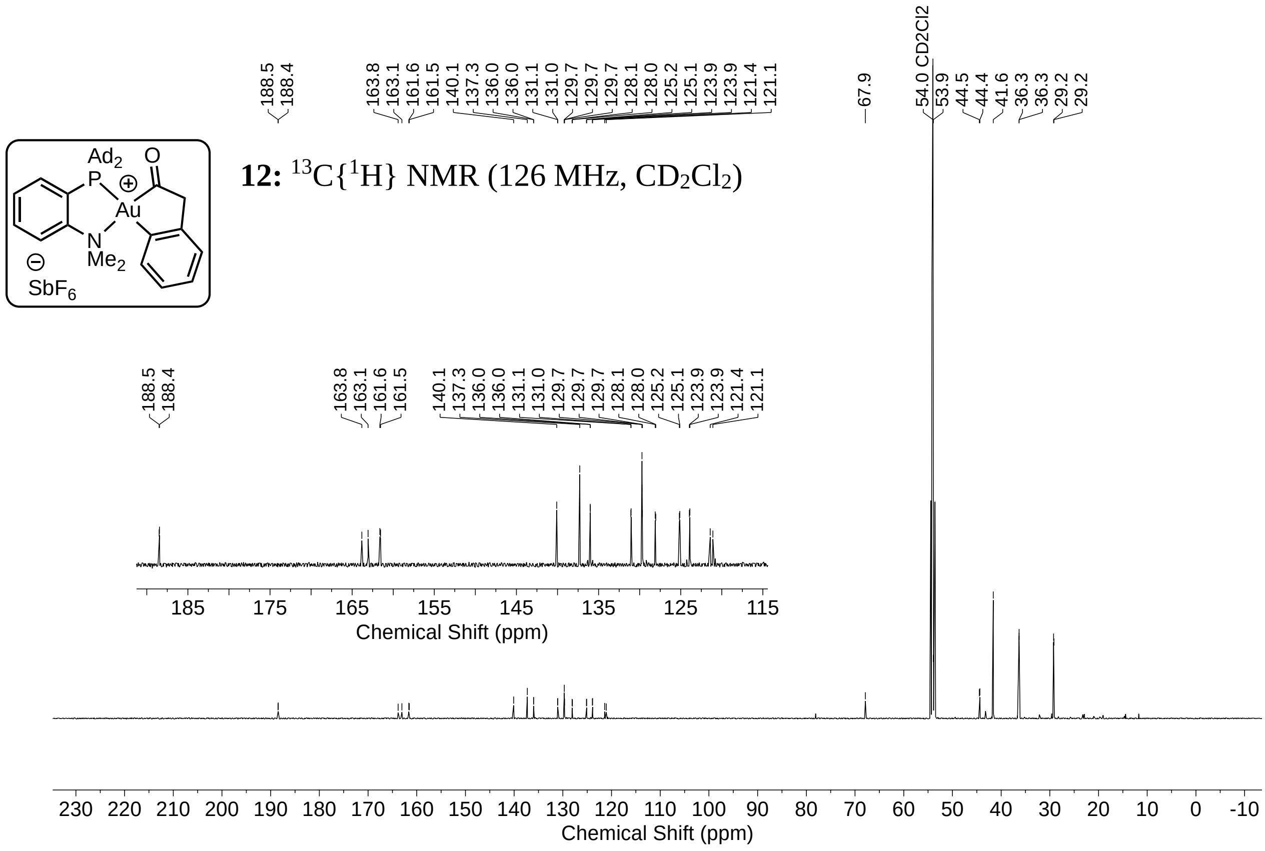

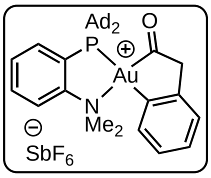

12: $^{31}P\{^{1}H\}$ NMR (162 MHz, CD_2Cl_2)

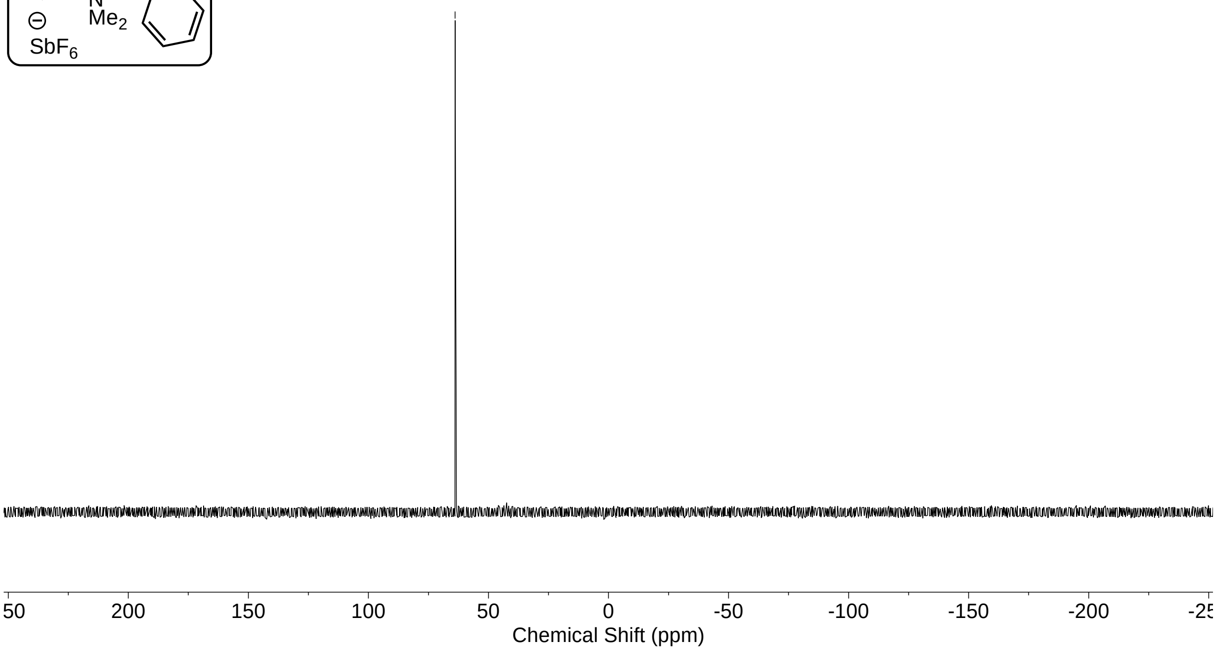

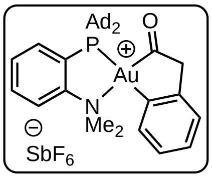

12: FTIR (ATR, neat)

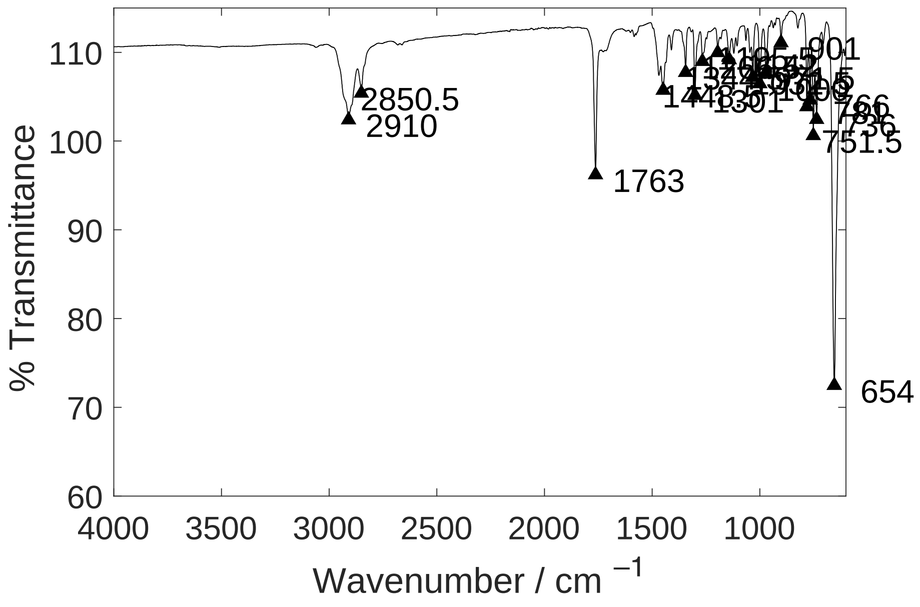

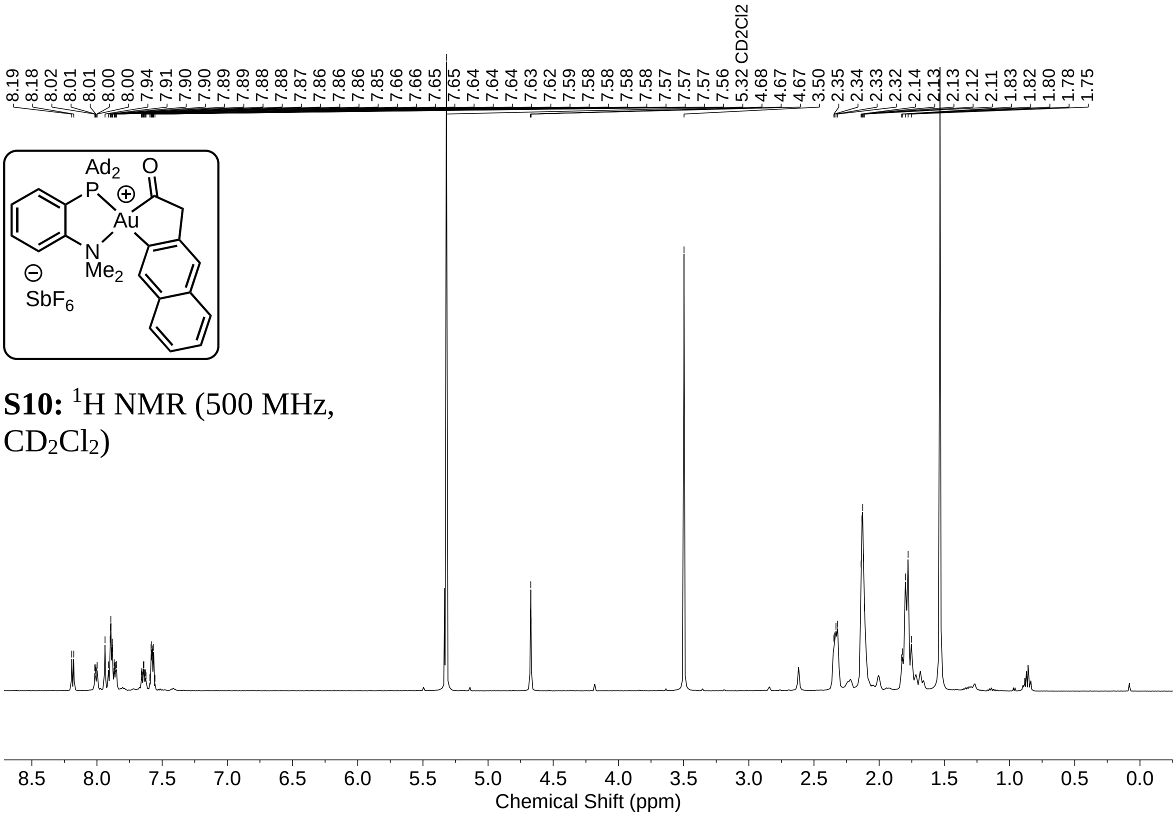

S10: ^{1}H NMR (500 MHz, CD_2Cl_2)

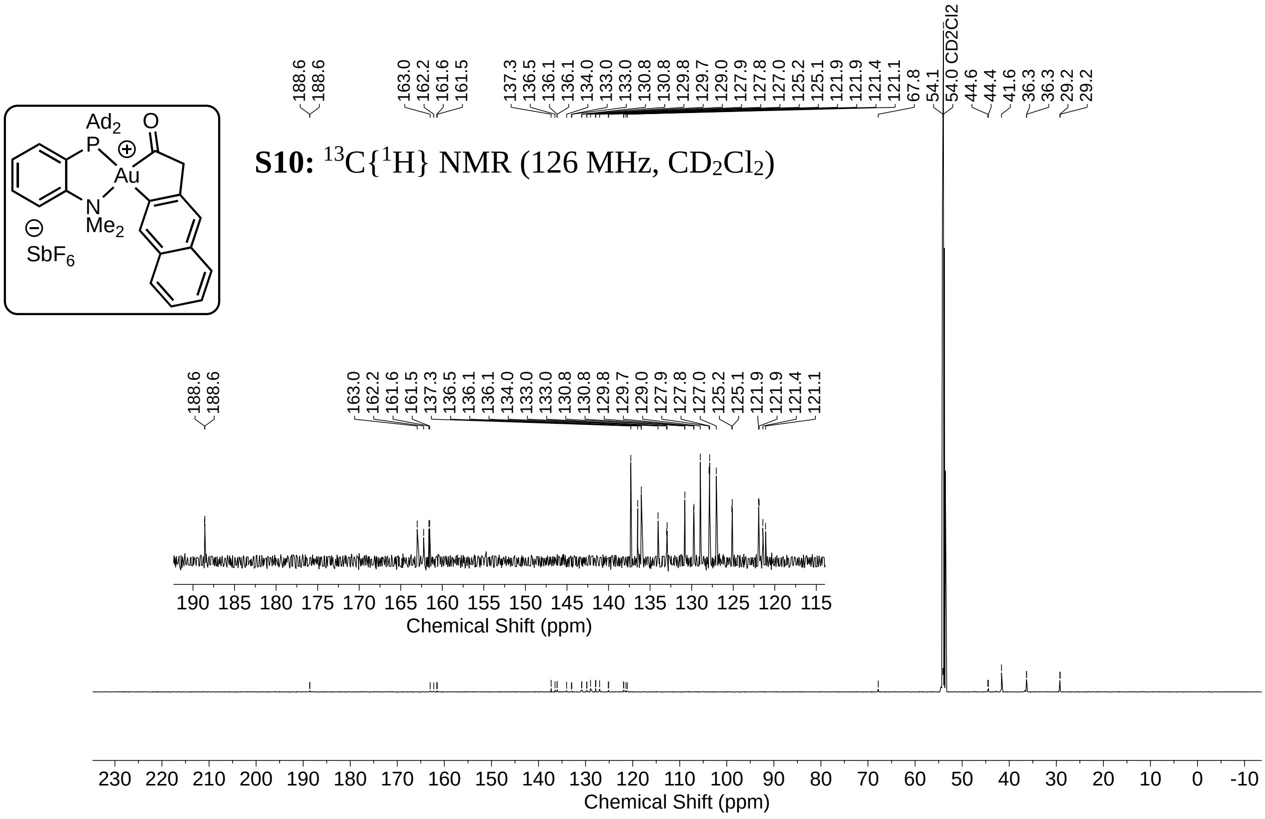

S10: ^{13}C{^{1}H} NMR (126 MHz, CD_2Cl_2)

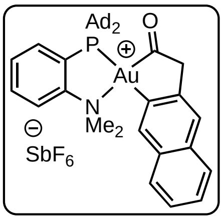

S10: $^{31}P\{^{1}H\}$ NMR (122 MHz, CD_2Cl_2)

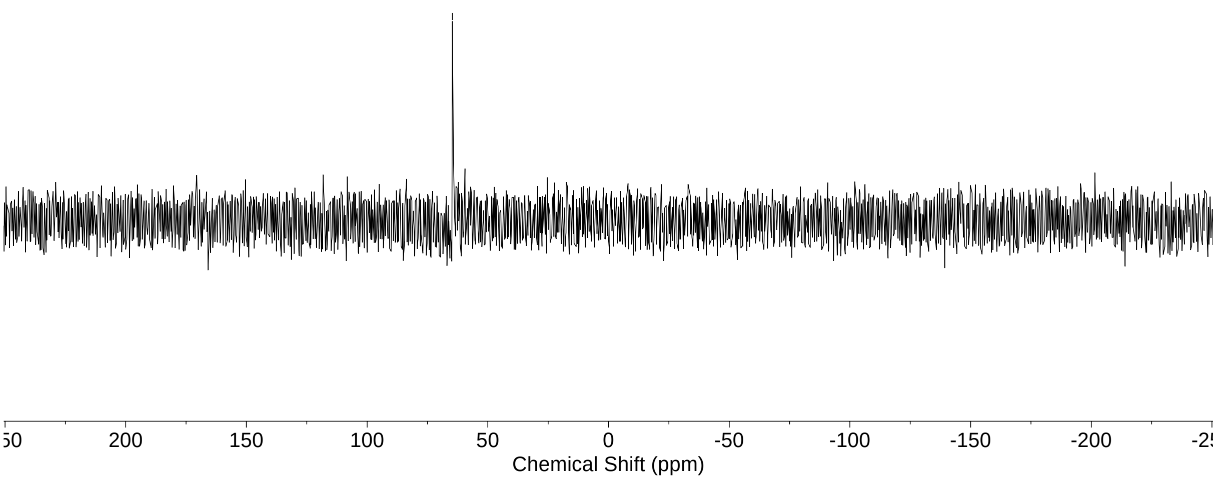

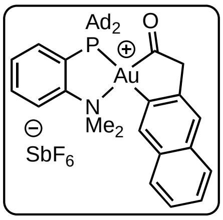

S10: FTIR (ATR, neat)

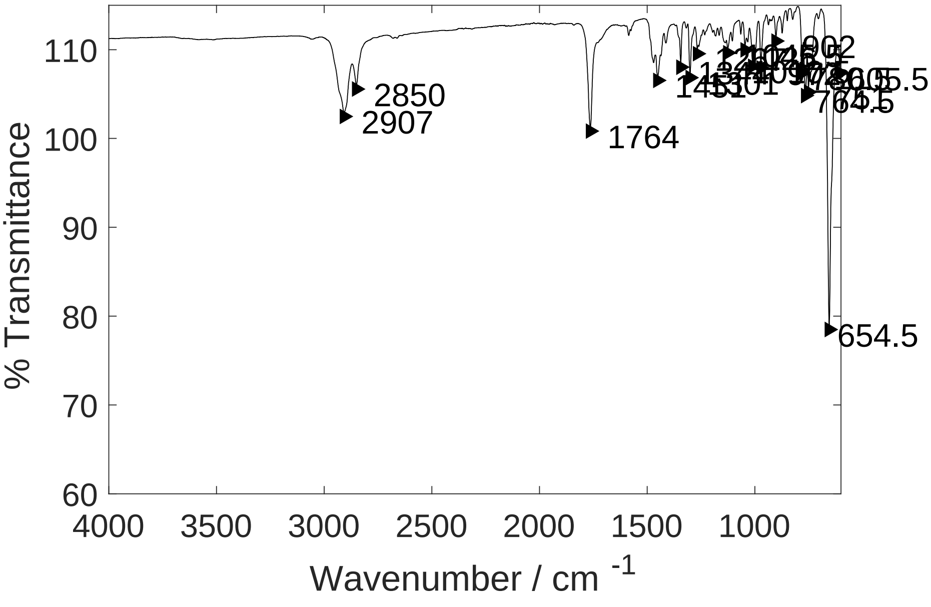

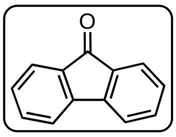

4: ^{1}H NMR (500 MHz, CD_2Cl_2)

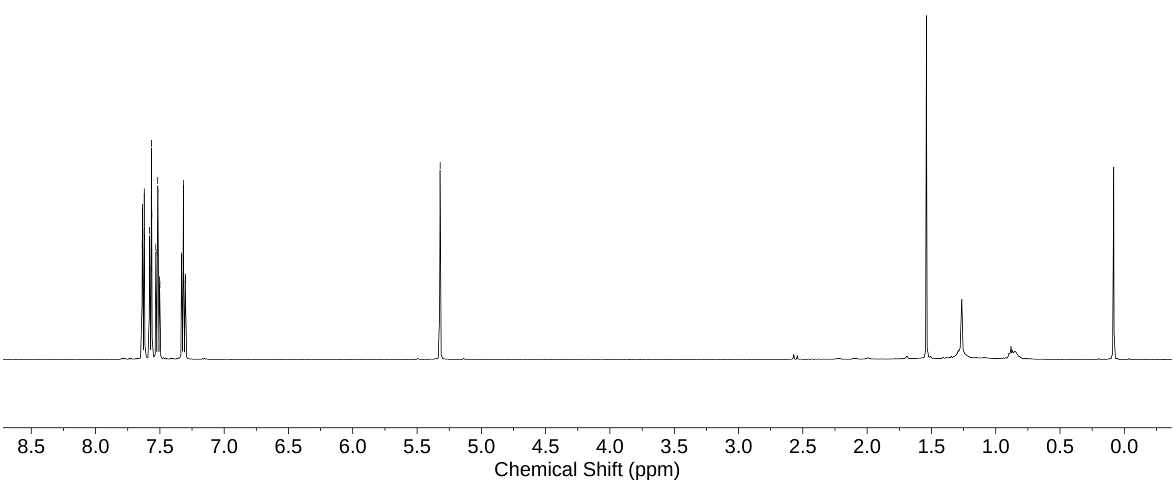

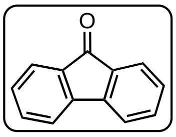

4: ^{13}C{^{1}H} NMR (126 MHz, CD_2Cl_2)

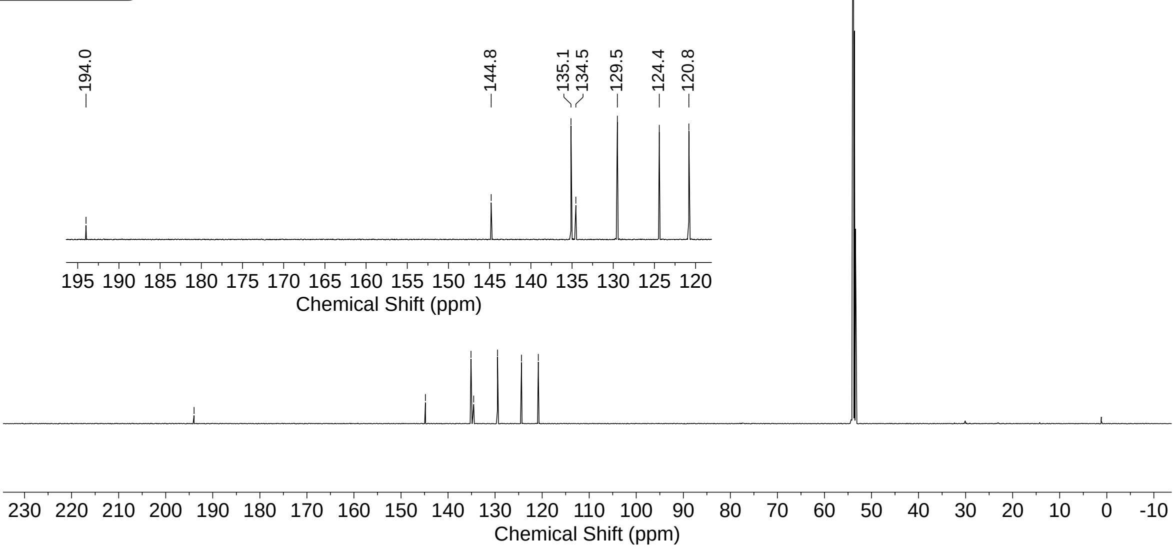

7. References

(1) Pangborn, A. B.; Giardello, M. A.; Grubbs, R. H.; Rosen, R. K.; Timmers, F. J. *Organometallics* **1996**, *15*, 1518-1520.

(2) Kirschner, S.; Uecker, I.; Bolte, M.; Lerner, H.-W.; Wagner, M. *Organometallics* **2019**, *38*, 2818-2823.

(3) Rubina, M.; Rubin, M. *Chemistry of Heterocyclic Compounds* **2012**, *48*, 807-821.

(4) Li, H.; Praveen Rao, P. N.; Habeeb, A. G.; Knaus, E. E. *Drug Dev. Res.* **2002**, *57*, 6-17.

(5) Stafford, J. A.; McMurry, J. E. *Tetrahedron Lett.* **1988**, *29*, 2531-2534.

(6) Dieskau, A. P.; Holzwarth, M. S.; Plietker, B. *J. Am. Chem. Soc.* **2012**, *134*, 5048-5051.

(7) Nappi, M.; Bergonzini, G.; Melchiorre, P. *Angew. Chem. Int. Ed.* **2014**, *53*, 4921-4925.

(8) Ueta, Y.; Mikami, K.; Ito, S. *Angew. Chem. Int. Ed.* **2016**, *55*, 7525-7529.

(9) Zhang, C.; Li, F.; Yu, Y.; Huang, A.; He, P.; Lei, M.; Wang, J.; Huang, L.; Liu, Z.; Liu, J.; et al. *J. Med. Chem.* **2017**, *60*, 3618-3625.

(10) He, Y.; Yuan, C.; Jiang, Z.; Shuai, L.; Xiao, Q. *Org. Lett.* **2019**, *21*, 185-189.

(11) Harper, M. J.; Arthur, C. J.; Crosby, J.; Emmett, E. J.; Falconer, R. L.; Fensham-Smith, A. J.; Gates, P. J.; Leman, T.; McGrady, J. E.; Bower, J. F.; et al. *J. Am. Chem. Soc.* **2018**, *140*, 4440-4445.

(12) Synthesised following a literature procedure, see ref 11.

(13) Prepared according to a literature procedure, see: Hooper, T.; Butts, C.; Green, M.; Haddow, M.; McGrady, J.; Russell, C. A., Chem. Eur. J. 2009, 15, 12196-12200.

(14) de Orbe, M. E.; Echavarren, A. M. *Org. Synth* **2016**, *93*, 115-126.

(15) (a) Cadge, J. A.; Sparkes, H. A.; Bower, J. F.; Russell, C. A. *Angew. Chem. Int. Ed.* **2020**, *59*, 6617-6621. (b) Cadge, J. A.; Bower, J. F.; Russell, C. A. *Angew. Chem. Int. Ed.* **2021**, *60*, 24976-24983.

(16) Wu, C.-Y.; Horibe, T.; Jacobsen, C. B.; Toste, F. D. *Nature* **2015**, *517*, 449-454.

(17) Zeineddine, A.; Estévez, L.; Mallet-Ladeira, S.; Miqueu, K.; Amgoune, A.; Bourissou, D. *Nat. Commun.* **2017**, *8*, 565.

(18) Navarro, M.; Toledo, A.; Mallet-Ladeira, S.; Sosa Carrizo, E. D.; Miqueu, K.; Bourissou, D. *Chem. Sci.* **2020**, *11*, 2750-2758.

(19) Seo, S.; Slater, M.; Greaney, M. F. *Org. Lett.* **2012**, *14*, 2650-2653.

(20) Zhukhovitskiy, A. V.; Kobylianskii, I. J.; Wu, C.-Y.; Toste, F. D. *J. Am. Chem. Soc.* **2018**, *140*, 466-474.

(21) Lundgren, R. J.; Sappong-Kumankumah, A.; Stradiotto, M. *Chem. Eur. J.* **2010**, *16*, 1983-1991.

(22) (a) Bauer, A.; Schneider, W.; Schmidbaur, H. *Inorganic Chemistry* **1997**, *36*, 2225-2226. (b) J. Mathieson, T.; G. Langdon, A.; B. Milestone, N.; K. Nicholson, B. *J. Chem. Soc., Dalton Trans.* **1999**, 201-208. (c) Singa, T. P.; DiPasquale, A. G.; Rheingold, A. L.; Kubiak, C. P. *Acta Crystallogr. Sect. E* **2008**, *64*, m1221. (d) Chow, A. L.-F.; So, M.-H.; Lu, W.; Zhu, N.; Che, C.-M. *Chem. Asian J.* **2011**, *6*, 544-553. (e) Schneider, D.; Schuster, O.; Schmidbaur, H. *Organometallics* **2005**, *24*, 3547-3551.

(23) *SAINT+ v8.38A Integration Engine, Data Reduction Software*; Bruker Analytical X-ray Instruments Inc.: Madison, WI, U.S.A., 2015. (accessed.

(24) *SADABS 2014/15, Bruker AXS area detector scaling and absorbtion correction*; Bruker Analytical X-ray Instruments Inc.: Madison, WI, U.S.A., 2014/15.

(25) *TWINABS 2012/1*; Bruker Analytical X-ray Instruments Inc.: Madison, WI, U.S.A., 2012.

(26) Sheldrick, G. *Acta Crystallogr., Sect. A* **2015**, *71*, 3-8.

(27) Sheldrick, G. *Acta Crystallogr., Sect. C* **2015**, *71*, 3-8.

(28) Dolomanov, O. V.; Bourhis, L. J.; Gildea, R. J.; Howard, J. A. K.; Puschmann, H. *J. Appl. Crystallogr.* **2009**, *42*, 339-341.

(29) Raiford, D. S.; Fisk, C. L.; Becker, E. D. *Anal. Chem.* **1979**, *51*, 2050-2051.

(30) *Gaussian 09 Rev. D.01*; Wallingford, CT, 2009. (accessed.

(31) Chai, J.-D.; Head-Gordon, M. *Phys. Chem. Chem. Phys.* **2008**, *10*, 6615-6620.

(32) Weigend, F.; Ahlrichs, R. *Phys. Chem. Chem. Phys.* **2005**, *7*, 3297-3305.

(33) (a) Andrae, D.; Häußermann, U.; Dolg, M.; Stoll, H.; Preuß, H. *Theor. Chim. Acta* **1990**, *77*, 123-141. (b) Peterson, K. A.; Figgen, D.; Goll, E.; Stoll, H.; Dolg, M. *J. Chem. Phys.* **2003**, *119*, 11113-11123. (c) Pritchard, B. P.; Altarawy, D.; Didier, B.; Gibson, T. D.; Windus, T. L. *J. Chem. Inf. Model.* **2019**, *59*, 4814-4820.

(34) Marenich, A. V.; Cramer, C. J.; Truhlar, D. G. *J. Phys. Chem. B* **2009**, *113*, 6378-6396.

(35) Roşca, D.-A.; Fernandez-Cestau, J.; Morris, J.; Wright, J. A.; Bochmann, M. *Science Advances* **2015**, *1*, e1500761.

(36) Gaggioli, C. A.; Belpassi, L.; Tarantelli, F.; Belanzoni, P. *Chem. Commun.* **2017**, *53*, 1603-1606.
